# Teaching and learning clinical reasoning skill in undergraduate medical students: A scoping review

**DOI:** 10.1371/journal.pone.0309606

**Published:** 2024-10-16

**Authors:** Somayeh Delavari, Farzaneh Barzkar, Remy M. J. P. Rikers, Mohammadreza Pourahmadi, Seyed Kamran Soltani Arabshahi, Abbasali Keshtkar, Helen Dargahi, Minoo Yaghmaei, Alireza Monajemi

**Affiliations:** 1 Center for Educational Research in Medical Education, Medical Education Department, School of Medicine, Iran University of Medical Sciences, Tehran, Iran; 2 Roosevelt Center for Excellence in Education, University College Roosevelt, Utrecht University, Middelburg, The Netherlands; 3 Rehabilitation Research Center, Department of Physiotherapy, School of Rehabilitation Sciences, Iran University of Medical Sciences, Tehran, Iran; 4 Department of Health Sciences Education Development, School of Public Health, Tehran University of Medical Sciences, Tehran, Iran; 5 Department of Obstetrics and Gynecology, School of Medicine, Shahid Beheshti University of Medical Sciences, Tehran, Iran; 6 Department of Philosophy of Science, Institute for Humanities and Cultural Studies, Tehran, Iran; FMUP: Universidade do Porto Faculdade de Medicina, PORTUGAL

## Abstract

**Background:**

Clinical reasoning involves the application of knowledge and skills to collect and integrate information, typically to arrive at a diagnosis, implement appropriate interventions, solve clinical problems, and improve the quality of health care and patient outcomes. It is a vital competency that medical students must acquire, as it is considered the heart of medicine.

**Purpose:**

This scoping review aimed to identify and summarize the existing literature on learning and teaching strategies for improving clinical reasoning skill in undergraduate medical education.

**Methods:**

We conducted electronic searches in Scopus, PubMed/Medline (NLM), Web of Science (WOS), and ERIC to retrieve articles published between January 1, 2010, and March 23, 2024. We also performed hand searches by scanning the reference lists of included studies and similar reviews and searching three key journals. After removing duplicates, two reviewers independently extracted data from primary articles using a standard data extraction form. The authors used Arksey and O’Malley’s framework.

**Results:**

Among the 46581 retrieved records, 54 full-text articles were included in the present review. We categorized the educational strategies based on their aspects, focus, and purpose. Included studies used various educational strategies for improving clinical reasoning skill in undergraduate medical education by serial cue or whole clinical cases that presented as process-oriented or knowledge-oriented.

**Conclusion:**

This scoping review investigated various dimensions of educational intervention for improving clinical reasoning skill in undergraduate medical education. There is a need for more precision studies with larger sample sizes, designing studies according to randomized controlled trials standards, determining MCID, or performing meta-analyses to acquire robust and conclusive results.

## Introduction

Clinical reasoning is the heart of medicine, and the lack of this skill correlates with increased medical errors, decreased patient safety and inadequate patient care [1, 2]. Thus, the development of this competency is one of the most pivotal objectives of medical education [3]. Clinical reasoning is a multidimensional, complex, and integrated skill [4] that plays an important role in the clinical decision making and accurate diagnosis [5]. It relies on an integrated and organized medical knowledge base and extensive clinical experience [6]. Because of the low variety of hospitalized patients, variable quality of supervision, and irregular feedback, clinical rotations alone cannot improve clinical reasoning skill [3, 5]. Medical students at different levels of medical education encounter different medical problems that lead to need for educational support according to their knowledge and experience levels [3].

The existing literatures present a variety of teaching and learning approaches of clinical reasoning that differ in several dimensions. A published narrative review by Schmidt and colleagues [3] shows that researchers have used various strategies to teach clinical reasoning (*eg*, case-based discussion [7], reflection [8, 9], simulation manikins [10], self-explanation [11], *etc*); In this study, the review was limited to articles from Web of Science and PubMed until June 2014. Lubarsky et al. [12] published a narrative study that identified some teaching strategies based on script theory in guiding reasoning during clinical encounters; They proposed some strategies for aligning teaching practices with basic principles of the script theory in classrooms and clinical settings [12]. Furthermore, a narrative review study by Norman [13] shows that acquiring knowledge, deliberate practice, and educational strategies are essential in improving medical students’ clinical reasoning skill. However, these two studies did not conduct a systematic search in academic databases and did not perform a systematic literature review. Hawks et al. [14] did perform a scoping review describing various dimensions of clinical reasoning curricula in undergraduate medical education. Vergel et al. [15] also published a scoping review to identify curriculum and pedagogical practices for improving medical students’ clinical reasoning skill. However, this study is limited to final-year medical students and did not consider another level of undergraduate medical education. In addition, the search was limited to papers published from 2014 to 2018. Although review studies have been published about clinical reasoning teaching, they did not address teaching and learning clinical reasoning skill at different levels of undergraduate medical students by a systematic investigation. Richmond et al. [16] published a realist review to delve deeper into the mechanisms different interventions aid in the development of analytical and non-analytical clinical reasoning. Given their focus on the mechanisms rather than effectiveness, they included 28 studies with varying designs via a structured search of MEDLINE, PsycINFO, ERIC and CINAHL. Although this review identified educational intervention for developing analytical and non-analytical clinical reasoning skill amongst medical students and when and why are they effective, for whom and in what circumstances; but it does not investigate various dimensions of clinical reasoning interventions such as the details of teaching approaches and the types of cases used and the variety of undergraduate medical education systems that tend as the setting of the interventions. Cooper et al. [17] conducted a literature review in 2021 to identify successful teaching strategies in improving clinical reasoning skill in medical students and developed a consensus statement accordingly. They identified 27 eligible studies via their structured search of MEDLINE, PsycINFO, CINAHL, EMBASE, ERIC and Google Scholar for articles published in the past 30 years. They emphasized a lack of effectiveness for teaching the general thinking processes involved in clinical reasoning, and the effectiveness of specific teaching strategies aimed at building knowledge and understanding. Although this review identified educational strategies for developing clinical reasoning skill among medical students; it did not address various aspects of clinical reasoning interventions that may affect its effectiveness and the variety of the effects in different levels of undergraduate medical trainees and different educational systems.

Despite the importance of clinical reasoning teaching and learning in undergraduate medical education, there is no consensus among empirical studies on the effective approaches. Scoping reviews are a form of evidence synthesis that is performed based on an exploratory research question often aiming to approach broad, fragmented, or complicated special field of research that have not received much attention in the literature [18, 19]; Scoping review has important role in hypothesis generation [19]. Scoping review studies examine the extent (that is, size), range (variety), and nature (characteristics) of the evidence on a research topic or question; determine the value of conducting a systematic review; summarize evidence that is heterogeneous in methods or discipline; or identify literature gaps in the literature to aid the planning and commissioning of future research [20]. Since current reviews on teaching and learning clinical reasoning lacked a rigorous methodology, a comprehensive search, and solid and actionable findings, we conducted this scoping review in a systematic way to identify and describe available educational strategies to enhance clinical reasoning in undergraduate medical students at different levels of their training and in different educational settings; This scoping review presents several aspects of educational strategies accessible for clinical teachers and researchers and clarifies the empirical support and their usefulness based on each included study. We have also endeavored to make an empirically tested collection of educational strategies accessible to clinical teachers and researchers. Therefore, the primary outcome of this scoping review was to identify the range, extent, and nature of the educational approach for teaching clinical reasoning in undergraduate medical education. The secondary outcome of this scoping review was determining the feasibility of conducting a systematic review to investigate the effectiveness of the educational strategies to teach clinical reasoning in undergraduate medical education. The third outcome was summarizing and disseminating research characteristics about educational strategies for teaching clinical reasoning in undergraduate medical education and identifying educational interventions for increasing diagnostic accuracy or clinical reasoning performance according to calculated effect sizes by extracted data from included studies.

## Materials and methods

The steps of this scoping review are based on Arksey and O’Malley’s framework for scoping reviews [21]. We used the PRISMA (Preferred Reporting Items for Systematic Reviews and Meta-Analyses); extension for scoping reviews (PRISMA-ScR) [20] and The PRISMA 2020 [22] for reporting our scoping review. The protocol of the present study was registered at https://osf.io/6ka48/ by DOI: https://doi.org/10.17605/osf.io/9vaer.

### Identifying the research question

1. What educational strategies are used to enhance clinical reasoning skill in medical students?
2. Is there a suitable condition for conducting a complete systematic review of the educational approach to teach clinical reasoning in undergraduate medical education?
3. What educational interventions can help increase diagnostic accuracy in undergraduate medical education?

### Identifying relevant studies

CIAO (**C** (**C**lient/ population), **I** (**I**ntervention), **A** (**A**lternative intervention/comparison), **O** (**O**utcome)) format [23] was used for formulating a search strategy for the current scoping review (S1 File).

#### Licensed journal databases

S.D. performed electronic searches in the following databases between January 1, 2010, and March 23, 2024: PubMed/Medline (NLM), Scopus, Web of Science (WOS), and ERIC. The syntax of this scoping review (designed by S.D. and M.P. and confirmed by A.K and R. M.J.P. R.) is a combination of Medical Subject Headings (MeSH; MEDLINE), Emtree medical (Embase) terms, and free text words in research equations with “OR” and “AND.” Boolean operators were used. Free text words were also selected from the synonyms of all keywords used in the text of relevant studies. The search strategy was initially created in PubMed/MEDLINE (NLM) and then translated into other databases (S1 File). We did not search ongoing, unpublished studies and gray literature such as books, theses, conference proceedings/abstracts, and news/magazine articles.

#### Hand searching

Manual searches (performed by S.D. and M.P. independently) included scanning reference lists of included studies, similar reviews, and three key journals (BMC Medical Education, Medical Education, and Advances in Health Sciences Education).

#### Study selection

After completing all database searches, the citations were imported into the EndNote reference manager software (Version X9.1; Clarivate Analytics Inc), where duplicate citations were removed automatically and double-checked manually. Articles were assessed for inclusion through a 3-stage screening process.

The first stage was screening the titles and abstracts to determine studies eligible for inclusion (S1 Table), in this stage, all studies divided in 2 parts and investigated independently by two reviewers (S.D. and M.P.) in two steps; The first step was investigation of titles retrieved with systematic searches in academic databases. The article was included in this first screen if the title identified clinical reasoning or medical education related terms. Then, we reviewed the abstracts of all potentially eligible articles. Then, two reviewers (S.D. and F.B.) independently evaluated the full text of potentially relevant nonduplicated articles. Conflicts were resolved by discussion to reach a consensus. A third reviewer (M.Y.) acted as an arbitrator when agreement was not reached. We described the number of primary studies included and excluded in each stage of the selection process using the PRISMA-2020. There are inclusion and exclusion criteria in (S1 Table).

### Data extraction & data synthesis

At first, the data extraction tool was piloted with three articles of varied methodological approaches to ensure it would collect correct and effective information. This process was verified by one researcher (S.K.S.A). Then, two reviewers (S.D. and F.B.) independently extracted data from primary articles by extraction form. Any discrepancies were resolved same as screening stage. Data charted and reported based on Arksey and O’Malley’s framework for scoping reviews [21].

### Level of evidence and critical appraisal

Two reviewers (S.D. and H.D.) assessed the level of evidence (S2 Table) according to the Australian National Health and Medical Research Council (NHMRC) classification for intervention studies [24]. Conflicts were resolved by discussion to reach a consensus. A third reviewer (A.M.) acted as an arbitrator when consensus was not reached.

In this scoping review, the Mixed Methods Appraisal Tool (MMAT) was used to assess the quality of the included studies. The MMAT is a comprehensive tool that can be applied to a variety of study designs, including qualitative, quantitative, and mixed-methods research [25, 26]. Quantitative studies were evaluated as two general categories (randomized and nonrandomized) and were evaluated according to their specific criteria, which are similar to the Cochrane tools. Two reviewers (S.D. and F.B.) independently evaluated each study using the relevant MMAT criteria based on the study design. Any discrepancies between reviewers were resolved through discussion until consensus was reached. The MMAT quality appraisal results were used to provide an overview of the methodological strengths and weaknesses of the included studies.

### Collating, summarizing, and reporting the results

A narrative synthesis was planned. We did not anticipate a meta-analysis being possible as homogeneity may not be consistent. We structured the narrative according to the intervention and all possible outcomes. This study employed a descriptive-analytical method within the narrative tradition to summarize the data. For the statistical analysis, we used mean (SD), mean difference (MD) (if it was reported), standardized mean difference (SMD), and *P* value. All SMD were calculated (https://www.psychometrica.de/effect_size.html) and interpreted according to S3 Table. There is a relationship between effect size and study power. The power of a study is determined by the sample size, effect size, and type I error probability. Researchers can increase power by increasing the sample size, reducing score variance, and selecting a higher Type I error probability. However, a higher Type I error probability should only be used with good justification. Researchers should prospectively estimate sample size and power, which usually targets the smallest educationally important difference. A narrower confidence interval indicates higher precision of study results [27].

## Results

### Publication pattern, papers design, and participants

From the 46581 identified records, we included 307 full-text reports. A total of 253 studies were excluded after evaluating their full text (S2 File). Overall, data were extracted from 54 articles (S1 Fig).

The included studies were published in 33 different journals. The three journals that published the highest number of studies: BMC Medical Education, Medical education and Advances in Health Sciences Education published 39% of the included articles. The number of published articles per year increased from zero in 2013 to nine in 2019 (S2 Fig, S4 Table). 38 (70%) articles were published by authors from five countries of Germany (17 articles) [28–44], Iran (6 articles) [5, 45–48], USA (5 articles) [49–53], Netherland (5 articles) [9, 54–57], and Brazil (5 articles) [58–62] (S4 Table).

All details of publication patterns of included studies are in supplement (S4 Table). S5 Table shows that the original studies’ participants varied from 12 to 398, with a median of 72 (interquartile range, 44–96); 81% of studies had ≤100 participants.

All of the studies except 14 studies [5, 28, 31, 34, 36, 37, 42, 47, 48, 52, 53, 56, 63, 64] were performed by randomized controlled trials (RCT) design; 11 and 6 of them were RCT add-on trials [6, 41, 43, 46, 49, 50, 61, 62, 65–67], and cross over [30, 35, 44, 45, 68, 69], respectively. In addition, 61% of studies (33 papers) had 2 arms of intervention and comparison [29–31, 33–37, 43–47, 49–52, 56, 57, 60–62, 64, 66–74]; 7 studies [5, 28, 42, 46, 48, 53, 63], 12 studies [4, 6, 9, 38, 39, 41, 54, 55, 58, 59, 65, 75], and 2 study [32, 40] had 1, 3, and 4 arms, respectively. 22 studies (41%) were conducted on internal medicine departments [4, 6, 9, 29, 34, 35, 37, 38, 45, 49, 51, 52, 54, 55, 57, 59, 60, 62, 65, 66, 69, 72, 76] (S5 Table).

### Description of academic medical education duration and participants academic years

S5 Table shows the characteristics of Participants of most papers (23 papers) were fourth-year medical students only [5, 9, 35, 37, 44, 45, 49, 53, 54, 61, 62, 70, 74, 77] or in combination with other groups of medical students [28–31, 33, 36, 40, 41, 68]. Undergraduate medical education duration was different from 4 years [4, 6, 65, 72, 75] to 7 years [5, 45–48, 64].

### Investigating the conducting a complete systematic review and meta-analysis

Included studies used different clinical cases/topics for four phases of pretest, intervention, posttest, and follow-up (S6 Table and S3 File). The most common educational strategies in intervention group include self-explanation [6, 32, 62, 65, 72], reflection [5, 9, 32, 41, 54–56, 58–61, 70, 77], test enhanced learning [5, 35, 37, 44, 62, 71], Team Based Learning [30, 31, 68, 70], thinking aloud [5, 45, 74], and PBL [34, 43, 47, 63, 66, 67]. On the other hand, The most common educational strategies in comparison/s group include traditional teaching [43, 46, 67, 70, 75, 77, 78], self-explanation [6, 32, 65, 73, 76], solving word puzzles [4, 54, 65], reflection [32, 41, 55, 58–60], feedback [6, 32, 35, 40, 71], and test enhanced learning [37, 44, 62, 71]. All of these educational strategies in intervention and comparison/s groups used individually and in different combinations educational strategies. Method of clinical case presentation in 28 and 13 studies were whole cases [4–6, 9, 34, 35, 37–40, 42, 45, 54–62, 65, 67–69, 72, 74, 77] and serial cue [29, 32, 36, 41, 43, 44, 46–48, 53, 63, 70, 71], respectively (S7 Table and S3 File). Included studies used various types of questions in pretest, posttest, and follow-up (S8 Table and S3 File).

### Description of educational interventions

S6 and S7 Tables show the characteristics of an educational intervention for improving clinical reasoning skill in undergraduate medical students. According to these tables, the duration of intervention varied from 1 hour and 15 minute [6] to 4 weeks [43, 46, 52, 75]/6 weeks [34], 10 sessions [35, 44], 18 days [71], 42 hours [28], and 3 months [37, 47]. Duration of intervention for 12 (24%) studies [4–6, 31, 33, 49, 65, 68, 69, 72, 74, 77] was ≤3 hours. The number of clinical cases/topics varied from 1 [5, 57] to 135 [37]; 34 studies used at least 4 [4, 6, 29–31, 53, 65, 75] clinical cases/topics or more [9, 33–37, 39, 42, 44, 48, 49, 54–56, 58–60, 62, 64, 71–74, 76, 77] (S6 and S7 Tables, and S3 File).

According to S7 Table, 25 (46%) studies used only one educational strategy as intervention [4, 9, 28, 33, 34, 36–39, 48, 49, 51, 53–55, 57–59, 64, 66, 68, 69, 72, 73, 75, 76]. Studies used various educational strategies such as feedback [5, 6, 32, 35, 40, 46, 70, 71, 77], SNAPPS [75], case-based clinical discussion [38, 42, 45, 46, 52], flipped/inverted classroom [28], self-explanation [6, 32, 62, 65, 72], reflection [5, 9, 32, 41, 54–56, 58–61, 70, 77], test enhanced learning [5, 35, 37, 44, 62, 71], TBL [30, 31, 68, 70], thinking aloud [5, 45, 74], and PBL [34, 43, 47, 63, 66, 67] as interventions for improving clinical reasoning skill. Researchers used various educational strategies such as traditional teaching [43, 46, 67, 70, 75, 77, 78], self-explanation [6, 32, 65, 73, 76], solving word puzzles [4, 54, 65], reflection [32, 41, 55, 58–60], feedback [6, 32, 35, 40, 71], and test enhanced learning [37, 44, 62, 71] for comparison groups. Purpose of teaching approach of 34 [5, 9, 29, 32, 34, 36, 40, 41, 43–48, 52–59, 63, 64, 67–71, 73–77] and 13 [4, 6, 31, 33, 35, 38, 42, 50, 60–62, 65, 72] studies were process- and knowledge- oriented, respectively. There is additional data about educational strategies in intervention and comparison groups in S7 Table and S3 File.

Three studies used similar clinical topics/cases for intervention [4, 6, 65]. Four studies used different clinical cases/topics for intervention and comparison groups [34, 56, 60, 71, 77]. Seven studies used similar clinical topics/cases for pretest, intervention, and posttest [4, 28, 32, 39, 40, 46, 53]. Four Studies without pretest and follow-up used similar clinical topics/cases for intervention and posttest [30, 61, 68, 69]. There are more details in S6 and S8 Tables and S3 File.

In summary, there are different educational strategies for teaching and learning clinical reasoning; The researchers used different educational strategies in pure or blended ways. The duration of these interventions was different from 1hrs to 10 weeks (S7 Table and S3 File). The number of clinical topics/cases for educational intervention, pretest, and posttest were different; Some studies used similar numbers and types of clinical topics/cases for these three steps (S8 Table and S3 File).

### Effect of educational interventions on clinical reasoning skill improvement

Clinical reasoning is a mental process that plays an important role in the management of diseases and diagnosis accuracy [5]. This process starts with the chief complaint and ends with the diagnosis and management of the disease [79]. extensive clinical practice and clinical knowledge lead to enriched illness scripts with multiple clinical presentations; therefore, illness script structures are different according to educational levels and the clinical duties of medical students [5]. Medical students’ performance in the whole/any parts of the process of clinical reasoning shows their clinical reasoning performance. On the other hand, diagnostic accuracy shows the medical student’s performance only in correctly diagnosing the disease. Kind of outcomes (clinical reasoning performance/diagnostic accuracy) extracted from the included studies.

The results of a study by Aghili et al. showed that educational intervention (traditional educational programs + simulation + feedback) has a significant and large effect on clinical reasoning skill improvement (MD, 3.82 [2.43–5.19]; SMD, 1.552 [0.928–2.175]; *P* = 0.001) [46]. SNAPSS, compared with traditional clinical teaching, had a statistically significant large effect (SMD, 1.65 [0.932–0.367); *P* = 0.001) on clinical reasoning skill improvement; SNAPPS, compared with OMP, had a statistically meaningful effect on clinical reasoning skill (1.013 [0.355–1.672; *P* = 0.005) [75].

Diagnostic performance was statistically significantly affected by resident self-explanation with prompts in contrast to solving word puzzles (control group) (SMD, 1.601[0.85–2.353]) [4]. The results of Lee et al. showed that lecture in combination with teaching illness script in comparison with self-directed study had a statistically significant and very large effect on the clinical reasoning skill (SMD, 1.241 [0.651–1.831]) [74].

The results of the Choi et al.’s study showed that immediate feedback + reflection in comparison to attended outpatient clinic or combination of outpatient clinic + lecture had a statistically significant and very large effect on diagnostic accuracy in training cases (SMD,1.462 [0.894–2.029] and SMD, 1.237 [0.649–1.825], respectively) [77]. According to the results of Gong et al study, Blended teaching (TBL + feedback + reflection + summarizing key points and commented on students’ performance by teacher) compared with traditional bedside teaching had a statistically significant and very large effect on clinical reasoning performance (SMD, 1.241 [0.136–2.346]; *P* = 0.002) [70].

The findings of Weidenbusch et al. demonstrate that Live-CCD in comparison paper cases had a significant and very large influence on clinical reasoning performance in posttest (SMD, 1.936 [1.337–2.535]; *P* < 0.001) and follow-up (1.933 [1.334–2.532]; *P* < 0.001). Video-CCD in comparison paper cases had a significant and large effect (SMD, 1.108 [0.562–1.654]; *P* < 0.001) in posttest and a powerful and very large impact on clinical reasoning performance in follow-up (SMD, 1.49 [0.916 _2.064]; *P* < 0.001) [38].

S9 Table shows the details of evidence snapshots on the effectiveness of interventions to improve clinical reasoning performance or diagnostic accuracy based on the included studies.

### Discussion

The first aim of the present study was to identify and summarize the existing literature and the types of educational strategies used in clinical reasoning improvement and evaluate the feasibility of a systematic review and meta-analysis based on the results.

The present study included 54 original studies published between 2010 and March 23, 2024. We identified and described available educational strategies to improve clinical reasoning in undergraduate medical education. We identified different dimensions of the educational approach for teaching clinical reasoning in undergraduate medical education. Feedback [5, 6, 32, 35, 46, 70, 71, 77], self-explanation [4, 6, 32, 62, 65, 72, 73, 76], reflection [5, 9, 32, 54–56, 58–60, 70, 77], and test enhanced learning [5, 35, 37, 44, 62, 71] were the most educational strategies used as intervention or comparison for improving clinical reasoning skill in undergraduate medical education.

The second aim of this scoping review was to determine the value of undertaking a systematic review of educational approaches to teach clinical reasoning in undergraduate medical education. Clinical reasoning is a complex cognitive construct requiring different skill sets, and the teaching approaches and the applied interventions are often multifaceted [16]. Moreover, many of the included studies have not adequately addressed the confounding factors, and many studies have used multiple concomitant interventions. Therefore, presenting a clear picture of the relative effectiveness of each unique educational strategy for improving clinical reasoning might be difficult.

Nevertheless, examining the evidence on this topic can provide insight for designing future studies in the field and finding the appropriate ways to synthesize the evidence on this topic. We suggest that it may be possible to conduct systematic reviews to compare knowledge-oriented and thinking process-oriented approaches and serial cue versus whole cases interventions. Because realist reviews allow for considering the various features and complicated character of this phenomenon, we recommend employing them for more complex interventions or parts of complex interventions, such as feedback, rather than traditional systematic reviews. For comparison-free reflection with other interventions, such as cued reflection via subgroup analysis, a meta-analysis may be practical.

Our third aim was to summarize and disseminate research characteristics of the studies on the educational approaches for teaching clinical reasoning in undergraduate medical education and to identify possibly effective educational interventions for increasing diagnostic accuracy in undergraduate medical education. Our scoping review found that SNAPPS was superior to a one-minute preceptor, and both were superior to traditional methods [75]. Blended educational intervention, in comparison with based clinical reasoning discussions had a nonmeaningful and negligible effect on clinical reasoning skill improvement [52]. According to the results of Gong et al.’s study, blended teaching compared with traditional bedside teaching, led to a meaningful and very large change in clinical judgment [70]. Hypothetico-deduction was superior to self-explanation for improving clinical reasoning [73, 76]. Seminar combined with TBL has no meaningful effects on clinical reasoning skill and knowledge acquisition [30]. In contrast, the results of Jost et al. show that TBL led to significant and large changes in students’ clinical decision-making skill [31]. According to the Lee et al.’s findings, lectures that included teaching illness script with lectures led to nonsignificant and ignorable changes in DTI scores but considerable and meaningful changes in CRP scores compared to self-directed study [74]. All these results are inconclusive, and more studies are needed with more sample sizes or meta-analysis for robust and conclusive results.

Immediate reflection led to nonmeaningful and minimal changes in diagnostic accuracy in the control set, while attending an outpatient clinic and attending a lecture led to immediate consideration that led to nonmeaningful and minimal changes in diagnostic accuracy in the control set; these variations in diagnostic precision in training instances were statistically meaningful with very large different [77]. These results are inconclusive, and more research with a larger sample size is required to obtain conclusive evidence. The structured reflection condition compared with the immediate diagnosis condition and the differential diagnosis condition led to meaningful and nonmeaningful changes in scores of clinical diagnoses in the immediate test, respectively. In the delayed test, this change in the score of the two comparison groups was meaningful [9]. Modelled reflection compared with free reflection and cued reflection resulted in considerable changes in diagnostic accuracy [55, 59]; Free reflection compared with modelled reflection led to moderate differences in diagnostic accuracy [55]. The findings of these studies are insignificant, and repeating the present study with a larger sample size or conducting a meta-analysis is required to provide stronger evidence.

In summary, with the increasing rate of research literature, policymakers cannot individually assess all primary research to make appropriate educational decisions [80]. Regarding the feasibility of conduct of a systematic review and metanalysis, one should note that systematic reviews and meta-analyses address a need for policy makers in medical education and health sciences to be able to access relevant, accessible, high-quality evidence, and up-to-date information [80] by integrating high-volume and often conflicting findings on a specific topic [23]. The increasing use of systematic reviews and particularly, the growing reliance on evidence synthesis through the use of rigorous research methods have led to significant advancements in medical education. However, these methods are not feasible in all fields of medical education, particularly in the clinical reasoning domain, due to limitations in the number of similar studies, common research questions, conceptual frameworks, operationally defined interventions, and rigorous, appropriately aligned outcomes [81]. According to the Cochrane handbook, statistical and methodological heterogeneity is one of the important challenges and limitations in conducting systematic reviews and meta-analyses; statistical heterogeneity may be solved by subgroup analysis in meta-analysis, but methodological and conceptual heterogeneity remain a limitation [80].

On the other hand, the results of most of the currently available studies have considerable conceptual heterogeneity, serious methodological limitations and low precision that limit the applicability of the conclusions, and the accuracy of a pooled effect size attained from metanalysis. Since we need more robust evidence to use these educational strategies in undergraduate medical training, a comprehensive search in academic databases and gray literatures might lead to identify more studies to perform a systematic review and meta-analysis or realist review.

Randomized Controlled Trials (RCTs) are widely considered strong study designs, but they present complexity and challenges in medical education research. The highly complex education system may be a poor fit for RCTs. Additionally, the feasibility and applicability of research models derived from clinical research to education studies have been questioned. RCTs and meta-analysis are appropriate for many study goals in medical education, depending on the research question and the presence of compelling strengths and limitations [81]. Thus, we included nonrandomized studies as well as RCTs to widen the applicability of the practical findings. Promoting the Cochrane Collaboration standard for RCTs and controlling confounders in medical education studies, especially in clinical reasoning intervention, poses significant challenges. However, it is feasible for researchers to identify and control some of these confounders. Randomized controlled trials (RCTs) and meta-analysis are suitable for many study goals in medical education, but they also have notable limitations [82].

### Methodological pitfalls in the included studies and future research

Some fundamental methodological problems in the included studies (S9 and S10 Tables) should be considered for future research.

Although most of the included studies were RCTs by design (based on the randomized assignments of students to intervention groups), it was impossible to ensure whether and how random sequence generation had been performed due to suboptimal reporting.

Most studies have utilized p-values to demonstrate that groups were balanced at baseline, but this approach is not recommended for routine assessment [83]. P-values are influenced by sample size and merely indicate the probability that observed differences are due to chance, which can be misleading, especially when the sample size lacks sufficient power to detect significant baseline differences [84].

To improve the evaluation of baseline balance, future researchers should focus on effect size measures [83]. For instance, a standardized mean difference (SMD) of 0.2 indicates a trivial difference, following Cohen’s guidelines: SMD < 0.2 signifies no or trivial effect, 0.2 to 0.5 indicates a small effect, 0.5 to 0.8 a medium effect, and >0.8 a large effect [85]. Additionally, a risk difference (RD) of 0.1 for qualitative data is another useful benchmark [83, 86].

While all of studies report p-values for their outcomes, a significant number fail to provide effect sizes and their confidence interval. Reporting effect sizes along with their confidence intervals offers more informative insights into population predictions than p-values alone [83, 87]. Researchers are encouraged to include confidence intervals for effect sizes to clarify the precision of their findings alongside p-values and effect sizes that ignore precision.

Furthermore, the analysis and reporting of covariates or confounders are essential components in interventional studies, yet many of the included studies did not adequately address these factors. Confounders can significantly influence the outcomes of a study, potentially leading to biased results if not properly identified and controlled. Inadequate reporting of these variables can obscure the true effects of the intervention being studied, making it difficult to ascertain whether observed outcomes are genuinely attributable to the intervention or influenced by other factors [83, 87].

To enhance the reliability of research findings, future researchers should rigorously identify potential confounders during the study design phase and implement appropriate statistical methods to control for these variables in their analyses [88]. This may involve using multivariable regression techniques or stratification methods to isolate the effect of the primary intervention from other influencing factors [88]. Additionally, researchers should transparently report the methods used to adjust for confounders, including the rationale for their selection, to allow for better interpretation and replication of the study. By prioritizing the accurate identification and reporting of covariates, researchers can strengthen the validity of their conclusions and contribute to a more robust body of evidence in their field [83].

Lastly, adherence and compliance reporting in intervention and comparison groups is a critical aspect of study design that many studies overlooked. Adherence refers to the extent to which participants follow the planned intervention, while compliance encompasses the overall engagement with the study protocol. High levels of adherence and compliance are necessary to ensure that the intervention is delivered as intended and that the results accurately reflect its efficacy [89]. When these factors are not reported, it raises concerns about the generalizability of the study findings and the potential for attrition bias, where the outcomes may differ for those who did not fully engage with the intervention [89].

Future researchers should implement rigorous adherence monitoring strategies and report these metrics alongside their results. This could include the use of validated adherence scales, regular quizzes to ensure and monitor participation in educational interventions, and participant feedback mechanisms to assess compliance throughout the study [89]. Furthermore, employing per-protocol and intention-to-treat (ITT) analyses can help mitigate biases associated with non-compliance [90]. ITT analysis involves including all randomized participants in the groups to which they were originally assigned, regardless of whether they completed the intervention as intended. This approach preserves the benefits of randomization and provides a more conservative estimate of the intervention’s effect [90].

The minimal clinically/practically important difference (MCID) [91] shows amount of learning improvement after educational interventions. Therefore, there is a need to determine the amount of MCID in the future studies.

Another primary concern was the lack of appropriate pretests in several studies, which further limited the ability to ensure the comparability of study groups at baseline. Using the consolidated standards of reporting trials protocol (CONSORT) [92] recommended by the International Committee of Medical Journal Editors (ICMJE) and the World Association of Medical Editors (WAME) could improve the quality of RCTs designing and reporting [26, 93].

The major limitation in nonrandomized studies was the lack of accounting for the confounding factors in the design and analysis. The second limitation was the lack of random selection, hindering generalization and applicability to different settings. Using mixed quantitative-qualitative design that could potentially enhance the applicability of their research to specific settings. However, there were concerns regarding the clarity of the qualitative methods and the related theories and the accuracy of the quantitative techniques, specifically regarding the lack of randomization and adjustment for confounding factors.

Some of the included studies used crossover design. This study design needs a period without any intervention for washout; all the effects of the first intervention should disappear in this period. Because clinical reasoning and learning are cognitive processes, a washout period is impossible in educational intervention for promoting learning.

The lack of a comparison group in several of the studies included led to an overestimation of the interventions’ effects, which could impact the generalizability of the findings. Many of the included studies identified in this review had short-term follow-up periods, which may not capture the long-term impact of interventions on clinical reasoning skill and diagnostic accuracy. Long-term follow-up studies would provide a better understanding of the sustained effects of interventions over time.

In certain studies, each study arm received multiple concurrent interventions. As a result, it is challenging to determine how much each interventional method and component has contributed to the results that have been observed. Although employing some designs, such as factorial design, may give a clearer picture of the relative contribution of each teaching technique, this is a natural restriction in complex interventions, like the majority of interventions in medical education.

Regarding the types of assessments, using the same topics of clinical cases for training and assessment in some of the included studies may have overestimated the effects of the interventions because of the inherent benefits of practicing the topics and memorizing the diagnoses on the post intervention assessments. In addition, many included studies did not use different types of specific clinical reasoning scales, instruments, tools, or examinations for pretests, posttests, and follow-ups. Because other tests, like a multiple-choice quiz, could not measure clinical reasoning ability, this could result in overstated or underestimated predicted effects of the interventions.

Most interventions lack an adequate sample size to ascertain a precise effect size. Employing a priori sample size calculation or post hoc power calculation is necessary for acquiring precision results [27]. Future studies should specify the primary and secondary outcomes and adjust the significance level in the multiple outcome measures. Determining a practical significance level for improvement in clinical reasoning competency based on available tests is another potentially helpful endeavor in future studies of this field. Such a significant level could be used as a basis for sample size calculation and facilitate the practical application of future trials. The minimal clinically/practically important difference (MCID) [91], which is commonly used in clinical research may be a useful basis for developing such significance levels.

## Strengths and limitations

The present study identified studies with conclusive and inconclusive results that can help researchers design future studies. Considering methodological pitfalls can help researchers produce robust evidence and precise results in the future. due to the nature of scoping reviews, we conducted a broad search with diverse search terms, which was a challenge. The selection of included studies was a challenging process because of the kinds of test in pre and post-test, lack of transparency in the study design and educational strategies. We considered different types of specific clinical reasoning scales, instruments, tools, or examinations (e.g., key features examination, DTI, SCT, KFs, etc.) as the inclusion criteria in the registered protocol. Due to the low number of papers included, we were forced to modify it to at least one pretest, posttest, or follow-up test that used various sorts of clinical reasoning scales, instruments, tools, or examinations. Nevertheless, there are many studies in this filed that didn’t fulfil our inclusion criteria in study design and type of test in pretest, posttest and follow up. We didn’t search gray literatures and other databases. Despite our efforts to obtain the full text of eligible papers; we were unable to access some articles. Consequently, we excluded these articles, which may have resulted in missing a few otherwise eligible studies and their data. The present study has other limitations, including using only English-language literature and a limited number of databases. Only four key databases were searched, and consequently, we may have missed some published articles. So, it is a sample of published studies in the field of clinical reasoning teaching and learning in undergraduate medical education. We need to bear in mind that these results cannot be generalized to all of the teaching and learning clinical reasoning studies in undergraduate medical education. We did not use risk of bias assessment techniques to evaluate the included studies. The level of evidence and critical appraisal by MMAT presented in the evidence (S9 and S10 Tables) are according to dependent on information extracted from individual studies and does not take the sources of bias into account. Thus, the results of individual studies should be interpreted with caution and by considering their respective limitations according to the quality assessments.

## Conclusion

This scoping review focused on investigating various dimensions of educational intervention for teaching and learning clinical reasoning skill in undergraduate medical education. We specifically examined the use of instructional initiatives and the effect of methodological pitfalls in the studies related to this topic. Researchers can use the comprehensive features educational strategies and methodological suggestions presented herein to design their future projects. Given the limitations of current evidence, no special teaching strategy or intervention can be recommended.

## Supporting information

S1 Checklist Preferred Reporting Items for Systematic reviews and Meta-Analyses extension for Scoping Reviews (PRISMA-ScR) checklist. (PDF)

S1 Fig This is the scoping review inclusion flow diagram. (TIF)

S2 Fig This is the number of published articles in improving clinical reasoning skill in undergraduate medical students per year. (TIF)

S1 Table This is the inclusion and exclusion criteria. (PDF)

S2 Table This is the level of evidence provided by different study types. (PDF)

S3 Table This is the interpretation zone of SMD (Cohen’s d). (PDF)

S4 Table This is the identifying characteristics of included articles. (PDF)

S5 Table This is the characteristic of participants and study design. (PDF)

S6 Table This is the clinical case topic(s) in pretest, intervention, posttest, and follow up based on the included studies. (PDF)

S7 Table This is the characteristic of educational intervention for improving clinical reasoning skills based on the included studies. (PDF)

S8 Table This is the characteristics of pretest, posttest, and follow up based on the included studies. (PDF)

S9 Table This is the evidence snapshot on the effectiveness of interventions to improve clinical reasoning based on the included studies. (PDF)

S10 Table This is the critical appraisal of included studies by MMAT. (PDF)

S1 File This is the search syntax in databases. (PDF)

S2 File This is the list of excluded articles with reasons. (PDF)

S3 File This is the abbreviation file. (PDF)
