# Supplementary figures and images for: Teaching and learning clinical reasoning skill in undergraduate medical students: A scoping review
Source: PLoS One. 2024 Oct 16;19(10):e0309606. doi: 10.1371/journal.pone.0309606 (PMC11482728; doi:10.1371/journal.pone.0309606)

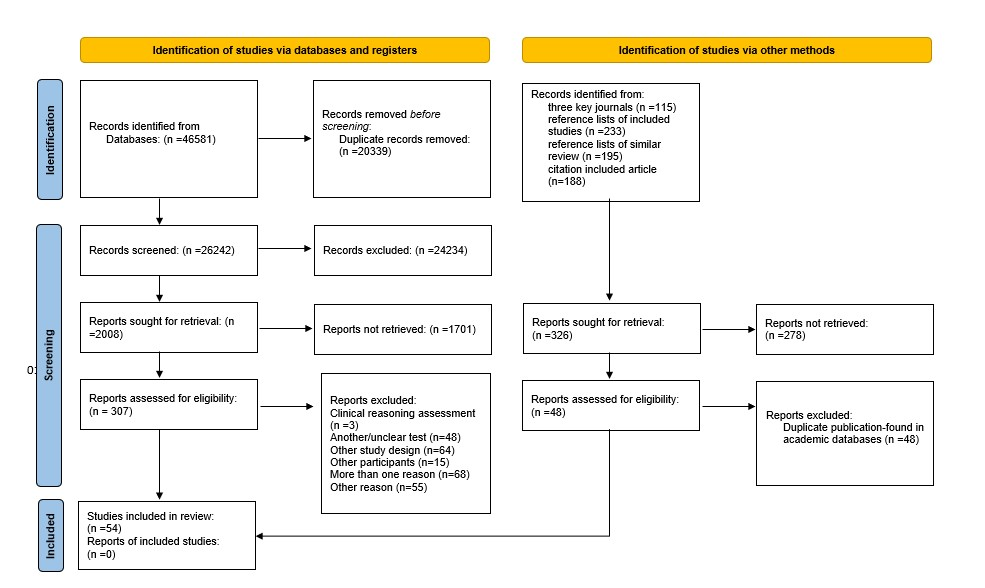

Supplement: S1 Fig — (TIF) [file pone.0309606.s002.tif]

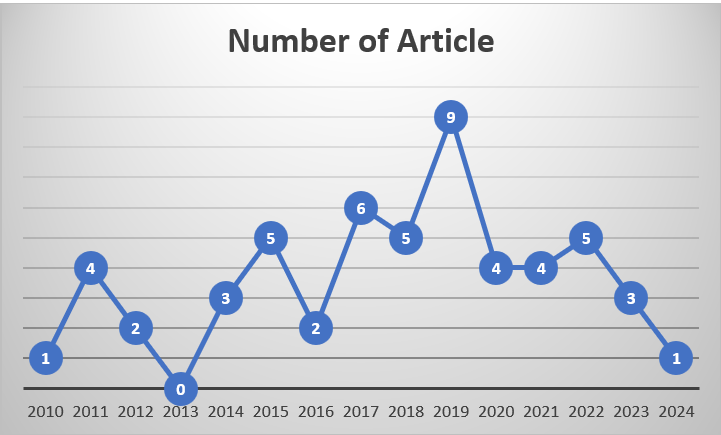

Supplement: S2 Fig — (TIF) [file pone.0309606.s003.tif]
