# Supplementary material for: Teaching and learning clinical reasoning skill in undergraduate medical students: A scoping review
Source: PLoS One. 2024 Oct 16;19(10):e0309606. doi: 10.1371/journal.pone.0309606 (PMC11482728; doi:10.1371/journal.pone.0309606)
Supplement: S1 Table — (PDF) [file pone.0309606.s004.pdf]

Criterion	Inclusion criteria	Exclusion criteria
Type of Study design	add-on trial, randomized control trial, quasi experimental, pre-experimental, cross over	observational study (e.g., cross-sectional, cohort, diagnostic accuracy, predictive model and case control articles), proceedings, case studies/case reports, descriptive studies, review articles (e.g., systematic review, meta-analysis, scoping review, etc.), editorial, letters, prospective or commentary and qualitative studies (e.g., action research, grounded theory, etc.), guideline, grey literature (i.e., books, thesis, conference proceedings/abstracts, and news/magazine articles), and protocol studies
participant	undergraduate medical students	Graduated physician and other health professions, undergraduate and postgraduate paramedical (e.g., physiotherapy, nursing, midwifery, etc.) students, postgraduate students (e.g., residency, BSc, Ph.D. of medical and paramedical sciences)
**Language**	English	Other languages
Measures of effect	Different types of specific clinical reasoning scales, instruments, tools, or exams (e.g., key features exam, diagnostic thinking inventory (DTI), CRP, SCT, etc.) at least in one of pretest, or posttest or follow up.	Multiple choice question, clinical evaluation test (e.g., OSCE, DOPS, mini-CEX, CBD, etc.), Descriptive test, oral test, image, video, self-reported, etc. in pretest, posttest, and follow up.
Date of publication	01/01/2010: 03/23/2024	Before 01/01/2010 and after 03/23/2024
Context	Eny medical or paramedical setting, and medical classroom	-
