# Supplementary material for: Teaching and learning clinical reasoning skill in undergraduate medical students: A scoping review
Source: PLoS One. 2024 Oct 16;19(10):e0309606. doi: 10.1371/journal.pone.0309606 (PMC11482728; doi:10.1371/journal.pone.0309606)
Supplement: S2 Table — (PDF) [file pone.0309606.s005.pdf]

level of evidence provided by different study types (1).

Level of evidence	Study type
I	A systematic review of level II studies
II	A randomized controlled trial
III-1	A pseudo-randomized controlled trial (i.e., alternate allocation or some other method)
III-2	A comparative study with concurrent controls:<br>Non-randomized, experimental trial<br>Cohort study<br>Case–control study<br>Interrupted time series with a control group
III-3	A comparative study without concurrent controls:<br>Historical control study<br>Two or more single-arm studies<br>Interrupted time series without a parallel control group
IV	Case series with either post-test or pre-test/post-test outcomes

1. Andrews G, Bell C, Boyce P, Gale C, Lampe L, Marwat O, et al. Royal Australian and New Zealand College of Psychiatrists clinical practice guidelines for the treatment of panic disorder, social anxiety disorder and generalised anxiety disorder. Australian & New Zealand Journal of Psychiatry. 2018;52(12):1109-72.
