# Supplementary material for: Teaching and learning clinical reasoning skill in undergraduate medical students: A scoping review
Source: PLoS One. 2024 Oct 16;19(10):e0309606. doi: 10.1371/journal.pone.0309606 (PMC11482728; doi:10.1371/journal.pone.0309606)
Supplement: S3 Table — (PDF) [file pone.0309606.s006.pdf]

Interpretation Zone of SMD (Cohen’s *d*) (1)

Zone	SMD (Cohen’s *d*)
Very small/ trivial/ inconsiderable/ ignorable	0.00-0.2
Small effect	0.2-0.49
Medium effect	0.50-0.79
Large effect	0.8-1.19
Very large effect	<1.2

1. Sawilowsky SS. New effect size rules of thumb. Journal of modern applied statistical methods. 2009;8(2):26.
