# Supplementary material for: Teaching and learning clinical reasoning skill in undergraduate medical students: A scoping review
Source: PLoS One. 2024 Oct 16;19(10):e0309606. doi: 10.1371/journal.pone.0309606 (PMC11482728; doi:10.1371/journal.pone.0309606)
Supplement: S4 Table — (PDF) [file pone.0309606.s007.pdf]

Identifying Characteristics of Included Articles

#	Study	Year	Title	First author country	Journal
1	**Aghili et al. (1)**	2012	Virtual Patient Simulation: Promotion of Clinical Reasoning Abilities of Medical Students	Iran	Knowledge Management & E-Learning: An International Journal
2	**Alavi-Moghadam et al. (2)**	2024	Teaching clinical reasoning to medical students: A brief report of case-based clinical reasoning approach	Iran	Journal of Education and Health Promotion
3	**Ali et al. (3)**	2018	Effectiveness of Various Teaching Methodologies in Developing Clinical Reasoning Skills in Undergraduate Female Medical Students	Pakistan	Khyber Medical University Journal
4	**Al Rumayyan et al. (4)**	2018	Teaching Clinical Reasoning Through Hypothetico-Deduction Is (Slightly) Better than Self-Explanation in Tutorial Groups: An Experimental Study	Saudi Arabia	Perspectives on Medical Education
5	**Al Rumayyan et al. (5)**	2021	Teaching Clinical Reasoning: An Experiment Comparing the Effects of Small-group Hypothetico-deduction Versus Self-explanation	Saudi Arabia	Health Professions Education
6	**Bonifacino et al. (6)**	2019	Implementation of a clinical reasoning curriculum for clerkship-level medical students: a pseudo-randomized and controlled study	USA	Diagnosis
7	**Bösner et al. (7)**	2015	Teaching differential diagnosis in primary care using an inverted classroom approach: student satisfaction and gain in skills and knowledge	Germany	BMC Medical Education
8	**Braun et al. (8)**	2017	Representation Scaffolds Improve Diagnostic Efficiency in Medical Students	Germany	Medical Education
9	**Brich et al. (9)**	2017	Teaching Neurology to Medical Students with a Simplified Version of Team-Based Learning	Germany	Neurology
10	**Carlson et al. (10)**	2011	The Impact of a Diagnostic Reminder System on Student Clinical Reasoning During Simulated Case Studies	USA	Simulation in Healthcare: The Journal of the Society for Simulation in Healthcare
11	**Chamberland et al. (11)**	2015	Self-Explanation in Learning Clinical Reasoning: The Added Value of Examples and Prompts	Canada	Medical Education
12	**Chamberland et al. (12)**	2011	The Influence of Medical Students' Self-Explanations on Diagnostic Performance	Canada	Medical Education
13	**Chamberland et al. (13)**	2015	Does Medical Students' Diagnostic Performance Improve by Observing Examples of Self-Explanation Provided by Peers or Experts?	Canada	Advances in Health Sciences Education
14	**Chamberland et al. (14)**	2019	Does Providing the Correct Diagnosis as Feedback after Self-Explanation Improve Medical Students' Diagnostic Performance?	Canada	BMC Medical Education
15	**Choi et al. (15)**	2020	Effects of Reflection and Immediate Feedback to Improve Clinical Reasoning of Medical Students in the Assessment of Dermatologic Conditions: A Randomized Controlled Trial	South Korea	BMC Medical Education
16	**Delavari et al. (16)**	2020	How to Develop Clinical Reasoning in Medical Students and Interns Based on Illness Script Theory: An Experimental Study	Iran	Medical journal of the Islamic Republic of Iran

**17**	**Fernandes et al. (17)**	2021	Adding Guidance to Deliberate Reflection Improves Medical Student's Diagnostic Accuracy	Brazil	Medical Education
**18**	**Fink et al. (18)**	2021	Learning to diagnose accurately through virtual patients: do reflection phases have an added benefit?	Germany	BMC Medical Education
**19**	**Gong et al. (19)**	2022	Effects of bedside team-based learning on pediatric clinical practice in Chinese medical students	China	BMC Medical Education
**20**	**Heitzmann et al. (20)**	2015	Enhancing Diagnostic Competence with Self-Explanation Prompts and Adaptable Feedback	Germany	Medical Education
**21**	**Ibiapina et al. (21)**	2014	Effects of free, cued and modelled reflection on medical students' diagnostic competence	Brazil	Medical Education
**22**	**Jost et al. (22)**	2017	Effects of Additional Team-Based Learning on Students' Clinical Reasoning Skills: A Pilot Study	Germany	BMC Research Note
**23**	**Kahl et al. (23)**	2022	A Randomized Study of Iterative Hypothesis Testing in Undergraduate Psychiatric Education	Germany	Acta Psychiatrica Scandinavica
**24**	**Kıyak et al. (24)**	2022	Can Preclinical Students Improve Their Clinical Reasoning Skills only by Taking Case-Based Online Testlets? A Randomized Controlled Study	Turkey	Innovations in Education and Teaching International
**25**	**Kiesewetter et al. (25)**	2020	Learning Clinical Reasoning: How Virtual Patient Case Format and Prior Knowledge Interact	Germany	BMC Medical Education
**26**	**Klein et al. (26)**	2019	Fostering Medical Students' Clinical Reasoning by Learning from Errors in Clinical Case Vignettes: Effects and Conditions of Additional Prompting Procedures to Foster Self-Explanations	Germany	Advances in Health Sciences Education
**27**	**Kuhn et al. (27)**	2023	Teaching medical students to apply deliberate reflection	Netherlands	Medical Teacher
**28**	**Lee et al. (28)**	2010	Using Illness Scripts to Teach Clinical Reasoning Skills to Medical Students	China	Medical Student Education
**29**	**Linsen et al. (29)**	2018	Education in Clinical Reasoning: An Experimental Study on Strategies to Foster Novice Medical Students' Engagement in Learning Activities	Netherlands	Health Professions Education
**30**	**Ludwig et al. (30)**	2018	How Can We Teach Medical Students to Choose Wisely? A Randomized Controlled Cross-Over Study of Video- Versus Text Based Case Scenarios	Germany	BMC Medicine
**31**	**Mamede et al. (31)**	2012	Reflection as a Strategy to Foster Medical Students' Acquisition of Diagnostic Competence	Netherlands	Medical Education
**32**	**Mamede et al. (32)**	2014	How Can Students' Diagnostic Competence Benefit Most from Practice with Clinical Cases? The Effects of Structured Reflection on Future Diagnosis of the Same and Novel Diseases	Netherlands	Academic Medicine
**33**	**Mamede et al. (33)**	2019	Fostering Novice Students' Diagnostic Ability: The Value of Guiding Deliberate Reflection	Netherlands	Medical Education
**34**	**Matinpour et al. (34)**	2014	Clinical Reasoning and Improvement in the Quality of Medical Education	Iran	Shieaz E-Medical Journal
**35**	**Middeke et al. (35)**	2018	Training of Clinical Reasoning with a Serious Game versus Small-Group Problem-Based Learning: A Prospective Study	Germany	PLOS ONE
**36**	**Mlika et al.(36)**	2023	Teaching clinical reasoning among undergraduate medical	Tunisia	Journal of Medical Education Development
**37**	**Moghadami et al. (37)**	2021	Teaching Clinical Reasoning to Undergraduate Medical Students by Illness Script Method: A Randomized Controlled Trial	Iran	BMC Medical Education

**38**	**Mutter et al. (38)**	2020	Case-Based Teaching: Does the Addition of High-Fidelity Simulation Make a Difference in Medical Students' Clinical Reasoning Skills?	USA	Medical Science Educator
**39**	**Oliveira et al. (39)**	2022	Teaching of Clinical Reasoning Guided by Illness Script Theory	Brazil	Arquivos Brasileiros de Cardiologia
**40**	**Ong et al. (40)**	2022	Differential Effects of Team-Based Learning on Clinical Reasoning	Singapore	The Clinical Teacher
**41**	**PEAHL et al. (41)**	2019	Impact of 4 Components of Instructional Design Video on Medical Student Medical Decision Making During the Inpatient Rounding Experience	USA	Journal of Surgical Education
**42**	**Peixoto et al. (42)**	2017	The Effect of Self-Explanation of Pathophysiological Mechanisms of Diseases on Medical Students' Diagnostic Performance	Brazil	Advances in Health Sciences Education
**43**	**Raupach et al. (43)**	2016	Test-Enhanced Learning of Clinical Reasoning: A Crossover Randomized Trial	Germany	Medical Education
**44**	**Ribeiro et al. (44)**	2019	Effects of deliberate reflection on students' engagement in learning and learning outcomes	Brazil	Medical Education
**45**	**Schubach et al. (45)**	2017	Virtual Patients in the Acquisition of Clinical Reasoning Skills: Does Presentation Mode Matter? A Quasi-Randomized Controlled Trial	Germany	BMC Medical Education
**46**	**Schuelper et al. (46)**	2019	The Impact of Medical Students' Individual Teaching Format Choice on the Learning Outcome Related to Clinical Reasoning	Germany	JMIR Medical Education
**47**	**Si et al. (47)**	2019	Developing Clinical Reasoning Skills through Argumentation with the Concept Map Method in Medical Problem-Based Learning	Korea	Interdisciplinary Journal of Problem-Based Learning
**48**	**Sobocan et al. (48)**	2017	Problem-Based Learning in Internal Medicine: Virtual Patients or Paper-Based Problems?	Slovenia	Internal Medicine Journal
**49**	**Stark et al. (49)**	2011	Case-based learning with worked examples in complex domains: Two experimental studies in undergraduate medical education	Germany	Learning and Instruction
**50**	**Stein et al. (50)**	2015	Clinical Reasoning Web-based Prototypic Module for Tutors Teaching 5th Grade Medical Students: A Pilot Randomized Study	USA	General Medicine
**51**	**Stieger et al. (51)**	2011	Diagnostic grand rounds: a new teaching concept to train diagnostic reasoning	Austria	European Journal of Radiology
**52**	**Weidenbusch et al. (52)**	2019	Can Clinical Case Discussions Foster Clinical Reasoning Skills in Undergraduate Medical Education? A Randomized Controlled Trial	Germany	BMJ Open
**53**	**Xu et al. (53)**	2023	Effectiveness of problem-based learning combined with lecture-based learning methodology in renal pathology education	China	Cogent Education
**54**	**Yousefichaijan et al. (54)**	2016	The Effect of Short-Term Workshop on Improving Clinical Reasoning Skill of Medical Students	Iran	Medical journal of the Islamic Republic of Iran

References:

1. Aghili O, Khamseh ME, Taghavinia M, Malek M, Emami Z, Baradaran HR, et al. Virtual patient simulation: Promotion of clinical reasoning abilities of medical students. Knowledge Management and E-Learning. 2012;4(4):518-27.
2. Alavi-Moghaddam M, Zeinaddini-Meymand A, Ahmadi S, Shirani A. Teaching clinical reasoning to medical students: A brief report of case-based clinical reasoning approach. Journal of education and health promotion. 2024;13(1):42.
3. Ali S, Jamil B, Ali L. EFFECTIVENESS OF VARIOUS TEACHING METHODOLOGIES IN DEVELOPING CLINICAL REASONING SKILLS IN UNDERGRADUATE FEMALE MEDICAL STUDENTS. Khyber Medical University Journal-Kmuj. 2018;10(2):71-5.
4. Al Rumayyan A, Ahmed N, Al Subait R, Al Ghamdi G, Mahzari MM, Mohamed TA, et al. Teaching clinical reasoning through hypothetico-deduction is (slightly) better than self-explanation in tutorial groups: An experimental study. Perspectives on Medical Education. 2018;7(2):93-9.
5. Al Rumayyan A, Mamede S, van Mook WNKA, Schmidt HG. Teaching Clinical Reasoning: An Experiment Comparing the Effects of Small-group Hypothetico-deduction Versus Self-explanation. Health Professions Education. 2021;7(1):12-9.
6. Bonifacino E, Follansbee WP, Farkas AH, Jeong K, McNeil MA, DiNardo DJ. Implementation of a clinical reasoning curriculum for clerkship-level medical students: a pseudo-randomized and controlled study. Diagnosis (Berlin, Germany). 2019;6(2):165-72.
7. Bösner S, Pickert J, Stibane T. Teaching differential diagnosis in primary care using an inverted classroom approach: student satisfaction and gain in skills and knowledge. BMC medical education. 2015;15:63.
8. Braun LT, Zottmann JM, Adolf C, Lottspeich C, Then C, Wirth S, et al. Representation scaffolds improve diagnostic efficiency in medical students. Medical education. 2017;51(11):1118-26.
9. Brich J, Jost M, Brustle P, Giesler M, Rijntjes M. Teaching neurology to medical students with a simplified version of team-based learning. Neurology. 2017;89(6):616-22.
10. Carlson J, Abel M, Bridges D, Tomkowiak J. The Impact of a Diagnostic Reminder System on Student Clinical Reasoning During Simulated Case Studies. Simulation in Healthcare-Journal of the Society for Simulation in Healthcare. 2011;6(1):11-7.
11. Chamberland M, Mamede S, St-Onge C, Setrakian J, Bergeron L, Schmidt H. Self-explanation in learning clinical reasoning: the added value of examples and prompts. Medical education. 2015;49(2):193-202.
12. Chamberland M, St-Onge C, Setrakian J, Lanthier L, Bergeron L, Bourget A, et al. The influence of medical students' self-explanations on diagnostic performance. Medical education. 2011;45(7):688-95.
13. Chamberland M, Mamede S, St-Onge C, Setrakian J, Schmidt HG. Does medical students’ diagnostic performance improve by observing examples of self-explanation provided by peers or experts? Advances in Health Sciences Education. 2015;20(4):981-93.
14. Chamberland M, Setrakian J, St-Onge C, Bergeron L, Mamede S, Schmidt HG. Does providing the correct diagnosis as feedback after self-explanation improve medical students diagnostic performance? BMC medical education. 2019;19(1):194.
15. Choi S, Oh S, Lee DH, Yoon HS. Effects of reflection and immediate feedback to improve clinical reasoning of medical students in the assessment of dermatologic conditions: a randomised controlled trial. BMC medical education. 2020;20(1):146.
16. Delavari S, Monajemi A, Baradaran HR, Myint PK, Yaghmaei M, Soltani Arabshahi SK. How to develop clinical reasoning in medical students and interns based on illness script theory: An experimental study. Medical journal of the Islamic Republic of Iran. 2020;34:9.
17. Fernandes RAF, Malloy-Diniz LF, de Vasconcellos MC, Camargos PAM, Ibiapina C. Adding guidance to deliberate reflection improves medical student's diagnostic accuracy. Medical education. 2021;55(10):1161-71.

18. Fink MC, Heitzmann N, Siebeck M, Fischer F, Fischer MR. Learning to diagnose accurately through virtual patients: do reflection phases have an added benefit? Bmc Medical Education. 2021;21(1).
19. Gong J, Du J, Hao J, Li L. Effects of bedside team-based learning on pediatric clinical practice in Chinese medical students. BMC medical education. 2022;22(1):264.
20. Heitzmann N, Fischer F, Kühne-Eversmann L, Fischer MR. Enhancing diagnostic competence with self-explanation prompts and adaptable feedback. Medical education. 2015;49(10):993-1003.
21. Ibiapina C, Mamede S, Moura A, Elói-Santos S, van Gog T. Effects of free, cued and modelled reflection on medical students' diagnostic competence. Medical Education. 2014;48(8):796-805.
22. Jost M, Brüstle P, Giesler M, Rijntjes M, Brich J. Effects of additional team-based learning on students' clinical reasoning skills: a pilot study. BMC research notes. 2017;10(1):282.
23. Kahl KG, Alte C, Sipos V, Kordon A, Hohagen F, Schweiger U. A randomized study of iterative hypothesis testing in undergraduate psychiatric education. Acta Psychiatr Scand. 2010;122(4):334-8.
24. Kiyak YS, Budakoglu, II, Kalaycioglu DB, Kula S, Coskun O. Can preclinical students improve their clinical reasoning skills only by taking case-based online testlets? A randomized controlled study. Innovations in Education and Teaching International. 2022.
25. Kiesewetter J, Sailer M, Jung VM, Schönberger R, Bauer E, Zottmann JM, et al. Learning clinical reasoning: how virtual patient case format and prior knowledge interact. BMC Medical Education. 2020;20(1):1-10.
26. Klein M, Otto B, Fischer MR, Stark R. Fostering medical students’ clinical reasoning by learning from errors in clinical case vignettes: effects and conditions of additional prompting procedures to foster self-explanations. Advances in Health Sciences Education. 2019;24(2):331-51.
27. Kuhn J, Mamede S, van den Berg P, Zwaan L, Elshout G, Bindels P, et al. Teaching medical students to apply deliberate reflection. Medical teacher. 2023;46(1):65-72.
28. Lee A, Joynt GM, Lee AK, Ho AM, Groves M, Vlantis AC, et al. Using illness scripts to teach clinical reasoning skills to medical students. Family medicine. 2010;42(4):255-61.
29. Linsen A, Elshout G, Pols D, Zwaan L, Mamede S. Education in clinical reasoning: an experimental study on strategies to foster novice medical students’ engagement in learning activities. Health Professions Education. 2018;4(2):86-96.
30. Ludwig S, Schuelper N, Brown J, Anders S, Raupach T. How can we teach medical students to choose wisely? A randomised controlled cross-over study of video- versus text-based case scenarios. BMC medicine. 2018;16(1):107.
31. Mamede S, van Gog T, Moura AS, de Faria RM, Peixoto JM, Rikers RM, et al. Reflection as a strategy to foster medical students' acquisition of diagnostic competence. Medical education. 2012;46(5):464-72.
32. Mamede S, van Gog T, Sampaio AM, de Faria RM, Maria JP, Schmidt HG. How can students' diagnostic competence benefit most from practice with clinical cases? The effects of structured reflection on future diagnosis of the same and novel diseases. Academic medicine : journal of the Association of American Medical Colleges. 2014;89(1):121-7.
33. Mamede S, Figueiredo-Soares T, Elói Santos SM, de Faria RMD, Schmidt HG, van Gog T. Fostering novice students' diagnostic ability: the value of guiding deliberate reflection. Medical education. 2019;53(6):628-37.
34. Matinpour M, Sedighi I, Monajemi A, Jafari F, Momtaz HE, Ali Seif Rabiei M. Clinical reasoning and improvement in the quality of medical education. Shiraz E Medical Journal. 2014;15(4):1-4.
35. Middeke A, Anders S, Schuelper M, Raupach T, Schuelper N. Training of clinical reasoning with a Serious Game versus small-group problem-based learning: A prospective study. PloS one. 2018;13(9):e0203851.

36. Mlika M, Dziri C, Jallouli M, Cheikhrouhou S, Mezni F. Teaching clinical reasoning among undergraduate medical. Journal of Medical Education Development. 2023;16(51):57-64.
37. Moghadami M, Amini M, Moghadami M, Dalal B, Charlin B. Teaching clinical reasoning to undergraduate medical students by illness script method: a randomized controlled trial. BMC medical education. 2021;21(1):87.
38. Mutter MK, Martindale JR, Shah N, Gusic ME, Wolf SJ. Case-Based Teaching: Does the Addition of High-Fidelity Simulation Make a Difference in Medical Students' Clinical Reasoning Skills? Medical science educator. 2020;30(1):307-13.
39. Oliveira JCV, Peixoto AB, Marinho GEM, Peixoto JM. Teaching of Clinical Reasoning Guided by Illness Script Theory. Arquivos Brasileiros de Cardiologia. 2022;119(5):14-21.
40. Ong KY, Ng CWQ, Tan NCK, Tan K. Differential effects of team-based learning on clinical reasoning. The clinical teacher. 2022;19(1):17-23.
41. Peahl AF, Tarr EE, Has P, Hampton BS. Impact of 4 Components of Instructional Design Video on Medical Student Medical Decision Making During the Inpatient Rounding Experience. Journal of surgical education. 2019;76(5):1286-92.
42. Peixoto JM, Mamede S, de Faria RMD, Moura AS, Santos SME, Schmidt HG. The effect of self-explanation of pathophysiological mechanisms of diseases on medical students' diagnostic performance. Advances in Health Sciences Education. 2017;22(5):1183-97.
43. Raupach T, Andresen JC, Meyer K, Strobel L, Koziolek M, Jung W, et al. Test-enhanced learning of clinical reasoning: a crossover randomised trial. Medical education. 2016;50(7):711-20.
44. Ribeiro LMC, Mamede S, de Brito EM, Moura AS, de Faria RMD, Schmidt HG. Effects of deliberate reflection on students' engagement in learning and learning outcomes. Medical education. 2019;53(4):390-7.
45. Schubach F, Goos M, Fabry G, Vach W, Boeker M. Virtual patients in the acquisition of clinical reasoning skills: does presentation mode matter? A quasi-randomized controlled trial. BMC medical education. 2017;17(1):165.
46. Schuelper N, Ludwig S, Anders S, Raupach T. The Impact of Medical Students' Individual Teaching Format Choice on the Learning Outcome Related to Clinical Reasoning. JMIR medical education. 2019;5(2):e13386.
47. Si J, Kong HH, Lee SH. Developing Clinical Reasoning Skills Through Argumentation With the Concept Map Method in Medical Problem-Based Learning. Interdisciplinary Journal of Problem-Based Learning. 2019;13(1).
48. Sobocan M, Turk N, Dinevski D, Hojs R, Balon BP. Problem-based learning in internal medicine: virtual patients or paper-based problems? Internal Medicine Journal. 2017;47(1):99-103.
49. Stark R, Kopp V, Fischer MR. Case-based learning with worked examples in complex domains: Two experimental studies in undergraduate medical education. Learning and instruction. 2011;21(1):22-33.
50. Stein GH, Tokunaga H, Ando H, Obika M, Miyoshi T, Tokuda Y, et al. Clinical Reasoning Web-based Prototypic Module for Tutors Teaching 5th Grade Medical Students : A Pilot Randomized Study. Journal of General and Family Medicine. 2015;16(1):13-25.
51. Stieger S, Praschinger A, Kletter K, Kainberger F. Diagnostic grand rounds: a new teaching concept to train diagnostic reasoning. European journal of radiology. 2011;78(3):349-52.
52. Weidenbusch M, Lenzer B, Sailer M, Strobel C, Kunisch R, Kiesewetter J, et al. Can clinical case discussions foster clinical reasoning skills in undergraduate medical education? A randomised controlled trial. BMJ open. 2019;9(9):e025973.
53. Xu G, Zhao L, Zhou M. Effectiveness of problem-based learning combined with lecture based learning methodology in renal pathology education. Cogent Education. 2023;10(1).
54. Yousefichaijan P, Jafari F, Kahbazi M, Rafiei M, Pakniyat A. The effect of short-term workshop on improving clinical reasoning skill of medical students. Medical journal of the Islamic Republic of Iran. 2016;30:396.
