# Supplementary material for: Teaching and learning clinical reasoning skill in undergraduate medical students: A scoping review
Source: PLoS One. 2024 Oct 16;19(10):e0309606. doi: 10.1371/journal.pone.0309606 (PMC11482728; doi:10.1371/journal.pone.0309606)
Supplement: S5 Table — (PDF) [file pone.0309606.s008.pdf]

characteristic of participants and study design

#	Study ID	Study Design	Setting					Participants			
			Pretest	Post Test	Follow-up	Time from intervention to follow-up	Specialty Ward Rotation	Country	Medical School Duration	Participants' Year of Training	Total number of participants
**1**	Aghili et al., 2012 (1)	RCT (add-on trial)	Y	Y	N	NA	Clinical endocrinology	Iran	7- year	6th year medical students	52
**2**	Alavi-Moghadam et al., 2024 (2)	Pre-experimental (One group before-after)	Y	N	Y	2 weeks	NR	Iran	7- year	NR	21
**3**	Ali et al., 2018(3)	RCT	Y	Y	N	NA	Pediatrics	Pakistan	4-year	final year medical students	60
**4**	Al Rumayyan et. al., 2018(4)	RCT	N	N	Y	1 Week	Internal Medicine	Saudi Arabia	6-year	2nd year medical students	88
**5**	Al Rumayyan et. al., 2021 (5)	RCT	N	N	Y	1 Week	Cardiology	Saudi Arabia	6-year	2nd year medical students	139
**6**	Bonifacino et al., 2019 (6)	Non-randomized controlled study (add-on trial)	N	Y	N	NA	Internal Medicine	USA	NR	3rd year medical students	67
**7**	Bösner et al., 2015 (7)	Mixed Methods; Pre-experimental (one-group before-after)	Y	Y	Y	1 Week	NR	Germany	NR	4th and 5th year medical students	17
**8**	Braun et al., 2017(8)	RCT	Y	Y	N	NA	Internal Medicine	Germany	NR	4th and 5th year medical students	88
**9**	Brich et al., 2017(9)	RCT (Cross-over)	N	Y	N	NA	Neurology	Germany	NR	3rd and 4th year medical students	122
**10**	Carlson et al., 2011(10)	Mixed Methods Pre-experimental (one group before-after)	Y	Y	N	NA	NR	USA	NR	4th year medical students	20

**11**	Chamberland et al., 2015(11)	RCT	Y	Y	Y	1 Week	Internal Medicine	Canada	4-year	3rd year medical students	54
**12**	Chamberland et al., 2011(12)	RCT	N	N	Y	1 Week	Internal Medicine	Canada	4-year	3rd year medical students	36
**13**	Chamberland et al., 2015(13)	RCT (add-on trial)	Y	Y	Y	1 Week	Internal Medicine	Canada	4-year	3rd year medical students	53
**14**	Chamberland et al., 2019(14)	RCT (add-on trial)	N	N	Y	1 Week	Medicine and Pediatrics	Canada	4-year	3rd year medical students	94
**15**	Choi et al., 2020(15)	RCT	Y	N	Y	2 Weeks	Dermatology	South Korea	NR	4th year medical students	87
**16**	Delavari et al., 2020(16)	Pre-experimental (One group before-after)	Y	N	N	NA	Gynecology	Iran	7- year	7th year medical students	12
										4th year medical students	15
**17**	Fernandes et al., 2021(17)	RCT	Y	Y	Y	1 Week	Pediatrics	Brazil	6- year	3rd year medical students	80
										6th year medical students	62
**18**	Fink et al., 2021 (18)	RCT (add-on trial)	Y	Y	N	NA	NR	Germany	NR	3rd to 5th year	82
**19**	Gong et al., 2022 (19)	RCT	Y	Y	Y	6 months	Pediatrics	China	NR	4th year medical students	30
**20**	Heitzmann et al., 2015(20)	RCT	Y	Y	N	NA	Cardiology	Germany	NR	NR	98
**21**	Ibiapina et al., 2014 (21)	RCT	Y	Y	Y	1 week later	Internal medicine	Brazil	6-year	5th year medical students	58
										6th year medical students	57

**22**	Jost et al., 2017(22)	Pre-Experimental (static-group comparison design)	N	Y	Y	5 days later	Neurology	Germany	NR	4th and 5th year medical students	26
**23**	Kahl et al., 2022 (23)	RCT (add-on trial)	N	Y	N	NA	Psychiatry	Germany	NR	5th year medical students	72
**24**	Kıyak et al., 2022 (24)	RCT-Factorial design	Y	Y	N	NA	General surgery, Urology	Turkey	6- year	3rd year medical students	40
**25**	Kiesewetter et al., 2020(25)	RCT	Y	Y	N	NA	NR	Germany	NR	3rd to 6th year medical students	142
**26**	Klein et al., 2019(26)	RCT	Y	Y	N	NA	NR	Germany	5-year	NR	98
**27**	Kuhn et al., 2023 (27)	Quasi-experimental	N	N	Y	5-9 days later	NR	Netherlands	NR	5th and 6th year medical students	119
**28**	Lee et al., 2010(28)	RCT	Y	N	Y	8 weeks	Family Medicine, Psychiatry	China	5-year	4th year medical students	52
**29**	Linsen et al., 2018(29)	RCT	N	N	Y	1 week	Internal Medicine	Netherlands	NR	1st year medical students	333
**30**	Ludwig et al., 2018(30)	RCT (Cross-over)	N	Y	Y	6 months	NR	Germany	NR	4th year medical students	93
**31**	Mamede et al., 2012(31)	RCT	Y	Y	Y	1 week	Internal Medicine	Brazil	6-year	4th year medical students	46
**32**	Mamede et al., 2014 (32)	RCT	Y	N	Y	1 week	Internal Medicine	Brazil	6- year	4th year medical students	110
**33**	Mamede et al., 2019 (33)	RCT	N	N	Y	2 weeks	Internal Medicine	Brazil	6-year	3rd year medical students	80
**34**	Matinpour et al., 2014(34)	Quasi-experimental (add- on trial)	Y	Y	N	NA	Pediatrics	Iran	7- year	medical interns	62
**35**	Middeke et al., 2018 (35)	Quasi-experimental	N	Y	N	NA	Internal Medicine	Germany	6-year	5th year medical students	112

**36**	Mlika et al., 2023(36)	RCT (Cross-over)	N	Y	N	NA	Internal Medicine	Tunisia	NR	3rd year medical students	72
**37**	Moghadami et al., 2021(37)	RCT (Cross-over)	Y	Y	Y	4 weeks	Internal Medicine	Iran	7-year	4th year medical students	100
**38**	Mutter et al., 2020(38)	RCT (add-on trial)	N	Y	N	NA	Internal Medicine	USA	NR	4th year medical students	96
**39**	Oliveira et al, 2022 (39)	RCT	Y	N	Y	1 week later	Internal Medicine	Brazil	NR	3rd medical students	27
**40**	Ong et al., 2022 (40)	RCT (Cross-over)	N	Y	N	NA	Neurology	Singapore	5-year	4th and 5th year medical students	179
**41**	PEAHL et al., 2019(41)	RCT (add-on trial)	Y	Y	N	NA	Ob/Gyn	USA	NR	3rd year medical students	78
**42**	Peixoto et al., 2017(42)	RCT (add-on trial)	Y	N	Y	1 week	Internal Medicine	Brazil	6-year	4thyear medical students	39
**43**	Raupach et al., 2016(43)	RCT (Cross-over)	Y	Y	Y	9 months after the first day of term.	Internal Medicine	Germany	NR	4thyear medical students	87
**44**	Ribeiro et al., 2019 (44)	RCT (add-on trial)	NR	Y	N	NA	NR	Brazil	6 years	4th	72
**45**	Schubach et al., 2017(45)	Quazi-randomized control trial	N	Y	Y	the end of the semester	General and visceral surgery	Germany	NR	4th and 5th year medical students	56
**46**	Schuelper et al., 2019(46)	Quasi-experimental	Y	Y	Y	4 months	Internal Medicine	Germany	NR	4th year medical students	75
**47**	Si et al., 2019(47)	Pre-experimental (One group pretest & posttest)	Y	Y	Y	NR	NR	Korea	NR	1st year medical students	44
										2nd year medical students	51

**48**	Sobocan et al., 2017(48)	RCT (add-on trial)	Y	Y	Y	end of the year	Internal Medicine (gastroenterology module)	Slovenia	NR	3rd year medical students	34
**49**	Stark et al. 2011 (49)	RCT-Factorial design	Y	Y	N	NA	NR	Germany	NR	3rd to 5th year	153
			Y	Y	N	NA	NR	Germany	NR	3rd to 5th year	124
**50**	Stein et al., 2015(50)	RCT	Y	Y	N	NA	Internal Medicine	Japan	NR	5th year medical student	24
**51**	Stieger et al., 2011 (51)	Pre-experimental (One group before-after)	Y	Y	N	NA	NR	Austria	NR	5th	398
**52**	Weidenbusch et al., 2019(52)	RCT	Y	Y	Y	4 weeks	Internal Medicine	Germany	NR	1st to 8th clinical semesters	90
**53**	Xu et al. 2023(53)	RCT (add-on trial)	N	N	N	NA	Nephrology	China	NR	NR	65
**54**	Yousefichaijan et al., 2016(54)	Quasi-experimental	Y	N	Y	15 days	Pediatrics	Iran	7-year	stagers	42

References:

1. Aghili O, Khamseh ME, Taghavinia M, Malek M, Emami Z, Baradaran HR, et al. Virtual patient simulation: Promotion of clinical reasoning abilities of medical students. Knowledge Management and E-Learning. 2012;4(4):518-27.
2. Alavi-Moghaddam M, Zeinaddini-Meymand A, Ahmadi S, Shirani A. Teaching clinical reasoning to medical students: A brief report of case-based clinical reasoning approach. Journal of education and health promotion. 2024;13(1):42.
3. Ali S, Jamil B, Ali L. EFFECTIVENESS OF VARIOUS TEACHING METHODOLOGIES IN DEVELOPING CLINICAL REASONING SKILLS IN UNDERGRADUATE FEMALE MEDICAL STUDENTS. Khyber Medical University Journal-Kmuj. 2018;10(2):71-5.
4. Al Rumayyan A, Ahmed N, Al Subait R, Al Ghamdi G, Mahzari MM, Mohamed TA, et al. Teaching clinical reasoning through hypothetico-deduction is (slightly) better than self-explanation in tutorial groups: An experimental study. Perspectives on Medical Education. 2018;7(2):93-9.
5. Al Rumayyan A, Mamede S, van Mook WNKA, Schmidt HG. Teaching Clinical Reasoning: An Experiment Comparing the Effects of Small-group Hypothetico-deduction Versus Self-explanation. Health Professions Education. 2021;7(1):12-9.
6. Bonifacino E, Follansbee WP, Farkas AH, Jeong K, McNeil MA, DiNardo DJ. Implementation of a clinical reasoning curriculum for clerkship-level medical students: a pseudo-randomized and controlled study. Diagnosis (Berlin, Germany). 2019;6(2):165-72.
7. Bösner S, Pickert J, Stibane T. Teaching differential diagnosis in primary care using an inverted classroom approach: student satisfaction and gain in skills and knowledge. BMC medical education. 2015;15:63.
8. Braun LT, Zottmann JM, Adolf C, Lottspeich C, Then C, Wirth S, et al. Representation scaffolds improve diagnostic efficiency in medical students. Medical education. 2017;51(11):1118-26.
9. Brich J, Jost M, Brustle P, Giesler M, Rijntjes M. Teaching neurology to medical students with a simplified version of team-based learning. Neurology. 2017;89(6):616-22.
10. Carlson J, Abel M, Bridges D, Tomkowiak J. The Impact of a Diagnostic Reminder System on Student Clinical Reasoning During Simulated Case Studies. Simulation in Healthcare-Journal of the Society for Simulation in Healthcare. 2011;6(1):11-7.
11. Chamberland M, Mamede S, St-Onge C, Setrakian J, Bergeron L, Schmidt H. Self-explanation in learning clinical reasoning: the added value of examples and prompts. Medical education. 2015;49(2):193-202.
12. Chamberland M, St-Onge C, Setrakian J, Lanthier L, Bergeron L, Bourget A, et al. The influence of medical students' self-explanations on diagnostic performance. Medical education. 2011;45(7):688-95.
13. Chamberland M, Mamede S, St-Onge C, Setrakian J, Schmidt HG. Does medical students' diagnostic performance improve by observing examples of self-explanation provided by peers or experts? Advances in Health Sciences Education. 2015;20(4):981-93.
14. Chamberland M, Setrakian J, St-Onge C, Bergeron L, Mamede S, Schmidt HG. Does providing the correct diagnosis as feedback after self-explanation improve medical students diagnostic performance? BMC medical education. 2019;19(1):194.
15. Choi S, Oh S, Lee DH, Yoon HS. Effects of reflection and immediate feedback to improve clinical reasoning of medical students in the assessment of dermatologic conditions: a randomised controlled trial. BMC medical education. 2020;20(1):146.
16. Delavari S, Monajemi A, Baradaran HR, Myint PK, Yaghmaei M, Soltani Arabshahi SK. How to develop clinical reasoning in medical students and interns based on illness script theory: An experimental study. Medical journal of the Islamic Republic of Iran. 2020;34:9.
17. Fernandes RAF, Malloy-Diniz LF, de Vasconcellos MC, Camargos PAM, Ibiapina C. Adding guidance to deliberate reflection improves medical student's diagnostic accuracy. Medical education. 2021;55(10):1161-71.

18. Fink MC, Heitzmann N, Siebeck M, Fischer F, Fischer MR. Learning to diagnose accurately through virtual patients: do reflection phases have an added benefit? Bmc Medical Education. 2021;21(1).
19. Gong J, Du J, Hao J, Li L. Effects of bedside team-based learning on pediatric clinical practice in Chinese medical students. BMC medical education. 2022;22(1):264.
20. Heitzmann N, Fischer F, Kühne-Eversmann L, Fischer MR. Enhancing diagnostic competence with self-explanation prompts and adaptable feedback. Medical education. 2015;49(10):993-1003.
21. Ibiapina C, Mamede S, Moura A, Elói-Santos S, van Gog T. Effects of free, cued and modelled reflection on medical students' diagnostic competence. Medical Education. 2014;48(8):796-805.
22. Jost M, Brüstle P, Giesler M, Rijntjes M, Brich J. Effects of additional team-based learning on students' clinical reasoning skills: a pilot study. BMC research notes. 2017;10(1):282.
23. Kahl KG, Alte C, Sipos V, Kordon A, Hohagen F, Schweiger U. A randomized study of iterative hypothesis testing in undergraduate psychiatric education. Acta Psychiatr Scand. 2010;122(4):334-8.
24. Kiyak YS, Budakoglu, II, Kalaycioglu DB, Kula S, Coskun O. Can preclinical students improve their clinical reasoning skills only by taking case-based online testlets? A randomized controlled study. Innovations in Education and Teaching International. 2022.
25. Kiesewetter J, Sailer M, Jung VM, Schönberger R, Bauer E, Zottmann JM, et al. Learning clinical reasoning: how virtual patient case format and prior knowledge interact. BMC Medical Education. 2020;20(1):1-10.
26. Klein M, Otto B, Fischer MR, Stark R. Fostering medical students' clinical reasoning by learning from errors in clinical case vignettes: effects and conditions of additional prompting procedures to foster self-explanations. Advances in Health Sciences Education. 2019;24(2):331-51.
27. Kuhn J, Mamede S, van den Berg P, Zwaan L, Elshout G, Bindels P, et al. Teaching medical students to apply deliberate reflection. Medical teacher. 2023;46(1):65-72.
28. Lee A, Joynt GM, Lee AK, Ho AM, Groves M, Vlantis AC, et al. Using illness scripts to teach clinical reasoning skills to medical students. Family medicine. 2010;42(4):255-61.
29. Linsen A, Elshout G, Pols D, Zwaan L, Mamede S. Education in clinical reasoning: an experimental study on strategies to foster novice medical students' engagement in learning activities. Health Professions Education. 2018;4(2):86-96.
30. Ludwig S, Schuelper N, Brown J, Anders S, Raupach T. How can we teach medical students to choose wisely? A randomised controlled cross-over study of video- versus text-based case scenarios. BMC medicine. 2018;16(1):107.
31. Mamede S, van Gog T, Moura AS, de Faria RM, Peixoto JM, Rikers RM, et al. Reflection as a strategy to foster medical students' acquisition of diagnostic competence. Medical education. 2012;46(5):464-72.
32. Mamede S, van Gog T, Sampaio AM, de Faria RM, Maria JP, Schmidt HG. How can students' diagnostic competence benefit most from practice with clinical cases? The effects of structured reflection on future diagnosis of the same and novel diseases. Academic medicine : journal of the Association of American Medical Colleges. 2014;89(1):121-7.
33. Mamede S, Figueiredo-Soares T, Elói Santos SM, de Faria RMD, Schmidt HG, van Gog T. Fostering novice students' diagnostic ability: the value of guiding deliberate reflection. Medical education. 2019;53(6):628-37.
34. Matinpour M, Sedighi I, Monajemi A, Jafari F, Momtaz HE, Ali Seif Rabiei M. Clinical reasoning and improvement in the quality of medical education. Shiraz E Medical Journal. 2014;15(4):1-4.
35. Middeke A, Anders S, Schuelper M, Raupach T, Schuelper N. Training of clinical reasoning with a Serious Game versus small-group problem-based learning: A prospective study. PloS one. 2018;13(9):e0203851.

36. Mlika M, Dziri C, Jallouli M, Cheikhrouhou S, Mezni F. Teaching clinical reasoning among undergraduate medical. Journal of Medical Education Development. 2023;16(51):57-64.
37. Moghadami M, Amini M, Moghadami M, Dalal B, Charlin B. Teaching clinical reasoning to undergraduate medical students by illness script method: a randomized controlled trial. BMC medical education. 2021;21(1):87.
38. Mutter MK, Martindale JR, Shah N, Gusic ME, Wolf SJ. Case-Based Teaching: Does the Addition of High-Fidelity Simulation Make a Difference in Medical Students' Clinical Reasoning Skills? Medical science educator. 2020;30(1):307-13.
39. Oliveira JCV, Peixoto AB, Marinho GEM, Peixoto JM. Teaching of Clinical Reasoning Guided by Illness Script Theory. Arquivos Brasileiros de Cardiologia. 2022;119(5):14-21.
40. Ong KY, Ng CWQ, Tan NCK, Tan K. Differential effects of team-based learning on clinical reasoning. The clinical teacher. 2022;19(1):17-23.
41. Peahl AF, Tarr EE, Has P, Hampton BS. Impact of 4 Components of Instructional Design Video on Medical Student Medical Decision Making During the Inpatient Rounding Experience. Journal of surgical education. 2019;76(5):1286-92.
42. Peixoto JM, Mamede S, de Faria RMD, Moura AS, Santos SME, Schmidt HG. The effect of self-explanation of pathophysiological mechanisms of diseases on medical students' diagnostic performance. Advances in Health Sciences Education. 2017;22(5):1183-97.
43. Raupach T, Andresen JC, Meyer K, Strobel L, Koziolek M, Jung W, et al. Test-enhanced learning of clinical reasoning: a crossover randomised trial. Medical education. 2016;50(7):711-20.
44. Ribeiro LMC, Mamede S, de Brito EM, Moura AS, de Faria RMD, Schmidt HG. Effects of deliberate reflection on students' engagement in learning and learning outcomes. Medical education. 2019;53(4):390-7.
45. Schubach F, Goos M, Fabry G, Vach W, Boeker M. Virtual patients in the acquisition of clinical reasoning skills: does presentation mode matter? A quasi-randomized controlled trial. BMC medical education. 2017;17(1):165.
46. Schuelper N, Ludwig S, Anders S, Raupach T. The Impact of Medical Students' Individual Teaching Format Choice on the Learning Outcome Related to Clinical Reasoning. JMIR medical education. 2019;5(2):e13386.
47. Si J, Kong HH, Lee SH. Developing Clinical Reasoning Skills Through Argumentation With the Concept Map Method in Medical Problem-Based Learning. Interdisciplinary Journal of Problem-Based Learning. 2019;13(1).
48. Sobocan M, Turk N, Dinevski D, Hojs R, Balon BP. Problem-based learning in internal medicine: virtual patients or paper-based problems? Internal Medicine Journal. 2017;47(1):99-103.
49. Stark R, Kopp V, Fischer MR. Case-based learning with worked examples in complex domains: Two experimental studies in undergraduate medical education. Learning and instruction. 2011;21(1):22-33.
50. Stein GH, Tokunaga H, Ando H, Obika M, Miyoshi T, Tokuda Y, et al. Clinical Reasoning Web-based Prototypic Module for Tutors Teaching 5th Grade Medical Students : A Pilot Randomized Study. Journal of General and Family Medicine. 2015;16(1):13-25.
51. Stieger S, Praschinger A, Kletter K, Kainberger F. Diagnostic grand rounds: a new teaching concept to train diagnostic reasoning. European journal of radiology. 2011;78(3):349-52.
52. Weidenbusch M, Lenzer B, Sailer M, Strobel C, Kunisch R, Kiesewetter J, et al. Can clinical case discussions foster clinical reasoning skills in undergraduate medical education? A randomised controlled trial. BMJ open. 2019;9(9):e025973.
53. Xu G, Zhao L, Zhou M. Effectiveness of problem-based learning combined with lecture based learning methodology in renal pathology education. Cogent Education. 2023;10(1).
54. Yousefichaijan P, Jafari F, Kahbazi M, Rafiei M, Pakniyat A. The effect of short-term workshop on improving clinical reasoning skill of medical students. Medical journal of the Islamic Republic of Iran. 2016;30:396.
