# Supplementary material for: Teaching and learning clinical reasoning skill in undergraduate medical students: A scoping review
Source: PLoS One. 2024 Oct 16;19(10):e0309606. doi: 10.1371/journal.pone.0309606 (PMC11482728; doi:10.1371/journal.pone.0309606)
Supplement: S6 Table — (PDF) [file pone.0309606.s009.pdf]

clinical case topic(s) in pretest, intervention, posttest, and follow up based on the included studies

#	study	Clinical case topic(s) for pretest	Clinical case topic(s) for the intervention group	Clinical case topic(s) for the comparison group 1	Clinical case topic(s) for the comparison group 2	Clinical case topic(s) for the comparison group 3	Clinical case topic(s) for posttest	Clinical case topic(s) for follow up
1	Aghili et al., 2012(1)	nodular thyroid disease and osteomalacia	nodular thyroid disease and osteomalacia	NR	NA	NA	nodular thyroid disease and osteomalacia	NA
2	Alavi-Moghaddam et al., 2024(2)	acute dyspnea, jaundice, loss of consciousness, chest pain, abdominal pain, gastrointestinal bleeding, back pain, headache, seizure, and weakness	acute dyspnea, jaundice, loss of consciousness, chest pain, abdominal pain, gastrointestinal bleeding, back pain, headache, seizure, and weakness	NA	NA	NA	NA	acute dyspnea, jaundice, loss of consciousness, chest pain, abdominal pain, gastrointestinal bleeding, back pain, headache, seizure, and weakness
3	Ali et al., 2018(3)	NR	pallor in children, hematuria in children, pyrexia of unknown origin in children, diarrhea in children	pallor in children, hematuria in children, pyrexia of unknown origin in children, diarrhea in children	pallor in children, hematuria in children, pyrexia of unknown origin in children, diarrhea in children	NA	NR	NA
4	Al Rumayyan et. Al., 2018 (4)	NA	Acute myocardial infarction with heart failure, Community-acquired pneumonia (Filler), Aortic stenosis with heart failure, Nephrotic syndrome (Filler),	Acute myocardial infarction with heart failure, Community-acquired pneumonia (Filler), Aortic stenosis with heart failure, Nephrotic syndrome (Filler),	NA	NA	NA	Stomach cancer (Filler), Chronic CAD, with decompensated heart failure by anemia, Acute pyelonephritis (Filler), Chronic mitral insufficiency with secondary

			Hypertensive cardiomyopathy, Acute viral hepatitis (Filler), Alcoholic cardiomyopathy	Hypertensive cardiomyopathy, Acute viral hepatitis (Filler), Alcoholic cardiomyopathy				heart failure, Meningoencephalitis (Filler), Hypertensive cardiomyopathy, Acute appendicitis, Viral myocarditis, Rheumatoid arthritis (Filler)
**5**	Al Rumayyan et. Al., 2021 (5)	NA	Heart failure due to cor pulmonale, Hypertension: secondary to coarctation of aorta, NSTEMI, Vasovagal syncope, non-cardiac chest pain due to costochondritis	Heart failure due to cor pulmonale, Hypertension: secondary to coarctation of aorta, NSTEMI, Vasovagal syncope, non-cardiac chest pain due to costochondritis	NA	NA	NA	STEMI, Unstable angina, Stable angina, CHF due to rheumatic mitral regurgitation, Syncope due to CHB, Pre-syncope due to VT, Resistant hypertension due to renal artery stenosis, Essential HTN with AF
**6**	Bonifacino et al., 2019 (6)	NA	NR	NR	NA	NA	NR	NA
**7**	Bösner et al., 2015 (7)	chest pain, dyspnea, abdominal pain, vertigo/dizziness	chest pain, dyspnea, abdominal pain, vertigo/dizziness	NA	NA	NA	chest pain, dyspnea, abdominal pain, vertigo/dizziness	NR
**8**	Braun et al., 2017(8)	NR	dyspnea	dyspnea	NA	NA	four further patient cases presenting with dyspnea caused by COPD, tuberculosis, pneumothorax and myocarditis.	NA
**9**	Brich et al., 2017(9)	NA	Vertigo, back pain, first epileptic seizure, acute AMS	Vertigo, back pain, first epileptic seizure, acute AMS	NA	NA	Vertigo, back pain, first epileptic seizure, acute AMS	NA
**10**	Carlson et al., 2011(10)	AAA, MS, SLE, thyrotoxicosis	AAA, MS, SLE, thyrotoxicosis	NA	NA	NA	AAA, MS, SLE, thyrotoxicosis	NA

**11**	Chamberland et al., 2015(11)	Viral hepatitis B, Chronic alcoholism, Pancreatic tumor, Hemolysis[1]	Viral hepatitis B, Chronic alcoholism, Pancreatic tumor, Hemolysis[2]	Viral hepatitis B, Chronic alcoholism, Pancreatic tumor, Hemolysis[3]	Viral hepatitis B, Chronic alcoholism, Pancreatic tumor, Hemolysis[4]	NA	Viral hepatitis B, Chronic alcoholism, Pancreatic tumor, Hemolysis[5]	Acute hepatitis[6], Cirrhosis[7], Obstructive jaundice[8], Hemolysis[9]
**12**	Chamberland et al., 2011(12)	NA	Jaundice[10], heart failure[11], NSAIDs-associated ulcer, Pneumonia, Acute pulmonary embolism, Hypovolemia	Jaundice[12], heart failure[13], NSAIDs-associated ulcer, Pneumonia, Acute pulmonary embolism, Hypovolemia	NA	NA	NA	jaundice[14], heart failure[15], Peptic ulcer, Acute prostatitis, Paraneoplastic deep vein thrombosis, Acute glomerulonephritis
**13**	Chamberland et al., 2015(13)	Viral hepatitis B, Chronic alcoholism, Pancreatic	Viral hepatitis B, Chronic alcoholism, Pancreatic tumor, Hemolysis[17]	Viral hepatitis B, Chronic alcoholism, Pancreatic tumor, Hemolysis[18]	Viral hepatitis B, Chronic alcoholism,	NA	NA	Acute hepatitis[20], Cirrhosis[21], Obstructive

[1] Autoimmune Hemolysis and idiopathic Hemolysis
[2] Autoimmune Hemolysis and idiopathic Hemolysis
[3] Autoimmune Hemolysis and idiopathic Hemolysis
[4] Autoimmune Hemolysis and idiopathic Hemolysis
[5] Autoimmune Hemolysis and idiopathic Hemolysis
[6] Viral hepatitis A & B
[7] Chronic alcoholism and Hemochromatosis
[8] Choledocholithiasis and Pancreatic tumor
[9] Auto-immune hemolysis, idiopathic hemolysis, Cold agglutinins hemolysis, Mycoplasma infection hemolysis
[10] Hepatitis B, Chronic alcoholism, Pancreatic tumor, Auto-immune Hemolysis, idiopathic Hemolysis
[11] Acute myocardial infarction, Aortic stenosis, Hypertensive cardiomyopathy, Toxic cardiomyopathy alcoholic cardiomyopathy
[12] Hepatitis B, Chronic alcoholism, Pancreatic tumor, Auto-immune Hemolysis, idiopathic Hemolysis
[13] Acute myocardial infarction, Aortic stenosis, Hypertensive cardiomyopathy, Toxic cardiomyopathy alcoholic cardiomyopathy
[14] Hepatitis A, Haemochromatosis, Choledocholithiasis, Cold agglutinins Hemolysis, Mycoplasma infection Hemolysis
[15] Chronic Coronary artery disease, anemia, Mitral insufficiency, Hypertensive cardiomyopathy, atrial fibrillation, Viral myocarditis
[17] Autoimmune Hemolysis and idiopathic Hemolysis
[18] Autoimmune Hemolysis and idiopathic Hemolysis
[20] Viral hepatitis A & B
[21] Chronic alcoholism and Hemochromatosis

		tumor, Hemolysis[16]			Pancreatic tumor, Hemolysis[19]			jaundice[22], Hemolysis[23]
**14**	Chamberland et al., 2019(14)	NA	Viral hepatitis B, Chronic alcoholism, Pancreatic tumor, Hemolysis[24]	Viral hepatitis B, Chronic alcoholism, Pancreatic tumor, Hemolysis[25]	Viral hepatitis B, Chronic alcoholism, Pancreatic tumor, Hemolysis[26]	NA	NA	Acute hepatitis[27], Cirrhosis[28], Obstructive jaundice[29], Hemolysis[30], Bleeding gastric ulcer, Acute prostatitis, Venous thrombosis, Acute tubular necrosis
**15**	Choi et al., 2020(15)	NR	HZ, Recurrent herpes simplex virus infection, Allergic contact dermatitis due to ginkgo tree Fruit, Small plaque psoriasis, large plaque psoriasis[31], Bowen's disease, Pigmented basal cell carcinoma, Intradermal nevus, Longitudinal	Alopecia areata, Actinic keratosis, Seborrheic keratosis, Wart, Vitiligo, Urticaria, Tinea, Varicella, Pityriasis versicolor, Infantile, hemangioma	NA	NA	NA	NR

[16] Autoimmune Hemolysis and idiopathic Hemolysis
[19] Autoimmune Hemolysis and idiopathic Hemolysis
[22] Choledocholithiasis and Pancreatic tumor
[23] Auto-immune hemolysis, idiopathic hemolysis, Cold agglutinins hemolysis, Mycoplasma infection hemolysis
[24] Autoimmune Hemolysis and idiopathic Hemolysis
[25] Autoimmune Hemolysis and idiopathic Hemolysis
[26] Autoimmune Hemolysis and idiopathic Hemolysis
[27] Viral hepatitis A & B
[28] Chronic alcoholism and Hemochromatosis
[29] Choledocholithiasis and Pancreatic tumor
[30] Auto-immune hemolysis, idiopathic hemolysis, Cold agglutinins hemolysis, Mycoplasma infection hemolysis
[31] confused with eczema.

			melanonychia of a child, Scabies						
**16**	Delavari et al., 2020(16)	NR	NR	NA	NA	NA	NR	NA	
**17**	Fernandes et al., 2021(17)	NA	Benign neonatal Hyperbilirubinemia , - Isoimmune-mediated hemolysis – ABO incapability, Breast milk jaundice, Pneumonia with pleural effusion, Community-acquired bacterial pneumonia, Community-acquired viral pneumonia, Acute Leukemia, newly diagnosed Immune thrombocytopenia, Aplastic anemia, Urinary tract infection, Erythema infectious, Septic arthritis	Benign neonatal Hyperbilirubinemia , - Isoimmune-mediated hemolysis – ABO incapability, Breast milk jaundice, Pneumonia with pleural effusion, Community-acquired bacterial pneumonia, Community-acquired viral pneumonia, Acute Leukemia, newly diagnosed Immune thrombocytopenia, Aplastic anemia, Urinary tract infection, Erythema infectious, Septic arthritis	Benign neonatal Hyperbilirubinemi a, - Isoimmune-mediated hemolysis – ABO incapability, Breast milk jaundice, Pneumonia with pleural effusion, Community-acquired bacterial pneumonia, Community-acquired viral pneumonia, Acute Leukemia, newly diagnosed Immune thrombocytopenia, Aplastic anemia, Urinary tract infection, Erythema infectious, Septic arthritis	NA	Benign neonatal Hyperbilirubinemia, Isoimmune-mediated hemolysis - ABO incapability, Pneumonia with pleural effusion, Community-acquired bacterial pneumonia, Acute Leukemia, newly diagnosed Immune thrombocytopenia, Nephrotic syndrome, Secondary syphilis, Acute schistosomiasis mansoni, Coarctation of the aorta, Giardiasis, Testicular torsion	Benign neonatal Hyperbilirubinemia, Isoimmune-mediated hemolysis - ABO incapability, Pneumonia with pleural effusion, Community-acquired bacterial pneumonia, Acute Leukemia, newly diagnosed Immune thrombocytopenia, Acute glomerulonephritis, Exanthem subitem, Innocent Still murmur, Rheumatic fever, Zika virus, Orchitis	
**18**	Fink et al., 2021 (18)	Hypertrophic cardiomyopath y, Pneumonia, Pulmonary embolism in case of prostate cancer	Acute posterior myocardial infarction, Pulmonary embolism due to heparin induced thrombocytopenia, Lung cancer	Acute posterior myocardial infarction, Pulmonary embolism due to heparin induced thrombocytopenia, Lung cancer	Acute posterior myocardial infarction, Pulmonary embolism due to heparin induced thrombocytopenia, Lung cancer	NA	Pulmonary embolism due to coagulation disorder, Congestive heart failure with atrial fibrillation, Hyperventilation tetany	NA	
**19**	Gong et al., 2022 (19)	NR	bronchopneumoni a, Kawasaki disease, allergic purpura, hydrothorax,	bronchopneumoni a, Kawasaki disease, allergic purpura, hydrothorax,	NA	NA	NA	NR	

			unknown fever, convulsions, bleeding, neonatal jaundice, shortness of breath	unknown fever, convulsions, bleeding, neonatal jaundice, shortness of breath				
**20**	Heitzmann et al., 2015(20)	Heart failure	Heart failure	Heart failure	Heart failure	Heart failure	Heart failure	NA
**21**	Ibiapina et al., 2014 (21)	NA	Hepatitis, Choledocholithiasis, Acute myocardial infarction, Aortic dissection, Pericarditis, Hemolytic anemia, Nephrotic syndrome, Bacterial pneumonia	Hepatitis, Choledocholithiasis, Acute myocardial infarction, Aortic dissection, Pericarditis, Hemolytic anemia, Nephrotic syndrome, Bacterial pneumonia	Hepatitis, Choledocholithiasis, Acute myocardial infarction, Aortic dissection, Pericarditis, Hemolytic anemia, Nephrotic syndrome, Bacterial pneumonia	NA	Hepatitis, Choledocholithiasis, Acute myocardial infarction, Aortic dissection, Congestive heart failure, Pyelonephritis, Hodgkin's lymphoma, Leishmaniasis	Hepatitis, Choledocholithiasis, Acute myocardial infarction, Aortic dissection, Infectious mononucleosis, Rheumatic fever, Meningitis, Gastro-esophageal reflux
**22**	Jost et al., 2017(22)	NA	vertigo, back pain, first epileptic seizure, acute altered mental status	vertigo, back pain, first epileptic seizure, acute altered mental status	NA	NA	vertigo, back pain, first epileptic seizure, acute altered mental status	multiple sclerosis, dementia, muscle diseases, neuro-oncology
**23**	Kahl et al., 2022 (23)	NA	NR	NR	NA	NA	Depression	NA
**24**	Kıyak et al., 2022 (24)	acute appendicitis, ileus, anal fissure, hemorrhoid, hernia	Acute appendicitis, ileus, anal fissure, hemorrhoid, umbilical hernia, inguinal hernia	3 UTI cases	NA	NA	acute appendicitis, ileus, anal fissure, hemorrhoid, hernia	NA
**25**	Kiesewetter et al., 2020(25)	NR	Key finding back pain (4 cases) / fever (4 cases)	Key finding back pain (4 cases) / fever (4 cases)	NA	NA	NR	NA
**26**	Klein et al., 2019(26)	arterial hypertension	arterial hypertension	arterial hypertension	arterial hypertension	NA	arterial hypertension	NA

**27**	Kuhn et al., 2023 (27)	NA	IBS[32], IBD[33], chronic pancreatitis	IBS, IBD, chronic pancreatitis, Bell's palsy, rosacea, multiple sclerosis	NA	NA	NA	pneumonia/ pulmonary embolism, myocardial infarction/ stomach ulcer, migraine/ subarachnoid hemorrhage, stomach ulcer/ cholelithiasis, Gout/ cellulite, lung carcinoma/ COPD[34]
**28**	Lee et al., 2010(28)	NA	an elderly man with a persistent cough + a middle-aged woman with an acute swollen and painful left leg	an elderly man with a persistent cough + a middle-aged woman with an acute swollen and painful left leg	NA	NA	NA	NR
**29**	Linsen et al., 2018(29)	NA	Pneumothorax	Pneumothorax	NA	NA	NA	heart failure, community-acquired pneumonia, pulmonary embolism, viral pericarditis, acute myocardial infarction, atrial fibrillation, chronic obstructive pulmonary disease exacerbation, hyperventilation, acute pyelonephritis,

[32] irritable bowel syndrome
[33] irritable bowel disease
[34] chronic obstructive pulmonary disease

								acute pancreatitis, aortic dissection, nephrotic syndrome
**30**	Ludwig et al., 2018(30)	NA	Pulmonary embolism, Arterial hypertension, Hyponatremia, Atrial fibrillation, Lupus erythematosus COPD, Pneumonia, Hyperthyroidism, Pulmonary fibrosis	Pulmonary embolism, Arterial hypertension, Hyponatremia, Atrial fibrillation, Lupus erythematosus COPD, Pneumonia, Hyperthyroidism, Pulmonary fibrosis	NA	NA	Pulmonary embolism, Arterial hypertension, Hyponatremia, Atrial fibrillation, Lupus erythematosus COPD, Pneumonia, Hyperthyroidism, Pulmonary fibrosis	Pulmonary embolism, Arterial hypertension, Hyponatremia, Atrial fibrillation, Lupus erythematosus COPD, Pneumonia, Hyperthyroidism, Pulmonary fibrosis
**31**	Mamede et al., 2012(31)	NA	Acute myocardial infarction, Acute viral pericarditis, Aortic dissection, Acute viral hepatitis, Choledocholithiasis, Hemolytic anemia	Acute myocardial infarction, Acute viral pericarditis, Aortic dissection, Acute viral hepatitis, Choledocholithiasis, Hemolytic anemia	Acute myocardial infarction, Acute viral pericarditis, Aortic dissection, Acute viral hepatitis, Choledocholithiasis, Hemolytic anemia	NA	Acute myocardial infarction, Acute viral pericarditis, Acute viral hepatitis, Choledocholithiasis, 2 Filler cases	Acute myocardial infarction, Acute viral pericarditis, Acute viral hepatitis, Choledocholithiasis, 2 Filler cases
**32**	Mamede et al., 2014 (32)	NA	Acute myocardial infarction, Choledocholithiasis, Filler cases	Acute myocardial infarction, Choledocholithiasis, Filler cases	Acute myocardial infarction, Choledocholithiasis, Filler cases	NA	NA	Acute myocardial infarction, Stable angina pectoris, Gastroesophageal reflux disease, Choledocholithiasis, Acute viral hepatitis, Hemolytic anemia, Filler cases
**33**	Mamede et al., 2019 (33)	NA	Acute viral hepatitis, Choledocholithiasis, Alcoholic cirrhosis, Acute myocardial infarction, Acute viral pericarditis, Aortic dissection, Community-acquired	Acute viral hepatitis, Choledocholithiasis, Alcoholic cirrhosis, Acute myocardial infarction, Acute viral pericarditis, Aortic dissection, Community-acquired	Acute viral hepatitis, Choledocholithiasis, Alcoholic cirrhosis, Acute myocardial infarction, Acute viral pericarditis, Aortic dissection, Community-acquired	NA	NA	Acute viral hepatitis, Choledocholithiasis, Acute myocardial infarction, Acute viral pericarditis, Pancreas carcinoma, Hemolytic anemia, Chest wall pain, Gastro-esophageal reflux, Meningoencephaliti

			pneumonia, Nephrotic syndrome, Visceral leishmaniasis	pneumonia, Nephrotic syndrome, Visceral leishmaniasis	pneumonia, Nephrotic syndrome, Visceral leishmaniasis			s, Infectious mononucleosis
**34**	Matinpour et al., 2014(34)	NR	NR	NR	NA	NA	NR	NA
**35**	Middeke et al., 2018 (35)	NA	Hodgkin lymphoma, sarcoidosis	Hodgkin lymphoma, sarcoidosis, heart failure, hyponatremi, hyperthyroidism, fever in aplasia	NA	NA	Fever in aplasia, Heart failure, Hodgkin lymphoma, Hyperthyroidisms, Hyponatremia, Sarcoidosis, NSTEMI, pancreatitis, gastrointestinal hemorrhage, asthma exacerbation	NA
**36**	Mlika et al, 2023(36)	NA	pleural tuberculosis, pulmonary tuberculosis	pleural tuberculosis, pulmonary tuberculosis	NA	NA	pleural tuberculosis, pulmonary tuberculosis	NA
**37**	Moghadami et al., 2021(37)	NR	Cirrhosis, CHF, Nephrotic syndrome	Cirrhosis, CHF, Nephrotic syndrome	NA	NA	NR	Cirrhosis, CHF, Nephrotic syndrome
**38**	Mutter et al., 2020(38)	NA	chest pain	chest pain	NA	NA	chest pain, dizziness, shortness of breath, confusion	NA
**39**	Oliveira et al., 2022 (39)	PTE, PER, HZ, MI, AD, GER, pyelonephritis, meningitis	PTE, PER, HZ	MI, AD, GER	NA	NA	No posttest	PTE, PER, HZ, MI, AD, GER, sinusitis, nephrotic syndrome
**40**	Ong et al., 2022 (40)	NA	neurological symptoms and syndromes[35], acute stroke, status epilepticus, central nervous system infections, Guillain	neurological symptoms and syndromes[36], acute stroke, status epilepticus, central nervous system infections, Guillain	NA	NA	neurological symptoms and syndromes[37], acute stroke, status epilepticus, central nervous system infections, Guillain	NA

[35] dysarthria, encephalopathy, visual symptoms, nystagmus and different patterns of weakness and numbness
[36] dysarthria, encephalopathy, visual symptoms, nystagmus and different patterns of weakness and numbness
[37] dysarthria, encephalopathy, visual symptoms, nystagmus and different patterns of weakness and numbness

			Barre syndrome and myasthenic crisis	Barre syndrome and myasthenic crisis			Barre syndrome and myasthenic crisis	
**41**	PEAHL et al., 2019(41)	febrile patient with likely endometritis	Post partum complications	Post partum complications	NA	NA	tachycardia following a postpartum hemorrhage	NA
**42**	Peixoto et al., 2017(42)	NA	Jaundice, chest pain, 2 filler cases	Jaundice, chest pain, 2 filler cases	NA	NA	No posttest	Jaundice, chest pain, 2 filler cases
**43**	Raupach et al., 2016(43)	Pulmonary embolism, Arterial hypertension, Hyponatremia, Atrial fibrillation, Lupus erythematosus , COPD, Pneumonia, Hyperthyroidis m, Pulmonary fibrosis	Coronary artery disease, Heart failure, Valvular disease, Arrhythmias, Respiratory disease, Peripheral artery disease, pulmonary embolism, myocarditis, pericarditis, Nephrotic syndrome, Electrolyte homeostasis, Renal failure, Anemia, Lymphoma, Solid tumors	Coronary artery disease, Heart failure, valvular disease, Arrhythmias, respiratory disease, Peripheral artery disease, pulmonary embolism, myocarditis, pericarditis, arrhythmias, Nephrotic syndrome, Electrolyte homeostasis, Renal failure, Anemia, Lymphoma, Solid tumors	NA	NA	Pulmonary embolism, Arterial hypertension, Hyponatremia, Atrial fibrillation, Lupus erythematosus, COPD, Pneumonia, Hyperthyroidism, Pulmonary fibrosis	Pulmonary embolism, Arterial hypertension, Hyponatremia, Atrial fibrillation, Lupus erythematosus, COPD, Pneumonia, Hyperthyroidism, Pulmonary fibrosis
**44**	Ribeiro et al., 2019 (44)	NA	jaundice	jaundice	NA	NA	Differential diagnosis of jaundice	NA
**45**	Schubach et al., 2017(45)	No pretest	Appendicitis, Diverticulitis, Cholecystitis, Crohn's disease, Ileus due to mesenteric ischemia, Ruptured abdominal aortic aneurysm, Ectopic	Appendicitis, Diverticulitis, Cholecystitis, Crohn's disease, Ileus due to mesenteric ischemia, Ruptured abdominal aortic aneurysm, Ectopic	NA	NA	gastrointestinal hemorrhage	knowledge in visceral surgery

			pregnancy, Ureteral colic	pregnancy, Ureteral colic				
**46**	Schuelper et al., 2019(46)	NR	NR	NR	NA	NA	NR	NR
**47**	Si et al., 2019(47)	NR	Barking cough, dyspnea	NA	NA	NA	NR	NR
			Jaundice, vomiting					
**48**	Sobocan et al., 2017(48)	NA	NR	NR	NA	NA	NR	NA
**49**	Stark et al. 2011 (49)	arterial hypertension	arterial hypertension	arterial hypertension	arterial hypertension	arterial hypertension	arterial hypertension	NA
		hyperthyroidism	hyperthyroidism	hyperthyroidism	hyperthyroidism	hyperthyroidism	hyperthyroidism	NA
**50**	Stein et al., 2015(50)	Streptococcus pneumoniae	fever, infection and pneumonia	fever, infection and pneumonia	NA	NA	cancerous-related lymph node obstructive pneumonia	NA
**51**	Stieger et al., 2011 (51)	NR	NR	NA	NA	NA	NR	NA
**52**	Weidenbusch et al., 2019(52)	NR	Paresthesia, fever and respiratory failure, progressive respiratory failure	Paresthesia, fever and respiratory failure, progressive respiratory failure	Paresthesia, fever and respiratory failure, progressive respiratory failure	NA	NR	NR
**53**	Xu et al., 2023(53)	NA	NR	NR	NA	NA	Idiopathic membranous nephropathy, Focal segmental glomerulosclerosis, Idiopathic membranoproliferative nephritis, nephropathy, Diabetic nephropathy, Hypertensive nephrosclerosis	NA
**54**	Yousefichaijan et al., 2016(54)	NA	NR	NR	NA	NA	NA	NR

References:

1. Aghili O, Khamseh ME, Taghavinia M, Malek M, Emami Z, Baradaran HR, et al. Virtual patient simulation: Promotion of clinical reasoning abilities of medical students. Knowledge Management and E-Learning. 2012;4(4):518-27.
2. Alavi-Moghaddam M, Zeinaddini-Meymand A, Ahmadi S, Shirani A. Teaching clinical reasoning to medical students: A brief report of case-based clinical reasoning approach. Journal of education and health promotion. 2024;13(1):42.
3. Ali S, Jamil B, Ali L. EFFECTIVENESS OF VARIOUS TEACHING METHODOLOGIES IN DEVELOPING CLINICAL REASONING SKILLS IN UNDERGRADUATE FEMALE MEDICAL STUDENTS. Khyber Medical University Journal-Kmuj. 2018;10(2):71-5.
4. Al Rumayyan A, Ahmed N, Al Subait R, Al Ghamdi G, Mahzari MM, Mohamed TA, et al. Teaching clinical reasoning through hypothetico-deduction is (slightly) better than self-explanation in tutorial groups: An experimental study. Perspectives on Medical Education. 2018;7(2):93-9.
5. Al Rumayyan A, Mamede S, van Mook WNKA, Schmidt HG. Teaching Clinical Reasoning: An Experiment Comparing the Effects of Small-group Hypothetico-deduction Versus Self-explanation. Health Professions Education. 2021;7(1):12-9.
6. Bonifacino E, Follansbee WP, Farkas AH, Jeong K, McNeil MA, DiNardo DJ. Implementation of a clinical reasoning curriculum for clerkship-level medical students: a pseudo-randomized and controlled study. Diagnosis (Berlin, Germany). 2019;6(2):165-72.
7. Bösner S, Pickert J, Stibane T. Teaching differential diagnosis in primary care using an inverted classroom approach: student satisfaction and gain in skills and knowledge. BMC medical education. 2015;15:63.
8. Braun LT, Zottmann JM, Adolf C, Lottspeich C, Then C, Wirth S, et al. Representation scaffolds improve diagnostic efficiency in medical students. Medical education. 2017;51(11):1118-26.
9. Brich J, Jost M, Brustle P, Giesler M, Rijntjes M. Teaching neurology to medical students with a simplified version of team-based learning. Neurology. 2017;89(6):616-22.
10. Carlson J, Abel M, Bridges D, Tomkowiak J. The Impact of a Diagnostic Reminder System on Student Clinical Reasoning During Simulated Case Studies. Simulation in Healthcare-Journal of the Society for Simulation in Healthcare. 2011;6(1):11-7.
11. Chamberland M, Mamede S, St-Onge C, Setrakian J, Bergeron L, Schmidt H. Self-explanation in learning clinical reasoning: the added value of examples and prompts. Medical education. 2015;49(2):193-202.
12. Chamberland M, St-Onge C, Setrakian J, Lanthier L, Bergeron L, Bourget A, et al. The influence of medical students' self-explanations on diagnostic performance. Medical education. 2011;45(7):688-95.
13. Chamberland M, Mamede S, St-Onge C, Setrakian J, Schmidt HG. Does medical students’ diagnostic performance improve by observing examples of self-explanation provided by peers or experts? Advances in Health Sciences Education. 2015;20(4):981-93.
14. Chamberland M, Setrakian J, St-Onge C, Bergeron L, Mamede S, Schmidt HG. Does providing the correct diagnosis as feedback after self-explanation improve medical students diagnostic performance? BMC medical education. 2019;19(1):194.
15. Choi S, Oh S, Lee DH, Yoon HS. Effects of reflection and immediate feedback to improve clinical reasoning of medical students in the assessment of dermatologic conditions: a randomised controlled trial. BMC medical education. 2020;20(1):146.
16. Delavari S, Monajemi A, Baradaran HR, Myint PK, Yaghmaei M, Soltani Arabshahi SK. How to develop clinical reasoning in medical students and interns based on illness script theory: An experimental study. Medical journal of the Islamic Republic of Iran. 2020;34:9.

17. Fernandes RAF, Malloy-Diniz LF, de Vasconcellos MC, Camargos PAM, Ibiapina C. Adding guidance to deliberate reflection improves medical student's diagnostic accuracy. Medical education. 2021;55(10):1161-71.
18. Fink MC, Heitzmann N, Siebeck M, Fischer F, Fischer MR. Learning to diagnose accurately through virtual patients: do reflection phases have an added benefit? Bmc Medical Education. 2021;21(1).
19. Gong J, Du J, Hao J, Li L. Effects of bedside team-based learning on pediatric clinical practice in Chinese medical students. BMC medical education. 2022;22(1):264.
20. Heitzmann N, Fischer F, Kühne-Eversmann L, Fischer MR. Enhancing diagnostic competence with self-explanation prompts and adaptable feedback. Medical education. 2015;49(10):993-1003.
21. Ibiapina C, Mamede S, Moura A, Elói-Santos S, van Gog T. Effects of free, cued and modelled reflection on medical students' diagnostic competence. Medical Education. 2014;48(8):796-805.
22. Jost M, Brüstle P, Giesler M, Rijntjes M, Brich J. Effects of additional team-based learning on students' clinical reasoning skills: a pilot study. BMC research notes. 2017;10(1):282.
23. Kahl KG, Alte C, Sipos V, Kordon A, Hohagen F, Schweiger U. A randomized study of iterative hypothesis testing in undergraduate psychiatric education. Acta Psychiatr Scand. 2010;122(4):334-8.
24. Kiyak YS, Budakoglu, II, Kalaycioglu DB, Kula S, Coskun O. Can preclinical students improve their clinical reasoning skills only by taking case-based online testlets? A randomized controlled study. Innovations in Education and Teaching International. 2022.
25. Kiesewetter J, Sailer M, Jung VM, Schönberger R, Bauer E, Zottmann JM, et al. Learning clinical reasoning: how virtual patient case format and prior knowledge interact. BMC Medical Education. 2020;20(1):1-10.
26. Klein M, Otto B, Fischer MR, Stark R. Fostering medical students' clinical reasoning by learning from errors in clinical case vignettes: effects and conditions of additional prompting procedures to foster self-explanations. Advances in Health Sciences Education. 2019;24(2):331-51.
27. Kuhn J, Mamede S, van den Berg P, Zwaan L, Elshout G, Bindels P, et al. Teaching medical students to apply deliberate reflection. Medical teacher. 2023;46(1):65-72.
28. Lee A, Joynt GM, Lee AK, Ho AM, Groves M, Vlantis AC, et al. Using illness scripts to teach clinical reasoning skills to medical students. Family medicine. 2010;42(4):255-61.
29. Linsen A, Elshout G, Pols D, Zwaan L, Mamede S. Education in clinical reasoning: an experimental study on strategies to foster novice medical students' engagement in learning activities. Health Professions Education. 2018;4(2):86-96.
30. Ludwig S, Schuelper N, Brown J, Anders S, Raupach T. How can we teach medical students to choose wisely? A randomised controlled cross-over study of video- versus text-based case scenarios. BMC medicine. 2018;16(1):107.
31. Mamede S, van Gog T, Moura AS, de Faria RM, Peixoto JM, Rikers RM, et al. Reflection as a strategy to foster medical students' acquisition of diagnostic competence. Medical education. 2012;46(5):464-72.
32. Mamede S, van Gog T, Sampaio AM, de Faria RM, Maria JP, Schmidt HG. How can students' diagnostic competence benefit most from practice with clinical cases? The effects of structured reflection on future diagnosis of the same and novel diseases. Academic medicine : journal of the Association of American Medical Colleges. 2014;89(1):121-7.
33. Mamede S, Figueiredo-Soares T, Elói Santos SM, de Faria RMD, Schmidt HG, van Gog T. Fostering novice students' diagnostic ability: the value of guiding deliberate reflection. Medical education. 2019;53(6):628-37.
34. Matinpour M, Sedighi I, Monajemi A, Jafari F, Momtaz HE, Ali Seif Rabiei M. Clinical reasoning and improvement in the quality of medical education. Shiraz E Medical Journal. 2014;15(4):1-4.

35. Middeke A, Anders S, Schuelper M, Raupach T, Schuelper N. Training of clinical reasoning with a Serious Game versus small-group problem-based learning: A prospective study. PloS one. 2018;13(9):e0203851.
36. Mlika M, Dziri C, Jallouli M, Cheikhrouhou S, Mezni F. Teaching clinical reasoning among undergraduate medical. Journal of Medical Education Development. 2023;16(51):57-64.
37. Moghadami M, Amini M, Moghadami M, Dalal B, Charlin B. Teaching clinical reasoning to undergraduate medical students by illness script method: a randomized controlled trial. BMC medical education. 2021;21(1):87.
38. Mutter MK, Martindale JR, Shah N, Gusic ME, Wolf SJ. Case-Based Teaching: Does the Addition of High-Fidelity Simulation Make a Difference in Medical Students' Clinical Reasoning Skills? Medical science educator. 2020;30(1):307-13.
39. Oliveira JCV, Peixoto AB, Marinho GEM, Peixoto JM. Teaching of Clinical Reasoning Guided by Illness Script Theory. Arquivos Brasileiros de Cardiologia. 2022;119(5):14-21.
40. Ong KY, Ng CWQ, Tan NCK, Tan K. Differential effects of team-based learning on clinical reasoning. The clinical teacher. 2022;19(1):17-23.
41. Peahl AF, Tarr EE, Has P, Hampton BS. Impact of 4 Components of Instructional Design Video on Medical Student Medical Decision Making During the Inpatient Rounding Experience. Journal of surgical education. 2019;76(5):1286-92.
42. Peixoto JM, Mamede S, de Faria RMD, Moura AS, Santos SME, Schmidt HG. The effect of self-explanation of pathophysiological mechanisms of diseases on medical students’ diagnostic performance. Advances in Health Sciences Education. 2017;22(5):1183-97.
43. Raupach T, Andresen JC, Meyer K, Strobel L, Koziolek M, Jung W, et al. Test-enhanced learning of clinical reasoning: a crossover randomised trial. Medical education. 2016;50(7):711-20.
44. Ribeiro LMC, Mamede S, de Brito EM, Moura AS, de Faria RMD, Schmidt HG. Effects of deliberate reflection on students' engagement in learning and learning outcomes. Medical education. 2019;53(4):390-7.
45. Schubach F, Goos M, Fabry G, Vach W, Boeker M. Virtual patients in the acquisition of clinical reasoning skills: does presentation mode matter? A quasi-randomized controlled trial. BMC medical education. 2017;17(1):165.
46. Schuelper N, Ludwig S, Anders S, Raupach T. The Impact of Medical Students' Individual Teaching Format Choice on the Learning Outcome Related to Clinical Reasoning. JMIR medical education. 2019;5(2):e13386.
47. Si J, Kong HH, Lee SH. Developing Clinical Reasoning Skills Through Argumentation With the Concept Map Method in Medical Problem-Based Learning. Interdisciplinary Journal of Problem-Based Learning. 2019;13(1).
48. Sobocan M, Turk N, Dinevski D, Hojs R, Balon BP. Problem-based learning in internal medicine: virtual patients or paper-based problems? Internal Medicine Journal. 2017;47(1):99-103.
49. Stark R, Kopp V, Fischer MR. Case-based learning with worked examples in complex domains: Two experimental studies in undergraduate medical education. Learning and instruction. 2011;21(1):22-33.
50. Stein GH, Tokunaga H, Ando H, Obika M, Miyoshi T, Tokuda Y, et al. Clinical Reasoning Web-based Prototypic Module for Tutors Teaching 5th Grade Medical Students : A Pilot Randomized Study. Journal of General and Family Medicine. 2015;16(1):13-25.
51. Stieger S, Praschinger A, Kletter K, Kainberger F. Diagnostic grand rounds: a new teaching concept to train diagnostic reasoning. European journal of radiology. 2011;78(3):349-52.
52. Weidenbusch M, Lenzer B, Sailer M, Strobel C, Kunisch R, Kiesewetter J, et al. Can clinical case discussions foster clinical reasoning skills in undergraduate medical education? A randomised controlled trial. BMJ open. 2019;9(9):e025973.
53. Xu G, Zhao L, Zhou M. Effectiveness of problem-based learning combined with lecture based learning methodology in renal pathology education. Cogent Education. 2023;10(1).

54. Yousefichaijan P, Jafari F, Kahbazi M, Rafiei M, Pakniyat A. The effect of short-term workshop on improving clinical reasoning skill of medical students. Medical journal of the Islamic Republic of Iran. 2016;30:396.
