# Supplementary material for: Teaching and learning clinical reasoning skill in undergraduate medical students: A scoping review
Source: PLoS One. 2024 Oct 16;19(10):e0309606. doi: 10.1371/journal.pone.0309606 (PMC11482728; doi:10.1371/journal.pone.0309606)
Supplement: S7 Table — (PDF) [file pone.0309606.s010.pdf]

characteristic of educational intervention for improving clinical reasoning skills based on the included studies

#	Study ID	Intervention Characteristics				Intervention Group		Comparison Group 1		Comparison Group 2		Comparison Group 3	
		Duration of intervention	Method of clinical case presentation	Purpose of teaching approach	Number of clinical cases	Intervention	N[1]	Comparison1	N	Comparison2	N	Comparison3	N
**1**	Aghili et al., 2012(1)	4 weeks	serial cue	Process-oriented	NR	traditional educational programs[2] + simulation + feedback	29	traditional educational programs	23	NA[3]	NA	NA	NA
**2**	Alavai-Moghaddam et al., 2024(2)	10-weekly 2-hours session	Serial cue	Process-oriented	10	Cased-based clinical reasoning	21	NA	NA	NA	NA	NA	NA
**3**	Ali et al., 2018(3)	4 weeks	NR	Process-oriented	4 topics	SNAPPS	20	traditional clinical teaching	20	one minute preceptor	20	NA	NA
**4**	Al Rumayyan et. Al., 2018 (4)	NR	serial cue **vs** whole case[4]	process-oriented	7	hypothetico-deduction	45	self-explanation	43	NA	NA	NA	NA
**5**	Al Rumayyan et. Al., 2021 (5)	NR	serial cue vs whole case[2]	process-oriented	5	hypothetico-deduction (small group)	67	self-explanation (Small group)	72	NA	NA	NA	NA
**6**	Bonifacino et al., 2019 (6)	4 weeks	NR	process-oriented	NR	six interactives online modules[5] + a case-based workshop + case based clinical reasoning discussions	34	case based clinical reasoning discussions	33	NA	NA	NA	NA

[1] Number of participants
[2] didactic lectures, case-based small group discussions, bed-side face to face teaching sessions, and interactive OPD teaching clinics.
[3] Not applicable.
[4] Intervention group: serial cue; comparison group: whole case
[5] The modules included didactic videos, simulated clinical cases, and interactive prompts for open-ended and multiple-choice questions.

**7**	Bösner et al., 2015 (7)	42 hours	NR	NR	NR	Inverted classroom	17	NA	NA	NA	NA	NA	NA
**8**	Braun et al., 2017(8)	NR	Serial cue	process-oriented	4	CASUS system cases, by representation scaffolding[6] + test enhanced learning	43	Test enhanced learning	45	NA	NA	NA	NA
**9**	Brich et al., 2017(9)	NR	NR	process and knowledge-oriented	4 topics	Seminar + TBL	56	TBL + seminar	66	NA	NA	NA	NA
**10**	Carlson et al., 2011(10)	3 days	serial cue	process-oriented	4	Isabel PRO[7] (test enhanced learning: standardized patient scenarios + mannequin-based scenario) + feedback	20	NA	NA	NA	NA	NA	NA
**11**	Chamberland et al., 2015(11)	2 hours	whole cases	knowledge-oriented	4	listened to examples of residents' SEs with prompts	19	solved word puzzles	17	listened to examples of residents' SEs without prompts	18	NA	NA
**12**	Chamberland et al., 2011(12)	2.5 hours	whole cases	knowledge-oriented	12	self-explanation	18	worked with booklets	18	NA	NA	NA	NA
**13**	Chamberland et al., 2015(13)	1.5 hours	whole cases	knowledge-oriented	4	self-explanation + listen to peer self-explanation	18	self-explanation + solve a word puzzle	16	self-explanation + listen to expert self-explanation	19	NA	NA
**14**	Chamberland et al., 2019(14)	1 hours and 15 min	whole cases	knowledge-oriented	4	immediate feedback + solved clinical cases using	31	solved clinical cases using self-explanation	32	delayed feedback + solved clinical cases	31	NA	NA

[6] interrupting case processing + writing case summaries
[7] a web-based Diagnostic reminder systems by using simulation.

						self-explanation				using self-explanation			
**15**	Choi et al., 2020(15)	2 hours	whole cases	process-oriented	10	Training with reflection and immediate feedback	27	attended outpatient clinic	34	attended outpatient clinic + lecture	26	NA	NA
**16**	Delavari et al., 2020(16)	2 hours	whole cases	process-oriented	1	Lecture + feedback + reflection+ thinking aloud + script-based questioning + test enhanced learning	12	NA	NA	NA	NA	NA	NA
		2 hours	whole cases	process-oriented	2	Lecture + feedback + reflection+ thinking aloud + script-based questioning + test enhanced learning + script-based reading	15	NA	NA	NA	NA	NA	NA
**17**	Fernandes et al., 2021(17)	NR	whole cases	process-oriented	12	Free reflection	27<br>21	Cued reflection	27<br>20	Worked example	26<br>21	NA	NA
**18**	Fink et al., 2021 (18)	4 hrs.	Serial cue	Process oriented	3	VP + concluding reflection	42	VP + accompanying reflection	39	VP without reflection	40	NA	NA
**19**	Gong et al., 2022 (19)	NR	Serial cue	process-oriented	NR	TBL + Feedback + reflection + summarizing key points and commented on students' performance by teacher	15	Traditional bedside teaching	15	NA	NA	NA	NA
**20**	Heitzmann et al., 2015(20)	NR	serial cue	process-oriented	3	self-explanation +	NR	self-explanation +	NR	Without self-explanation +	NR	Without self-explanation +	NR

						adaptable feedback + reflection on the error		Without adaptable feedback+ reflect on the error		adaptable feedback		Without adaptable feedback	
**21**	Ibiapina et al., 2014 (21)	NR	Whole cases	process-oriented	8	Modelled reflection	20<br>19	Cued reflection	19<br>16	Free reflection	19<br>22	NA	NA
**22**	Jost et al., 2017(22)	180 min	unclear	knowledge-oriented	4	seminar[8] + TBL	11	seminar	15	NA	NA	NA	NA
**23**	Kahl et al., 2022 (23)	4 Weeks	Serial cue	process-oriented	NR	Lectures + PBL + IHT + Video Film	36	Lectures + PBL + TAU	36	NA	NA	NA	NA
**24**	Kıyak et al., 2022 (24)	18 days	Serial cue	process-oriented	6	Test enhanced learning + feedback	20	Test enhanced learning + feedback	20	NA	NA	NA	NA
**25**	Kiesewetter et al., 2020(25)	140 minutes	serial cue + whole case	knowledge-oriented	8	serial cue (VP_S in CASUS system)	71	whole case (VPS in CASUS system)	71	NA	NA	NA	NA
**26**	Klein et al., 2019(26)	NR	whole case	unclear	16	The unsupported-example-condition	29	The closed prompt-condition	29	The open-prompt-condition	26	NA	NA
**27**	Kuhn et al., 2023 (27)	NR	Whole case	Process-oriented	IG[9]=3<br>CG[10]=6	Deliberate reflection + Read case + give diagnosis	58	Read case + give diagnosis	61	NA	NA	NA	NA
**28**	Lee et al., 2010(28)	3 hours	whole cases	process-oriented	5	Lecture + teaching illness script[11]	24	self-directed study	28	NA	NA	NA	NA
**29**	Linsen et al., 2018(29)	NR	Whole case	process-oriented	1	solving a clinical case using a written description of a patient encounter and	171	solving the clinical case using a video patient encounter and group discussion	162	NA	NA	NA	NA

[8] regular teaching
[9] Intervention group
[10] Comparison group
[11] test-enhanced learning + think aloud + feedback.

						individual study							
**30**	Ludwig et al., 2018(30)	10 sessions	Serial cue	process-oriented	30[12]	test enhanced learning + e-seminar + watching videos	48	test enhanced learning + e-seminar + read text cases	45	NA	NA	NA	NA
**31**	Mamede et al., 2012(31)	NR	whole case	process-oriented	6	the structured reflection condition	15	the immediate diagnosis condition	15	the differential diagnosis condition	16	NA	NA
**32**	Mamede et al., 2014 (32)	3 sessions	whole case	process-oriented	7	the structured reflection condition	39	Single diagnosis[13] + solve a word puzzle	36	Differential diagnosis + solve a word puzzle	35	NA	NA
**33**	Mamede et al., 2019 (33)	NR	whole cases	process-oriented	9	Free reflection	26	Cued reflection	26	Modelled reflection	28	NA	NA
**34**	Matinpour et al., 2014(34)	3 months	Serial cue	process-oriented	NR	workshop[14] + conventional training	32	conventional training	30	NA	NA	NA	NA
**35**	Middeke et al., 2018 (35)	6 weeks[15]	Whole cases	process-oriented	45[16]	PBL	34	Serious gam / EMERGE	78	NA	NA	NA	NA
**36**	Mlika et al., 2023(36)	2 sessions (60-90 min)	Whole cases	Process-oriented	2	CRT[17]	NR	SNAPPS	NR	NA	NA	NA	NA
**37**	Moghadami et al., 2021(37)	7 hours	whole cases	process-oriented	three diseases	think aloud + small group discussion	50	traditional lecture + small group discussion	50	NA	NA	NA	NA
**38**	Mutter et al., 2020(38)	2 hours	unclear	unclear	6	Test enhanced learning (CS-M: case scenario + manikin)	48	Test enhanced learning (CS-NM: case scenario)	48	NA	NA	NA	NA

[12] Each cases containing five key feature questions.
[13] Immediate decision
[14] clinical reasoning education (PBL) using the patient scenarios.
[15] ten 90-minute sessions
[16] PBL (5 cases); Serious gam / EMERGE (40 cases).
[17] Clinical Reasoning Technique

**39**	Oliveira et al., 2022 (39)	5 hours	Whole cases	Knowledge-oriented	6[18]	Individual study + Structured reflection + Identification and association exercises + Mind maps + Application to the resolution of clinical cases	13	Individual study + Structured reflection + Identification and association exercises + Mind maps + Application to the resolution of clinical cases	14	NA	NA	NA	NA
**40**	Ong et al., 2022 (40)	3 hours	Whole cases	Process-oriented	NR	sTBL	81	IL	98	NA	NA	NA	NA
**41**	PEAHL et al., 2019(41)	NR	NR	knowledge-oriented	NR	postpartum rounding video + usual teaching	42	usual teaching	36	NA	NA	NA	NA
**42**	Peixoto et al., 2017(42)	NR	whole cases	knowledge-oriented	8	test enhanced learning + self-explanation	20	test enhanced learning	19	NA	NA	NA	NA
**43**	Raupach et al., 2016(43)	10 sessions	whole cases	Knowledge-oriented	15	e-seminar (test enhanced learning + feedback)	45	e-seminar (studying long case narratives[19] + feedback)	42	NA	NA	NA	NA
**44**	Ribeiro et al., 2019 (44)	single session	Whole cases	Knowledge oriented	2	Deliberate Reflection + case study	36	Making differential diagnosis+ case study	36	NA	NA	NA	NA
**45**	Schubach et al., 2017(45)	2 sessions	serial cue	process-oriented	8	key feature arm[20]	30	systematic arm[21]	26	NA	NA	NA	NA
**46**	Schuelper et al., 2019(46)	3 months	Whole case	unclear	135[22]	test enhanced learning	23	test enhanced learning (text-	52	NA	NA	NA	NA

[18] 3 cases for intervention group & 3 case for comparison groups.
[19] case-based learning
[20] virtual patients + worked on multiple short cases, with the instruction being focused only on important elements.
[21] virtual patients + worked on few long cases, with the instruction being comprehensive and systematic.
[22] original KFs

						(video-based key feature questions)		based key feature questions)					
**47**	Si et al., 2019(47)	2 sessions	serial cue	process-oriented	NR	PBL + Argumentation with the Concept Map Method	44<br>51	NA	NA	NA	NA	NA	NA
**48**	Sobocan et al., 2017(48)	NR	NR	NR	NR	digital problem-based learning[23]	17	paper problem-based learning	17	NA	NA	NA	NA
**49**	Stark et al. 2011 (49)	NR	Whole cases	Process oriented	6	with errors and elaborated feedback	36	with errors and KOR[24] feedback	41	without errors and elaborated feedback	40	without errors and KOR-feedback	36
		NR	Whole cases	Process oriented	NR	with errors and elaborated feedback	30	with errors and KOR feedback	32	without errors and elaborated feedback	33	without errors and KOR-feedback	29
**50**	Stein et al., 2015(50)	4 sessions[25]	NR	NR	NR	self-study	12	Tutored syllabus	12	NA	NA	NA	NA
**51**	Stieger et al., 2011 (51)	8 sessions, (12 hrs.)	Whole cases	Knowledge oriented	23	DGR[26] + interactive discussions	398	NA	NA	NA	NA	NA	NA
**52**	Weidenbusch et al., 2019(52)	3 weeks[27]	whole case	knowledge-oriented	NR	peer-moderated live case discussions in an interactive setting	30	single-learner format utilizing an interactive multimedia platform displaying video recordings of	27	single learner format in which the students worked with the original paper cases of the NEJM	33	NA	NA

[23] virtual patients
[24] knowledge of results
[25] each session lasting 1.5 hours.
[26] Diagnostic grand rounds
[27] 5 course sessions of 90 minutes.

								the live case discussion					
**53**	Xu et al., 2023(53)	12 weeks	Whole cases	Process-oriented	NR	PBL + lecture	33	lecture	32	NA	NA	NA	NA
**54**	Yousefichaijan et al., 2016(54)	12 hours (In 2 days)	NR	process-oriented	5	the nature and process of clinical reasoning	19	no intervention	23	NA	NA	NA	NA

References:

1. Aghili O, Khamseh ME, Taghavinia M, Malek M, Emami Z, Baradaran HR, et al. Virtual patient simulation: Promotion of clinical reasoning abilities of medical students. Knowledge Management and E-Learning. 2012;4(4):518-27.
2. Alavi-Moghaddam M, Zeinaddini-Meymand A, Ahmadi S, Shirani A. Teaching clinical reasoning to medical students: A brief report of case-based clinical reasoning approach. Journal of education and health promotion. 2024;13(1):42.
3. Ali S, Jamil B, Ali L. EFFECTIVENESS OF VARIOUS TEACHING METHODOLOGIES IN DEVELOPING CLINICAL REASONING SKILLS IN UNDERGRADUATE FEMALE MEDICAL STUDENTS. Khyber Medical University Journal-Kmuj. 2018;10(2):71-5.
4. Al Rumayyan A, Ahmed N, Al Subait R, Al Ghamdi G, Mahzari MM, Mohamed TA, et al. Teaching clinical reasoning through hypothetico-deduction is (slightly) better than self-explanation in tutorial groups: An experimental study. Perspectives on Medical Education. 2018;7(2):93-9.
5. Al Rumayyan A, Mamede S, van Mook WNKA, Schmidt HG. Teaching Clinical Reasoning: An Experiment Comparing the Effects of Small-group Hypothetico-deduction Versus Self-explanation. Health Professions Education. 2021;7(1):12-9.
6. Bonifacino E, Follansbee WP, Farkas AH, Jeong K, McNeil MA, DiNardo DJ. Implementation of a clinical reasoning curriculum for clerkship-level medical students: a pseudo-randomized and controlled study. Diagnosis (Berlin, Germany). 2019;6(2):165-72.
7. Bösner S, Pickert J, Stibane T. Teaching differential diagnosis in primary care using an inverted classroom approach: student satisfaction and gain in skills and knowledge. BMC medical education. 2015;15:63.
8. Braun LT, Zottmann JM, Adolf C, Lottspeich C, Then C, Wirth S, et al. Representation scaffolds improve diagnostic efficiency in medical students. Medical education. 2017;51(11):1118-26.
9. Brich J, Jost M, Brustle P, Giesler M, Rijntjes M. Teaching neurology to medical students with a simplified version of team-based learning. Neurology. 2017;89(6):616-22.
10. Carlson J, Abel M, Bridges D, Tomkowiak J. The Impact of a Diagnostic Reminder System on Student Clinical Reasoning During Simulated Case Studies. Simulation in Healthcare-Journal of the Society for Simulation in Healthcare. 2011;6(1):11-7.
11. Chamberland M, Mamede S, St-Onge C, Setrakian J, Bergeron L, Schmidt H. Self-explanation in learning clinical reasoning: the added value of examples and prompts. Medical education. 2015;49(2):193-202.
12. Chamberland M, St-Onge C, Setrakian J, Lanthier L, Bergeron L, Bourget A, et al. The influence of medical students' self-explanations on diagnostic performance. Medical education. 2011;45(7):688-95.
13. Chamberland M, Mamede S, St-Onge C, Setrakian J, Schmidt HG. Does medical students' diagnostic performance improve by observing examples of self-explanation provided by peers or experts? Advances in Health Sciences Education. 2015;20(4):981-93.

14. Chamberland M, Setrakian J, St-Onge C, Bergeron L, Mamede S, Schmidt HG. Does providing the correct diagnosis as feedback after self-explanation improve medical students diagnostic performance? BMC medical education. 2019;19(1):194.
15. Choi S, Oh S, Lee DH, Yoon HS. Effects of reflection and immediate feedback to improve clinical reasoning of medical students in the assessment of dermatologic conditions: a randomised controlled trial. BMC medical education. 2020;20(1):146.
16. Delavari S, Monajemi A, Baradaran HR, Myint PK, Yaghmaei M, Soltani Arabshahi SK. How to develop clinical reasoning in medical students and interns based on illness script theory: An experimental study. Medical journal of the Islamic Republic of Iran. 2020;34:9.
17. Fernandes RAF, Malloy-Diniz LF, de Vasconcellos MC, Camargos PAM, Ibiapina C. Adding guidance to deliberate reflection improves medical student's diagnostic accuracy. Medical education. 2021;55(10):1161-71.
18. Fink MC, Heitzmann N, Siebeck M, Fischer F, Fischer MR. Learning to diagnose accurately through virtual patients: do reflection phases have an added benefit? Bmc Medical Education. 2021;21(1).
19. Gong J, Du J, Hao J, Li L. Effects of bedside team-based learning on pediatric clinical practice in Chinese medical students. BMC medical education. 2022;22(1):264.
20. Heitzmann N, Fischer F, Kühne-Eversmann L, Fischer MR. Enhancing diagnostic competence with self-explanation prompts and adaptable feedback. Medical education. 2015;49(10):993-1003.
21. Ibiapina C, Mamede S, Moura A, Elói-Santos S, van Gog T. Effects of free, cued and modelled reflection on medical students' diagnostic competence. Medical Education. 2014;48(8):796-805.
22. Jost M, Brüstle P, Giesler M, Rijntjes M, Brich J. Effects of additional team-based learning on students' clinical reasoning skills: a pilot study. BMC research notes. 2017;10(1):282.
23. Kahl KG, Alte C, Sipos V, Kordon A, Hohagen F, Schweiger U. A randomized study of iterative hypothesis testing in undergraduate psychiatric education. Acta Psychiatr Scand. 2010;122(4):334-8.
24. Kiyak YS, Budakoglu, II, Kalaycioglu DB, Kula S, Coskun O. Can preclinical students improve their clinical reasoning skills only by taking case-based online testlets? A randomized controlled study. Innovations in Education and Teaching International. 2022.
25. Kiesewetter J, Sailer M, Jung VM, Schönberger R, Bauer E, Zottmann JM, et al. Learning clinical reasoning: how virtual patient case format and prior knowledge interact. BMC Medical Education. 2020;20(1):1-10.
26. Klein M, Otto B, Fischer MR, Stark R. Fostering medical students’ clinical reasoning by learning from errors in clinical case vignettes: effects and conditions of additional prompting procedures to foster self-explanations. Advances in Health Sciences Education. 2019;24(2):331-51.
27. Kuhn J, Mamede S, van den Berg P, Zwaan L, Elshout G, Bindels P, et al. Teaching medical students to apply deliberate reflection. Medical teacher. 2023;46(1):65-72.
28. Lee A, Joynt GM, Lee AK, Ho AM, Groves M, Vlantis AC, et al. Using illness scripts to teach clinical reasoning skills to medical students. Family medicine. 2010;42(4):255-61.
29. Linsen A, Elshout G, Pols D, Zwaan L, Mamede S. Education in clinical reasoning: an experimental study on strategies to foster novice medical students’ engagement in learning activities. Health Professions Education. 2018;4(2):86-96.
30. Ludwig S, Schuelper N, Brown J, Anders S, Raupach T. How can we teach medical students to choose wisely? A randomised controlled cross-over study of video- versus text-based case scenarios. BMC medicine. 2018;16(1):107.
31. Mamede S, van Gog T, Moura AS, de Faria RM, Peixoto JM, Rikers RM, et al. Reflection as a strategy to foster medical students' acquisition of diagnostic competence. Medical education. 2012;46(5):464-72.

32. Mamede S, van Gog T, Sampaio AM, de Faria RM, Maria JP, Schmidt HG. How can students' diagnostic competence benefit most from practice with clinical cases? The effects of structured reflection on future diagnosis of the same and novel diseases. Academic medicine : journal of the Association of American Medical Colleges. 2014;89(1):121-7.
33. Mamede S, Figueiredo-Soares T, Elói Santos SM, de Faria RMD, Schmidt HG, van Gog T. Fostering novice students' diagnostic ability: the value of guiding deliberate reflection. Medical education. 2019;53(6):628-37.
34. Matinpour M, Sedighi I, Monajemi A, Jafari F, Momtaz HE, Ali Seif Rabiei M. Clinical reasoning and improvement in the quality of medical education. Shiraz E Medical Journal. 2014;15(4):1-4.
35. Middeke A, Anders S, Schuelper M, Raupach T, Schuelper N. Training of clinical reasoning with a Serious Game versus small-group problem-based learning: A prospective study. PloS one. 2018;13(9):e0203851.
36. Mlika M, Dziri C, Jallouli M, Cheikhrouhou S, Mezni F. Teaching clinical reasoning among undergraduate medical. Journal of Medical Education Development. 2023;16(51):57-64.
37. Moghadami M, Amini M, Moghadami M, Dalal B, Charlin B. Teaching clinical reasoning to undergraduate medical students by illness script method: a randomized controlled trial. BMC medical education. 2021;21(1):87.
38. Mutter MK, Martindale JR, Shah N, Gusic ME, Wolf SJ. Case-Based Teaching: Does the Addition of High-Fidelity Simulation Make a Difference in Medical Students' Clinical Reasoning Skills? Medical science educator. 2020;30(1):307-13.
39. Oliveira JCV, Peixoto AB, Marinho GEM, Peixoto JM. Teaching of Clinical Reasoning Guided by Illness Script Theory. Arquivos Brasileiros de Cardiologia. 2022;119(5):14-21.
40. Ong KY, Ng CWQ, Tan NCK, Tan K. Differential effects of team-based learning on clinical reasoning. The clinical teacher. 2022;19(1):17-23.
41. Peahl AF, Tarr EE, Has P, Hampton BS. Impact of 4 Components of Instructional Design Video on Medical Student Medical Decision Making During the Inpatient Rounding Experience. Journal of surgical education. 2019;76(5):1286-92.
42. Peixoto JM, Mamede S, de Faria RMD, Moura AS, Santos SME, Schmidt HG. The effect of self-explanation of pathophysiological mechanisms of diseases on medical students' diagnostic performance. Advances in Health Sciences Education. 2017;22(5):1183-97.
43. Raupach T, Andresen JC, Meyer K, Strobel L, Koziolek M, Jung W, et al. Test-enhanced learning of clinical reasoning: a crossover randomised trial. Medical education. 2016;50(7):711-20.
44. Ribeiro LMC, Mamede S, de Brito EM, Moura AS, de Faria RMD, Schmidt HG. Effects of deliberate reflection on students' engagement in learning and learning outcomes. Medical education. 2019;53(4):390-7.
45. Schubach F, Goos M, Fabry G, Vach W, Boeker M. Virtual patients in the acquisition of clinical reasoning skills: does presentation mode matter? A quasi-randomized controlled trial. BMC medical education. 2017;17(1):165.
46. Schuelper N, Ludwig S, Anders S, Raupach T. The Impact of Medical Students' Individual Teaching Format Choice on the Learning Outcome Related to Clinical Reasoning. JMIR medical education. 2019;5(2):e13386.
47. Si J, Kong HH, Lee SH. Developing Clinical Reasoning Skills Through Argumentation With the Concept Map Method in Medical Problem-Based Learning. Interdisciplinary Journal of Problem-Based Learning. 2019;13(1).
48. Sobocan M, Turk N, Dinevski D, Hojs R, Balon BP. Problem-based learning in internal medicine: virtual patients or paper-based problems? Internal Medicine Journal. 2017;47(1):99-103.
49. Stark R, Kopp V, Fischer MR. Case-based learning with worked examples in complex domains: Two experimental studies in undergraduate medical education. Learning and instruction. 2011;21(1):22-33.
50. Stein GH, Tokunaga H, Ando H, Obika M, Miyoshi T, Tokuda Y, et al. Clinical Reasoning Web-based Prototypic Module for Tutors Teaching 5th Grade Medical Students : A Pilot Randomized Study. Journal of General and Family Medicine. 2015;16(1):13-25.

51. Stieger S, Praschinger A, Kletter K, Kainberger F. Diagnostic grand rounds: a new teaching concept to train diagnostic reasoning. European journal of radiology. 2011;78(3):349-52.
52. Weidenbusch M, Lenzer B, Sailer M, Strobel C, Kunisch R, Kiesewetter J, et al. Can clinical case discussions foster clinical reasoning skills in undergraduate medical education? A randomised controlled trial. BMJ open. 2019;9(9):e025973.
53. Xu G, Zhao L, Zhou M. Effectiveness of problem-based learning combined with lecture based learning methodology in renal pathology education. Cogent Education. 2023;10(1).
54. Yousefichaijan P, Jafari F, Kahbazi M, Rafiei M, Pakniyat A. The effect of short-term workshop on improving clinical reasoning skill of medical students. Medical journal of the Islamic Republic of Iran. 2016;30:396.
