# Supplementary material for: Teaching and learning clinical reasoning skill in undergraduate medical students: A scoping review
Source: PLoS One. 2024 Oct 16;19(10):e0309606. doi: 10.1371/journal.pone.0309606 (PMC11482728; doi:10.1371/journal.pone.0309606)
Supplement: S8 Table — (PDF) [file pone.0309606.s011.pdf]

characteristics of pretest, posttest, and follow up based on the included studies

	Study ID	Pretest			Posttest			Follow-up			
		Type	Number of questions	Clinical case topic(s)	Type	Number of questions	Clinical case topic(s)	Type	Number of questions	Clinical case topic(s)	Time of follow-up test
**1**	Aghili et al., 2012(1)	clinical case scenario	10	Thyroid nodules & osteomalacia	same clinical case scenario	10	Thyroid nodules& osteomalacia	No follow-up	NA	NA	NA
**2**	Alavai-Moghaddam et al., 2024(2)	CRP, SCT, KF_s, and puzzle	NR	acute dyspnea, jaundice, loss of consciousness, chest pain, abdominal pain, gastrointestinal bleeding, back pain, headache, seizure, and weakness	NA	NA	NA	CRP, SCT, KFs, and puzzle	NR	acute dyspnea, jaundice, loss of consciousness, chest pain, abdominal pain, gastrointestinal bleeding, back pain, headache, seizure, and weakness	2 weeks after training phase
**3**	Ali et al., 2018(3)	KFP	4	NR	KFP	4	NR	No follow-up	NA	NA	NA
**4**	Al Rumayyan et. al., 2018(4)	No pretest	NA	NA	No posttest	NA	NA	clinical case scenario	8	Stomach cancer (Filler), Chronic CAD, with decompensated heart failure by anemia, Acute pyelonephritis (Filler), Chronic mitral insufficiency with secondary heart failure, Meningoencephalitis (Filler),	One week after training phase

										Hypertensive cardiomyopathy, Acute appendicitis, Viral myocarditis, Rheumatoid arthritis (Filler)	
**5**	Al Rumayyan et. Al., 2021 (5)	No pretest	NA	NA	No posttest	NA	NA	clinical case scenario	8	STEMI, Unstable angina, Stable angina, CHF due to rheumatic mitral regurgitation, Syncope due to CHB, Pre-syncope due to VT, Resistant hypertension due to renal artery stenosis, Essential HTN with AF	One week after training phase
**6**	Bonifacino et al., 2019 (6)	No pretest	NA	NA	post-curricular quiz + Interpretive summary, Differential diagnosis, Explanation of reasoning and Alternatives (IDEA) tool	20-question post-curricular quiz	NR	No follow-up	NA	NA	NA
**7**	Bösner et al., 2015 (7)	EMQ + KF_s	13 EMQ + 20 KF_S	chest pain, dyspnea, abdominal pain, vertigo/dizziness	EMQ + KF_s + Student satisfaction	NR	chest pain, dyspnea, abdominal pain, vertigo/dizziness	OSCE	NR	NR	One week later
**8**	Braun et al., 2017(8)	DTI + specific knowledge exam	DTI (41 items)	NR	MCQ + KF_s + problem-solving tasks	NR	four further patient cases presenting with dyspnea caused by COPD,	No follow-up	NA	NA	NA

							tuberculosis, pneumothorax and myocarditis.				
**9**	Brich et al., 2017(9)	No pretest	NA	NA	KF_s + MCQ	44 KF_s + 39 MCQ	Vertigo, back pain, first epileptic seizure, acute AMS	No follow-up	NA	NA	NA
**10**	Carlson et al., 2011(10)	clinical case scenario	4	AAA, MS, SLE, thyrotoxicosis	clinical case scenario	4	AAA, MS, SLE, thyrotoxicosis	No follow-up	NA	NA	NA
**11**	Chamberland et al., 2015(11)	clinical case scenario	4	Viral hepatitis B, Chronic alcoholism, Pancreatic tumor, Hemolysis[1]	clinical case scenario	4	Viral hepatitis B, Chronic alcoholism, Pancreatic tumor, Hemolysis[2]	clinical cases	12[3]	Acute hepatitis[4], Cirrhosis[5], Obstructive jaundice[6], Hemolysis[7]	One week later
**12**	Chamberland et al., 2011(12)	No pretest	NA	NA	No posttest	NA	NA	clinical case scenario	12[8]	jaundice[9], heart failure[10], Peptic ulcer, Acute prostatitis, Paraneoplastic deep vein thrombosis, Acute glomerulonephritis	One week later

[1] Autoimmune Hemolysis and idiopathic Hemolysis
[2] Autoimmune Hemolysis and idiopathic Hemolysis
[3] four near-transfer cases + four far-transfer cases + four intercalated fillers.
[4] Viral hepatitis A & B
[5] Chronic alcoholism and Hemochromatosis
[6] Choledocholithiasis and Pancreatic tumor
[7] Auto-immune hemolysis, idiopathic hemolysis, Cold agglutinins hemolysis, Mycoplasma infection hemolysis
[8] four near-transfer cases + four far-transfer cases + four intercalated fillers
[9] Hepatitis A, Haemochromatosis, Choledocholithiasis, Cold agglutinins Hemolysis, Mycoplasma infection Hemolysis
[10] Chronic Coronary artery disease, anemia, Mitral insufficiency, Hypertensive cardiomyopathy, atrial fibrillation, Viral myocarditis

**13**	Chamberland et al., 2015(13)	clinical case scenario	4	Viral hepatitis B, Chronic alcoholism, Pancreatic tumor, Hemolysis[11]	clinical case scenario	4	Viral hepatitis B, Chronic alcoholism, Pancreatic tumor, Hemolysis[12]	clinical case scenario	11[13]	Acute hepatitis[14], Cirrhosis[15], Obstructive jaundice[16], Hemolysis[17]	One week later
**14**	Chamberland et al., 2019(14)	No pretest	NA	NA	No posttest	NA	NA	clinical case scenario	12[18]	Acute hepatitis[19], Cirrhosis[20], Obstructive jaundice[21], Hemolysis[22], Bleeding gastric ulcer, Acute prostatitis, Venous thrombosis, Acute tubular necrosis	One-week later
**15**	Choi et al., 2020(15)	written clinical case scenario with photographs	20[23]	NR	No posttest	NA	NA	written clinical cases with photographs[4]	20	NR	after completing the 2-week rotation

[11] Autoimmune Hemolysis and idiopathic Hemolysis
[12] Autoimmune Hemolysis and idiopathic Hemolysis
[13] 4 training cases + 4 transfer cases + 3 filler cases
[14] Viral hepatitis A & B
[15] Chronic alcoholism and Hemochromatosis
[16] Choledocholithiasis and Pancreatic tumor
[17] Auto-immune hemolysis, idiopathic hemolysis, Cold agglutinins hemolysis, Mycoplasma infection hemolysis
[18] four near-transfer + four far-transfer + four cases on different topics
[19] Viral hepatitis A & B
[20] Chronic alcoholism and Hemochromatosis
[21] Choledocholithiasis and Pancreatic tumor
[22] Auto-immune hemolysis, idiopathic hemolysis, Cold agglutinins hemolysis, Mycoplasma infection hemolysis
[23] 10 novel cases presented in diagnostic training [training set], 10 cases with diagnoses not included in training [control set]

**16**	Delavari et al., 2020(16)	CRP	4 clinical cases (16 questions)	NR	CRP	4 new clinical cases (16 questions)	NR	No follow-up	NA	NA	NA
		KF_s	4 clinical cases	NR	KF_s	4 clinical cases					NA
**17**	Fernandes et al., 2021(17)	Self-assessment of prior knowledge + previous clinical experience	NR	NA	clinical case scenario	12[24]	Benign neonatal Hyperbilirubinemia, Isoimmune-mediated hemolysis - ABO incapability, Pneumonia with pleural effusion, Community-acquired bacterial pneumonia, Acute Leukemia, newly diagnosed Immune thrombocytopenia, Nephrotic syndrome, Secondary syphilis, Acute schistosomiasis mansoni, Coarctation of the aorta, Giardiasis, Testicular torsion	clinical cases	12[25]	Benign neonatal Hyperbilirubinemia, Isoimmune-mediated hemolysis - ABO incapability, Pneumonia with pleural effusion, Community-acquired bacterial pneumonia, Acute Leukemia, newly diagnosed Immune thrombocytopenia, Acute glomerulonephritis, Exanthem subitem, Innocent Still murmur, Rheumatic fever, Zika virus, Orchitis	7 days later
**18**	Fink et al., 2021 (18)	MCQ + KFs +VP cases	3 VP cases	Hypertrophic cardiomyopathy, Pneumonia,	new VP cases	3	Pulmonary embolism due to coagulation disorder,	NA	NA	NA	NA

[24] six cases involved the same diseases presented in all three phases and six involved various other diseases in each phase.
[25] six cases involved the same diseases presented in all three phases and six involved various other diseases in each phase.

				Pulmonary embolism in case of prostate cancer			Congestive heart failure with atrial fibrillation, Hyperventilation tetany				
**19**	Gong et al., 2022 (19)	MCQ	10	NR	students' satisfaction	NA	NA	CCS + mini_CEX	NR	NR	6 months later
**20**	Heitzmann et al., 2015(20)	knowledge-decomposition tasks + MCQ + KF_S	21 MCQ + 6 KF_S + 6 knowledge-decomposition tasks	Heart Failure	knowledge-decomposition tasks + MCQ + KF_S	21 MCQ + 18 KF_S + 18 knowledge-decomposition tasks	Heart Failure	No follow-up	NA	NA	NA
**21**	Ibiapina et al., (21)	5-point Likert scale	NR	NA	Clinical cases	8	Hepatitis, Choledocholithiasis, Acute myocardial infarction, Aortic dissection, Congestive heart failure, Pyelonephritis, Hodgkin's lymphoma, Leishmaniasis	Clinical cases	8	Hepatitis, Choledocholithiasis, Acute myocardial infarction, Aortic dissection, Infectious mononucleosis, Rheumatic fever, Meningitis, Gastro-esophageal reflux	1 week later
**22**	Jost et al., 2017(22)	No pretest	NA	NA	KF_s	13	vertigo, back pain, first epileptic seizure, acute altered mental status	MCQ	40	multiple sclerosis, dementia, muscle diseases, neuro-oncology	5 days later
**23**	Kahl et al., 2022 (23)	No pretest	NA	NA	SP + MCQ + Students Satisfaction	2 SP + 27 MCQ + 3 OQ	Depression	No follow-up	NA	NA	NA
**24**	Kıyak et al., 2022 (24)	KF_s	20	acute appendicitis, ileus, anal fissure, hemorrhoid, hernia	KF_s	20	acute appendicitis, ileus, anal fissure, hemorrhoid, hernia	No follow-up	NA	NA	NA

**25**	Kiesewetter et al., 2020(25)	MCQ + subjective content knowledge + KF_s + cognitive load	4 KF_s + 10 MCQ + 2 subjective content knowledge + 4 cognitive load	NR	KF_s + cognitive load	4 KF_s + 4 cognitive loads	NR	No follow-up	NA	NA	NA
**26**	Klein et al., 2019(26)	7-point Likert scale[26] + MC-items + KF_s + problem solving task	17 MC-items + 2 KF_s + 1 problem solving task	arterial hypertension	MC-items + KF_s+ problem solving task + 7-point Likert scale[27]	17 MC-items + 8 KF_s + 3 problem solving task	arterial hypertension	No follow-up	NA	NA	NA
**27**	Kuhn et al., 2023 (27)	NA	NA	NA	NA	NA	NA	clinical case scenario	6	pneumonia/ pulmonary embolism, myocardial infarction/ stomach ulcer, migraine/ subarachnoid hemorrhage, stomach ulcer/ cholelithiasis, Gout/ cellulite, lung carcinoma/ COPD	5-9 days later
**28**	Lee et al., 2010(28)	DTI	41 items	NA	No posttest	NA	NA	DTI + CRP	DTI (41 items) + 10 CRP	NR	8 weeks after the workshop

[26] by Jerusalem and Schwarzer
[27] by Paas and Kalyuga

**29**	Linsen et al., 2018(29)	No pretest	NA	NA	No posttest	NA	NA	written clinical cases	12[28]	heart failure, community-acquired pneumonia, pulmonary embolism, viral pericarditis, acute myocardial infarction, atrial fibrillation, chronic obstructive pulmonary disease exacerbation, hyperventilation, acute pyelonephritis, acute pancreatitis, aortic dissection, nephrotic syndrome	1 week later
**30**	Ludwig et al., 2018(30)	No pretest	NA	NA	KF_s	4[29]	Pulmonary embolism, Arterial hypertension, Hyponatremia, Atrial fibrillation, Lupus erythematosus COPD, Pneumonia, Hyperthyroidism, Pulmonary fibrosis	KF_s	4[30]	Pulmonary embolism, Arterial hypertension, Hyponatremia, Atrial fibrillation, Lupus erythematosus COPD, Pneumonia, Hyperthyroidism, Pulmonary fibrosis	6 months later
**31**	Mamede et al., 2012(31)	assessin g clinical experie nces by	NA	NA	New clinical case scenario	6	Acute myocardial infarction, Acute viral pericarditis, Acute viral hepatitis,	New clinical case scenario	6	Acute myocardial infarction, Acute viral pericarditis, Acute viral hepatitis,	1 week later

[28] 8 related diseases + 4 not related diseases
[29] new cases containing 6 or 8 key features each (consisting of these 28 items)
[30] four new cases containing 6 or 8 key features each (consisting of these 28 items)

		using 5-point scale					Choledocholithiasis, 2 Filler cases			Choledocholithiasis, 2 Filler cases	
**32**	Mamede et al., 2014 (32)	assessing clinical experiences by using 5-point scale	NA	NA	No posttest	NA	NA	clinical case scenario	9	Acute myocardial infarction, Stable angina pectoris, Gastroesophageal reflux disease, Choledocholithiasis, Acute viral hepatitis, Hemolytic anemia, Filler cases	1 week later
**33**	Mamede et al., 2019 (33)	No pretest	NA	NA	No posttest	NA	NA	New clinical case scenario	10	Acute viral hepatitis, Choledocholithiasis, Acute myocardial infarction, Acute viral pericarditis, Pancreas carcinoma, Hemolytic anemia, Chest wall pain, Gastro-esophageal reflux, Meningoencephalitis, Infectious mononucleosis	2 weeks later
**34**	Matinpour et al., 2014(34)	CRP	5[31]	NR	CRP	5	NR	No follow-up	NA	NA	NA
**35**	Middeke et al., 2018 (35)	No pretest	NA	NA	KF$_s$ + EMERGE[32]	24 KF$_s$ + 4 unknown cases in EMERGE[13]	Fever in aplasia, Heart failure, Hodgkin lymphoma, Hyperthyroidisms, Hyponatremia, Sarcoidosis, NSTEMI,	No follow-up	NA	NA	NA

[31] scenarios of common diseases
[32] serious game

							pancreatitis, gastrointestinal hemorrhage, asthma exacerbation				
**36**	Mlika et al., 2023(36)	No pretest	NA	NA	NICTALOP+ satisfaction	2	pleural tuberculosis, pulmonary tuberculosis	NA	NA	NA	NA
**37**	Moghadami et al., 2021(37)	MCQ	10	Cirrhosis, CHF, Nephrotic syndrome	MCQ	10	NR	SCT	10	Cirrhosis, CHF, Nephrotic syndrome	4 week later
**38**	Mutter et al., 2020(38)	No pretest	NA	NA	SCT	64	chest pain, dizziness, shortness of breath, confusion	No follow-up	NA	NA	NA
**39**	Oliveira et al., 2022 (39)	Clinical cases	8	PTE, PER, HZ, MI, AD, GER, pyelonephritis, meningitis	No posttest	NA	NA	Clinical cases	8	PTE, PER, HZ, MI, AD, GER, sinusitis, nephrotic syndrome	1 week later
**40**	Ong et al., 2022 (40)	No pretest	NA	NA	SCT	14 Scenarios comprising 53 questions	neurological symptoms and syndromes[33], acute stroke, status epilepticus, central nervous system infections, Guillain Barre syndrome and myasthenic crisis	No follow-up	NA	NA	NA
**41**	PEAHL et al., 2019 (41)	DTI + clinical case scenario	DTI (41 items) + 1 scenario	febrile patient with likely endometritis	DTI + clinical case scenario	DTI (41 items) + 1 scenario	tachycardia following a postpartum hemorrhage	No follow-up	NA	NA	NA
**42**	Peixoto et al.,2017(42)	self-reported level of knowledge using a	NA	NA	No posttest	NA	NA	New clinical case scenario	10	Jaundice, chest pain, 2 filler cases	1 week later

[33] dysarthria, encephalopathy, visual symptoms, nystagmus and different patterns of weakness and numbness

		5-point scale									
**43**	Raupach et al.,2016(43)	patient cases + KF_s	4 patient cases + 30 KF_s	Pulmonary embolism, Arterial hypertension, Hyponatremia, Atrial fibrillation, Lupus erythematosus , COPD, Pneumonia, Hyperthyroidis m, Pulmonary fibrosis	patient cases + KF_s	4 patient cases + 30 KF_s	Pulmonary embolism, Arterial hypertension, Hyponatremia, Atrial fibrillation, Lupus erythematosus, COPD, Pneumonia, Hyperthyroidism, Pulmonary fibrosis	patient cases + KF_s	4 patient cases + 30 KF_s	Pulmonary embolism, Arterial hypertension, Hyponatremia, Atrial fibrillation, Lupus erythematosus, COPD, Pneumonia, Hyperthyroidism, Pulmonary fibrosis	9 months after the first day of term.
**44**	Ribeiro et al., 2019 (44)	NA	NA	NA	Clinical case scenario	8	Differential diagnosis of jaundice	NA	NA	NA	NA
**45**	Schubach et al., 2017(45)	No pretest	NA	NA	SCT	24 cases[34]	gastrointestinal hemorrhage	MCQ	NR	knowledge in visceral surgery	at the end of the semester
**46**	Schuelper et al., 2019(46)	KF_s	4 cases[35]	NR	KF_s	4 cases[36]	NR	KF_s	4 cases[37]	NR	4 months later
**47**	Si et al., 2019(47)	proble m solving test + rubric	NR	NR	New problem-solving test + new rubric cases	NR	NR	New problem-solving test + new rubric cases	NR	NR	NR
**48**	Sobocan et al., 2017(48)	DTI	41 items	NA	the gastroenterolo gy knowledge exam + DTI	DTI (41 items)	NR	DTI	41 items	NA	end of the year

[34] 24 cases with 66 items nested within the cases.
[35] 4 cases histories with a total number of 28 items.
[36] 4 cases histories with a total number of 28 items.
[37] 4 cases histories with a total number of 28 items.

**49**	Stark et al. 2011 (49)	MCQ	19	arterial hypertension	KFs + problem solving task	10 KFs + 3 clinical case scenarios	arterial hypertension	NA	NA	NA	NA
		MCQ	23	hyperthyroidis m	MCQ + KFs + problem solving task	23 MCQ +10 KFs + 3 clinical case scenarios	10 KFs + 3 clinical case scenarios	NA	NA	NA	NA
**50**	Stein et al., 2015(50)	Sequent ial Questio n and Answer	8	Streptococcus pneumoniae	Slightly different sequential Question and Answer	8	cancerous-related lymph node obstructive pneumonia	No follow-up	NA	NA	NA
**51**	Stieger et al., 2011 (51)	DTI+MC Q	41 items+125 MCQ	NR	DTI	41 items	NA	NA	NA	NA	NA
**52**	Weidenbusch et al., 2019(52)	MCQ +KF_s + proble m solving tasks	29 items	NR	MCQ +KF_s + problem solving tasks + survey questionnaire [38]	29 items	NR	MCQ +KF_s + problem solving tasks	29 items	NR	4 weeks after
**53**	Xu et al., 2023(53)	No pretest	NA	NA	Clinical case scenario +satisfaction survey	NR	Idiopathic membranous nephropathy, Focal segmental glomerulosclerosis, Idiopathic membranoprolifera tive nephritis, nephropathy, Diabetic nephropathy, Hypertensive nephrosclerosis	NA	NA	NA	NA
**54**	Yousefichaijan et al., 2016(54)	DTI	41 items	NA	No posttest	NA	NA	DTI + KF +CRP	DTI (41 items)	NR	15 days after the worksho p

[38] Short questionnaire consisting of nine items that rating on a Likert scale ranging from 1 to 5.

References:

1. Aghili O, Khamseh ME, Taghavinia M, Malek M, Emami Z, Baradaran HR, et al. Virtual patient simulation: Promotion of clinical reasoning abilities of medical students. Knowledge Management and E-Learning. 2012;4(4):518-27.
2. Alavi-Moghaddam M, Zeinaddini-Meymand A, Ahmadi S, Shirani A. Teaching clinical reasoning to medical students: A brief report of case-based clinical reasoning approach. Journal of education and health promotion. 2024;13(1):42.
3. Ali S, Jamil B, Ali L. EFFECTIVENESS OF VARIOUS TEACHING METHODOLOGIES IN DEVELOPING CLINICAL REASONING SKILLS IN UNDERGRADUATE FEMALE MEDICAL STUDENTS. Khyber Medical University Journal-Kmuj. 2018;10(2):71-5.
4. Al Rumayyan A, Ahmed N, Al Subait R, Al Ghamdi G, Mahzari MM, Mohamed TA, et al. Teaching clinical reasoning through hypothetico-deduction is (slightly) better than self-explanation in tutorial groups: An experimental study. Perspectives on Medical Education. 2018;7(2):93-9.
5. Al Rumayyan A, Mamede S, van Mook WNKA, Schmidt HG. Teaching Clinical Reasoning: An Experiment Comparing the Effects of Small-group Hypothetico-deduction Versus Self-explanation. Health Professions Education. 2021;7(1):12-9.
6. Bonifacino E, Follansbee WP, Farkas AH, Jeong K, McNeil MA, DiNardo DJ. Implementation of a clinical reasoning curriculum for clerkship-level medical students: a pseudo-randomized and controlled study. Diagnosis (Berlin, Germany). 2019;6(2):165-72.
7. Bösner S, Pickert J, Stibane T. Teaching differential diagnosis in primary care using an inverted classroom approach: student satisfaction and gain in skills and knowledge. BMC medical education. 2015;15:63.
8. Braun LT, Zottmann JM, Adolf C, Lottspeich C, Then C, Wirth S, et al. Representation scaffolds improve diagnostic efficiency in medical students. Medical education. 2017;51(11):1118-26.
9. Brich J, Jost M, Brustle P, Giesler M, Rijntjes M. Teaching neurology to medical students with a simplified version of team-based learning. Neurology. 2017;89(6):616-22.
10. Carlson J, Abel M, Bridges D, Tomkowiak J. The Impact of a Diagnostic Reminder System on Student Clinical Reasoning During Simulated Case Studies. Simulation in Healthcare-Journal of the Society for Simulation in Healthcare. 2011;6(1):11-7.
11. Chamberland M, Mamede S, St-Onge C, Setrakian J, Bergeron L, Schmidt H. Self-explanation in learning clinical reasoning: the added value of examples and prompts. Medical education. 2015;49(2):193-202.
12. Chamberland M, St-Onge C, Setrakian J, Lanthier L, Bergeron L, Bourget A, et al. The influence of medical students' self-explanations on diagnostic performance. Medical education. 2011;45(7):688-95.
13. Chamberland M, Mamede S, St-Onge C, Setrakian J, Schmidt HG. Does medical students' diagnostic performance improve by observing examples of self-explanation provided by peers or experts? Advances in Health Sciences Education. 2015;20(4):981-93.
14. Chamberland M, Setrakian J, St-Onge C, Bergeron L, Mamede S, Schmidt HG. Does providing the correct diagnosis as feedback after self-explanation improve medical students diagnostic performance? BMC medical education. 2019;19(1):194.
15. Choi S, Oh S, Lee DH, Yoon HS. Effects of reflection and immediate feedback to improve clinical reasoning of medical students in the assessment of dermatologic conditions: a randomised controlled trial. BMC medical education. 2020;20(1):146.
16. Delavari S, Monajemi A, Baradaran HR, Myint PK, Yaghmaei M, Soltani Arabshahi SK. How to develop clinical reasoning in medical students and interns based on illness script theory: An experimental study. Medical journal of the Islamic Republic of Iran. 2020;34:9.
17. Fernandes RAF, Malloy-Diniz LF, de Vasconcellos MC, Camargos PAM, Ibiapina C. Adding guidance to deliberate reflection improves medical student's diagnostic accuracy. Medical education. 2021;55(10):1161-71.

18. Fink MC, Heitzmann N, Siebeck M, Fischer F, Fischer MR. Learning to diagnose accurately through virtual patients: do reflection phases have an added benefit? Bmc Medical Education. 2021;21(1).
19. Gong J, Du J, Hao J, Li L. Effects of bedside team-based learning on pediatric clinical practice in Chinese medical students. BMC medical education. 2022;22(1):264.
20. Heitzmann N, Fischer F, Kühne-Eversmann L, Fischer MR. Enhancing diagnostic competence with self-explanation prompts and adaptable feedback. Medical education. 2015;49(10):993-1003.
21. Ibiapina C, Mamede S, Moura A, Elói-Santos S, van Gog T. Effects of free, cued and modelled reflection on medical students' diagnostic competence. Medical Education. 2014;48(8):796-805.
22. Jost M, Brüstle P, Giesler M, Rijntjes M, Brich J. Effects of additional team-based learning on students' clinical reasoning skills: a pilot study. BMC research notes. 2017;10(1):282.
23. Kahl KG, Alte C, Sipos V, Kordon A, Hohagen F, Schweiger U. A randomized study of iterative hypothesis testing in undergraduate psychiatric education. Acta Psychiatr Scand. 2010;122(4):334-8.
24. Kiyak YS, Budakoglu, II, Kalaycioglu DB, Kula S, Coskun O. Can preclinical students improve their clinical reasoning skills only by taking case-based online testlets? A randomized controlled study. Innovations in Education and Teaching International. 2022.
25. Kiesewetter J, Sailer M, Jung VM, Schönberger R, Bauer E, Zottmann JM, et al. Learning clinical reasoning: how virtual patient case format and prior knowledge interact. BMC Medical Education. 2020;20(1):1-10.
26. Klein M, Otto B, Fischer MR, Stark R. Fostering medical students’ clinical reasoning by learning from errors in clinical case vignettes: effects and conditions of additional prompting procedures to foster self-explanations. Advances in Health Sciences Education. 2019;24(2):331-51.
27. Kuhn J, Mamede S, van den Berg P, Zwaan L, Elshout G, Bindels P, et al. Teaching medical students to apply deliberate reflection. Medical teacher. 2023;46(1):65-72.
28. Lee A, Joynt GM, Lee AK, Ho AM, Groves M, Vlantis AC, et al. Using illness scripts to teach clinical reasoning skills to medical students. Family medicine. 2010;42(4):255-61.
29. Linsen A, Elshout G, Pols D, Zwaan L, Mamede S. Education in clinical reasoning: an experimental study on strategies to foster novice medical students’ engagement in learning activities. Health Professions Education. 2018;4(2):86-96.
30. Ludwig S, Schuelper N, Brown J, Anders S, Raupach T. How can we teach medical students to choose wisely? A randomised controlled cross-over study of video- versus text-based case scenarios. BMC medicine. 2018;16(1):107.
31. Mamede S, van Gog T, Moura AS, de Faria RM, Peixoto JM, Rikers RM, et al. Reflection as a strategy to foster medical students' acquisition of diagnostic competence. Medical education. 2012;46(5):464-72.
32. Mamede S, van Gog T, Sampaio AM, de Faria RM, Maria JP, Schmidt HG. How can students' diagnostic competence benefit most from practice with clinical cases? The effects of structured reflection on future diagnosis of the same and novel diseases. Academic medicine : journal of the Association of American Medical Colleges. 2014;89(1):121-7.
33. Mamede S, Figueiredo-Soares T, Elói Santos SM, de Faria RMD, Schmidt HG, van Gog T. Fostering novice students' diagnostic ability: the value of guiding deliberate reflection. Medical education. 2019;53(6):628-37.
34. Matinpour M, Sedighi I, Monajemi A, Jafari F, Momtaz HE, Ali Seif Rabiei M. Clinical reasoning and improvement in the quality of medical education. Shiraz E Medical Journal. 2014;15(4):1-4.
35. Middeke A, Anders S, Schuelper M, Raupach T, Schuelper N. Training of clinical reasoning with a Serious Game versus small-group problem-based learning: A prospective study. PloS one. 2018;13(9):e0203851.

36. Mlika M, Dziri C, Jallouli M, Cheikhrouhou S, Mezni F. Teaching clinical reasoning among undergraduate medical. Journal of Medical Education Development. 2023;16(51):57-64.
37. Moghadami M, Amini M, Moghadami M, Dalal B, Charlin B. Teaching clinical reasoning to undergraduate medical students by illness script method: a randomized controlled trial. BMC medical education. 2021;21(1):87.
38. Mutter MK, Martindale JR, Shah N, Gusic ME, Wolf SJ. Case-Based Teaching: Does the Addition of High-Fidelity Simulation Make a Difference in Medical Students' Clinical Reasoning Skills? Medical science educator. 2020;30(1):307-13.
39. Oliveira JCV, Peixoto AB, Marinho GEM, Peixoto JM. Teaching of Clinical Reasoning Guided by Illness Script Theory. Arquivos Brasileiros de Cardiologia. 2022;119(5):14-21.
40. Ong KY, Ng CWQ, Tan NCK, Tan K. Differential effects of team-based learning on clinical reasoning. The clinical teacher. 2022;19(1):17-23.
41. Peahl AF, Tarr EE, Has P, Hampton BS. Impact of 4 Components of Instructional Design Video on Medical Student Medical Decision Making During the Inpatient Rounding Experience. Journal of surgical education. 2019;76(5):1286-92.
42. Peixoto JM, Mamede S, de Faria RMD, Moura AS, Santos SME, Schmidt HG. The effect of self-explanation of pathophysiological mechanisms of diseases on medical students’ diagnostic performance. Advances in Health Sciences Education. 2017;22(5):1183-97.
43. Raupach T, Andresen JC, Meyer K, Strobel L, Koziolek M, Jung W, et al. Test-enhanced learning of clinical reasoning: a crossover randomised trial. Medical education. 2016;50(7):711-20.
44. Ribeiro LMC, Mamede S, de Brito EM, Moura AS, de Faria RMD, Schmidt HG. Effects of deliberate reflection on students' engagement in learning and learning outcomes. Medical education. 2019;53(4):390-7.
45. Schubach F, Goos M, Fabry G, Vach W, Boeker M. Virtual patients in the acquisition of clinical reasoning skills: does presentation mode matter? A quasi-randomized controlled trial. BMC medical education. 2017;17(1):165.
46. Schuelper N, Ludwig S, Anders S, Raupach T. The Impact of Medical Students' Individual Teaching Format Choice on the Learning Outcome Related to Clinical Reasoning. JMIR medical education. 2019;5(2):e13386.
47. Si J, Kong HH, Lee SH. Developing Clinical Reasoning Skills Through Argumentation With the Concept Map Method in Medical Problem-Based Learning. Interdisciplinary Journal of Problem-Based Learning. 2019;13(1).
48. Sobocan M, Turk N, Dinevski D, Hojs R, Balon BP. Problem-based learning in internal medicine: virtual patients or paper-based problems? Internal Medicine Journal. 2017;47(1):99-103.
49. Stark R, Kopp V, Fischer MR. Case-based learning with worked examples in complex domains: Two experimental studies in undergraduate medical education. Learning and instruction. 2011;21(1):22-33.
50. Stein GH, Tokunaga H, Ando H, Obika M, Miyoshi T, Tokuda Y, et al. Clinical Reasoning Web-based Prototypic Module for Tutors Teaching 5th Grade Medical Students : A Pilot Randomized Study. Journal of General and Family Medicine. 2015;16(1):13-25.
51. Stieger S, Praschinger A, Kletter K, Kainberger F. Diagnostic grand rounds: a new teaching concept to train diagnostic reasoning. European journal of radiology. 2011;78(3):349-52.
52. Weidenbusch M, Lenzer B, Sailer M, Strobel C, Kunisch R, Kiesewetter J, et al. Can clinical case discussions foster clinical reasoning skills in undergraduate medical education? A randomised controlled trial. BMJ open. 2019;9(9):e025973.
53. Xu G, Zhao L, Zhou M. Effectiveness of problem-based learning combined with lecture based learning methodology in renal pathology education. Cogent Education. 2023;10(1).
54. Yousefichaijan P, Jafari F, Kahbazi M, Rafiei M, Pakniyat A. The effect of short-term workshop on improving clinical reasoning skill of medical students. Medical journal of the Islamic Republic of Iran. 2016;30:396.
