# Supplementary material for: Teaching and learning clinical reasoning skill in undergraduate medical students: A scoping review
Source: PLoS One. 2024 Oct 16;19(10):e0309606. doi: 10.1371/journal.pone.0309606 (PMC11482728; doi:10.1371/journal.pone.0309606)
Supplement: S9 Table — (PDF) [file pone.0309606.s012.pdf]

Evidence Snapshot on the effectiveness of interventions to improve clinical reasoning based on the included studies

#	Study	Intervention(s)	Comparison(s)	Main outcome	Level of the Evidence	Effect Size(s): SMD[1] (95% CI), P-value
1	Aghili et al., 2012(1)	traditional educational programs[2] + simulation + feedback	traditional educational programs	Clinical Reasoning Skills	II	SMD: 1.552 (0.928_2.175); P-value= 0.001
2	Alavai-Moghaddam et al., 2024(2)	Case-based clinical reasoning	NA	Improving clinical reasoning	III-3	Significant improvement in CRP, KF_S, and total score (p-value<0.005)
3	Ali et al., 2018(3)	SNAPPS	1- traditional clinical teaching<br>one-minute preceptor	Clinical Reasoning Performance	II	SMD traditional vs OMP: 0.508 (-0.122_1.137); P-value=0.235<br>SMD traditional vs SNAPPS: 1.65 (0.932_-2.367); P value=0.001<br>SMD SNAPPS vs. OMP: 1.013 (0.355 _ 1.672); P-value= 0.005
4	Al Rumayyan et. Al., 2018(4)	hypothetico-deduction	self-explanation	Diagnostic Performance	II	SMD: -0.383 (-0.805 _ 0.039); P-value= 0.04
5	Al Rumayyan et. Al., 2021 (5)	hypothetico-deduction (small group)	self-explanation (Small group)	Diagnostic Performance	II	accurate diagnoses:<br>SMD: 0.353 (0.018 _ 0.688); P-value= 0.03<br>number of diagnostic hypotheses mentioned per case including repetitions:<br>SMD: -1.842 (-2.239_-1.445); p-value= 0.001<br>number of diagnostic hypotheses mentioned per case without repetitions:<br>SMD: -1.571 (-1.951 _ -1.19); P-value= 0.001
6	Bonifacino et al., 2019 (6)	six interactives online modules[3] + a case-based workshop + case based clinical reasoning discussions	case based clinical reasoning discussions	Clinical Reasoning Performance	III-1	Knowledge:<br>SMD: -0.738 (-1.402 _ -0.397); P-Value <0.001<br>Clinical reasoning skill:<br>SMD: -.424 (-0.908 _- 0.061); P-value= 0.09
7	Bösner et al., 2015 (7)	Inverted classroom	NA	Diagnostic Performance	III-3	There was a significant (p < 0.01) overall absolute gain in skills and knowledge of 33%. significant improvement (p < 0.01) in KF & EMQ exam.

---

[1] All SMD were calculated by https://www.psychometrica.de/effect,size.html

[2] didactic lectures, case-based small group discussions, bed-side face to face teaching sessions, and interactive OPD teaching clinics.

[3] The modules included didactic videos, simulated clinical cases, and interactive prompts for open-ended and multiple-choice questions.

8	Braun et al., 2017(8)	CASUS system cases, by representation scaffolding[4]	CASUS system cases without scaffolding	Diagnostic Efficiency	II	Diagnostic efficiency:<br>SMD: -0.461 (-0.884 _-0.038); P-value= 0.045<br>Diagnostic accuracy:<br>SMD: -0.174 (-0.593_0.244); P-value= 0.52
9	Brich et al., 2017(9)	Seminar + TBL	TBL + seminar	acquisition of knowledge and the clinical reasoning skills	II	MCQ Exam:<br>SMD: 0.035 (-0.404_0.473); P-value= 0.89<br>KF_s exam:<br>SMD: 0.424 (-0.019_0.867); P-value= 0.06
10	Carlson et al., 2011(10)	Isabel PRO[5]	NA	Diagnostic Performance	III-3	Average Magnitude of Change Between Pre-Post DAS Scores: 0.07 (0.06); (P < 0.05)
11	Chamberland et al., 2015(11)	listened to examples of residents' SEs with prompts	1. solved word puzzles<br>listened to examples of residents' SEs without prompts	Diagnostic Performance	II	diagnostic accuracy:<br>• training cases<br>SMD resident SE with prompts vs resident SE without prompts: 0.12 (-0.525_0.765)<br>SMD resident SE without prompts vs control group:<br>1.096 (0.385_1.807)<br>SMD resident SE with prompts vs control group:<br>0.972 (0.28_1.664)<br>• near-transfer cases<br>SMD resident SE with prompts vs resident SE without prompts: 0.077 (-0.568,0.722)<br>SMD resident SE with prompts vs control group:<br>0.31 (0.348 _0.969)<br>SMD resident SE without prompts vs control group:<br>0.442 (-0.229_1.113)<br>• far-transfer cases<br>SMD resident SE with prompts vs resident SE without prompts: 0.8 (0.13_1.469)<br>SMD resident SE with prompts vs control group:<br>0.969 (0.277_1.66)<br>SMD resident SE without prompts vs control group:<br>0.291 (-0.376_0.957)<br>diagnostic performance:<br>SMD resident SE with prompts vs resident SE without prompts: 0.989 (0.306_1.672)

[4] interrupting case processing + writing case summaries
[5] a web-based Diagnostic reminder systems by using simulation.

						SMD resident SE with prompts vs control group: 1.601 (0.85_2.353)<br>SMD resident SE without prompts vs control group:<br>0.487 (0.186_1.159)
12	Chamberland et al., 2011(12)	self-explanation	worked with booklets	Diagnostic Performance and Accuracy	=	diagnostic accuracy:<br>less familiar topic:<br>SMD: 0.598 (-0.07_1.265); P-Value < 0.07<br>more familiar topic:<br>SMD: 0.285 (-0.372_0.942); P-value= 0.46<br>diagnostic performance:<br>less familiar topic:<br>SMD: 0.759 (0.083_1.436); P-value= <0.05<br>more familiar topic:<br>SMD: 0.221(-0.434_0.876); P-value=0.44
13	Chamberland et al., 2015(13)	self-explanation + listen to peer self-explanation	1. self-explanation + solve a word puzzle<br>2. self-explanation + listen to expert self-explanation	Diagnostic Performance and Accuracy	=	Diagnostic Accuracy:<br>Posttest:<br>SMD Expert SE vs control: 0.829 (0.136_1.521)<br>SMD Peer SE vs control: 0.391 (-0.289_1.071)<br>SMD Peer SE vs Expert SE: 0.511 (-0.144_1.166)<br>Follow-up:<br>SMD Peer SE vs Expert SE: 0.217 (-0.429_-0.864)<br>SMD Peer SE vs control: 0.112 (-0.562_0.876)<br>SMD Expert SE vs control: 0.062 (-0.734_0.596)<br>Diagnostic Performance<br>Posttest:<br>SMD Peer SE vs Expert SE: 0.028 (-0.616_0.673)<br>SMD Peer SE vs control: 0.363 (-0.316_1.042)<br>SMD Expert SE vs control: 0.436 (-0.237_1.109)<br>Follow-up:<br>SMD Peer SE vs Expert SE: 0.212(-0.435_0.858)<br>SMD Peer SE vs control: 0.46 (-0.222_1.142)<br>SMD Expert SE vs control: 0.271 (-0.397_0.939)
14	Chamberland et al., 2019(14)	immediate feedback + solved clinical cases using self-explanation	1- solved clinical cases using self-explanation<br>delayed feedback + solved clinical cases	Diagnostic Performance and Accuracy	=	Diagnostic Accuracy:<br>Learning cases:<br>SMD Immediate feedback vs delayed feedback: 0.181 (-0.318_0.68)<br>SMD Immediate feedback vs control group:<br>0.07 (-0.424_0.564)

			using self-explanation			SMD delayed feedback vs control group: 0.108 (-0.387_0.602)<br>Near-transfer case:<br>SMD Immediate feedback vs delayed feedback: 0.065 (-0.432_0.563)<br>SMD Immediate feedback vs control group: 0.594 (0.089_1.098)<br>SMD delayed feedback vs control group: 0.496 (-0.005_0.998)<br>Far transfer cases:<br>SMD Immediate feedback vs delayed feedback: 0.034 (-0.464_0.532)<br>SMD Immediate feedback vs control group: 0.194 (-0.301_0.69)<br>SMD delayed feedback vs control group: 0.23 (-0.266_0.726)<br>Diagnostic performance:<br>Learning cases:<br>SMD Immediate feedback vs delayed feedback: 0.019 (-0.479_0.517)<br>SMD Immediate feedback vs control group: 0.207(-0.289_0.702)<br>SMD delayed feedback vs control group: 0.216 (-0.28_0.711)<br>Near-transfer case:<br>SMD Immediate feedback vs delayed feedback: 0.166 (-0.333_0.664)<br>SMD Immediate feedback vs control group: 0.018 (-0.476_0.512)<br>SMD delayed feedback vs control group: 0.124 (-0.37_0.619)<br>Far transfer cases:<br>SMD Immediate feedback vs delayed feedback: 0.082 (-0.416_0.58)<br>SMD Immediate feedback vs control group: 0.176 (-0.318_0.671)<br>SMD delayed feedback vs control group: 0.252 (-0.244_0.748)
15	Choi et al., 2020(15)	Training with reflection and immediate feedback	1- attended outpatient clinic	Diagnostic Accuracy	=	Control set<br>SMD control vs lecture group: 0.474 (-0.04 _0.991)

			attended outpatient clinic + lecture			SMD control vs experiment group: 0.33 (-0.179_0.839)<br>SMD lecture vs experiment group: 0.187 (-0.353_0.726)<br><br>Training set<br>SMD control vs lecture group: 0.07 (-0.441_0.58)<br>SMD control vs experiment group: 1.462(0.894_2.029)<br>SMD lecture vs experiment group: 1.237(0.649_1.825)
16	Delavari et al., 2020(16)	thinking aloud + script-based questioning + test enhanced learning	NA	Clinical Reasoning Performance and Diagnostic Accuracy	III-3	Total diagnostic accuracy score in the pretest and posttest were 5.41(1.16) and 4.91(1.44) respectively (p-value=0.111); total correct discriminating score in the pretest and posttest were 0.41(0.66) and 1.41(2.06) respectively (p-value=0.146)
		thinking aloud + script-based questioning + test enhanced learning + script-based reading		Clinical Reasoning Performance		common KF score in pretest and posttest were 0.4 [0.25-0.78] and 0.9 [0.6-1] respectively (p-value=0.791); discriminative key features score in pretest and posttest were 0.33 [0.16-0.33] and 0.22 [0.11-0.44] respectively (p=0.972)
17	Fernandes et al., 2021(17)	Free reflection	1- Cued reflection Worked example	Diagnostic Accuracy	II	There was a significant primary effect of experimental condition, year of training, and study phase on the diagnostic accuracy achieved (p-value <0.001). Deliberate reflection on the immediate test had significant effect (p-value <0.001). there was no significant difference between the Cued reflection and Worked example groups (p-value> 0.05).
18	Fink et al., 2021 (18)	VP + concluding reflection	1-VP + accompanying reflection<br>2-VP without reflection	diagnostic accuracy	II	Diagnostic accuracy in posttest Mean (SD): Concluding reflection: 0.44 (0.33); accompanying reflection: 0.37 (0.31); control group: 0.34 (0.33); p-value: 0.36<br>SMD Concluding reflection *vs* accompanying reflection:<br>0.218 (-0.219_0.656)<br>SMD accompanying reflection *vs* control group:<br>0.94 (-0.348_0.535)

						SMD control group *vs* Concluding reflection: 0.303 (-0.132_0.739)
19	Gong et al., 2022 (19)	TBL + Feedback + reflection + summarizing key points and commented on students' performance by teacher	Traditional bedside teaching	Clinical Reasoning Performance	=	SMD: 1.241(0.136_2.346); P-value= 0.002
20	Heitzmann et al., 2015(20)	self-explanation + adaptable feedback + reflection on the error	1. Without self-explanation + Adaptable feedback<br>2. self-explanation + Without adaptable feedback+ reflect on the error without self-explanation + Without adaptable feedback	Diagnostic competency	=	Adaptable feedback improved different aspects of diagnostic competency:<br>Strategic Knowledge:<br>F (1,93) = 4.15, p < 0.05, partial g2 = 0.04<br>Decision-oriented practical knowledge:<br>F (1,93) = 4.41, p < 0.05, partial g2 = 0.05)
21	Ibiapina et al., 2014(21)	Modelled reflection	1- cued reflection<br>2- free reflection	Diagnostic accuracy	=	Immediate test:<br>modelled reflection vs Cued reflection:<br>SMD=0.044(-0.584_0.677)<br>modelled reflection vs free reflection:<br>SMD=0.716 (0.069_1.364)<br>Fallow-up:<br>modelled reflection vs Cued reflection:<br>SMD=0.249(-0.381_0.879)<br>modelled reflection vs free reflection:<br>SMD=0.805 (0.152_1.457)<br>Immediate test:<br>modelled reflection vs Cued reflection:<br>SMD=0(-0.665_0.665)<br>modelled reflection vs free reflection:<br>SMD=0.762 (0.127_1.398)<br>Fallow-up:<br>modelled reflection vs Cued reflection:<br>SMD=0.138(-0.528_0.804)<br>modelled reflection vs free reflection:<br>SMD=0.737 (0.103_1.372)

22	Jost et al., 2017(22)	seminar[6] + TBL	seminar	Clinical Reasoning Performance	III-2	SMD: 0.805 (-0.003_1.613); P-value= 0.026
23	Kahl et al., 2022 (23)	Lectures + PBL + IHT + Video Film	Lectures + PBL + TAU	Diagnostic performance	II	SMD: 1.17 (0.441_1.899); P-Value < 0.001
24	Kıyak et al., 2022 (24)	Test enhanced learning + feedback	Test enhanced learning + feedback	Clinical Reasoning Performance	II	The Intervention group pretest and posttest median were 23.75 and 88.50 respectively (p-value<0.001); comparison group pretest and posttest median were 23.05 and 17 respectively (p-value:0.30)
25	Kiesewetter et al., 2020(25)	serial cue (VPs in CASUS system)	whole case (VPS in CASUS system)	Diagnostic accuracy	II	Diagnosis Performance<br>SMD: 0.545 (0.072_1.019); p-value < 0.01<br>Diagnostic Accuracy<br>SMD: 0.286 (-0.182_0.753); p-value= 0.22
26	Klein et al., 2019(26)	The unsupported-example-condition	1. The closed prompt-condition<br>The open-prompt-condition	Clinical Reasoning Performance	II	SMD Unsupported-example condition vs Closed-prompt-condition: 0.447 (-0.042_0.935)<br>SMD Unsupported-example condition vs Open-prompt-condition: 0.185 (-0.303_0.672)<br>SMD Closed-prompt-condition vs Open-prompt-condition:<br>0.242 (-0.247_0.73)
27	Kuhn et al., 2023 (27)	Deliberate reflection	Read case and give diagnosis	Clinical Reasoning Performance	III-2	Proportion of recalled discriminating features for alternative diagnosis:<br>deliberate reflection condition Mean (SD): 0.60 (0.14); Control group Mean (SD): 0.53 (0.18); p-value:0.13<br>SMD: 0.433 (0.069_0.796)
28	Lee et al., 2010(28)	Lecture + teaching illness script[7]	self-directed study	Clinical Reasoning Skill	II	DTI<br>SMD: 0.055 (-0.486_0.596)<br>CRP<br>SMD: 1.241 (0.651_1.831)
29	Linsen et al., 2018(29)	solving a clinical case using a written description of a patient encounter +individual study	solving the clinical case using a video patient encounter + group discussion	Diagnostic Performance	II	SMD: 0.121 (-0.094_0.336); p-value= 0.23
30	Ludwig et al., 2018(30)	test enhanced learning + e-seminar + watching videos	test enhanced learning + e-seminar + read text cases	Clinical Reasoning Performance	II	Students in the video-based group obtained higher scores in comparison with the text-based group (76.2 ± 19.4% vs. 72.4 ± 19.1%, p

[6] regular teaching
[7] test-enhanced learning + think aloud + feedback.

						= 0.026). However, this effect was not observed in the retention test(69.2 ± 20.2% vs. 66.4 ± 20.3%, p = 0.108).
31	Mamede et al., 2012(31)	Reflection (Structured)	1. the immediate diagnosis condition<br>2. the differential diagnosis condition	Diagnostic Performance	=	Immediate test<br>Immediate diagnosis group Mean (95% CI): 0.61 (0.52_0.70) Differential diagnosis group Mean (95% CI): 0.62 (0.54_0.70) Structured reflection group Mean (95% CI): 0.48 (0.38_0.58) Reflection vs Immediate diagnosis p-value: 0.028; Reflection vs differential diagnosis p-value: 0.82.<br>Delayed test<br>Immediate diagnosis group Mean (95% CI): 0.52 (0.43_0.60)<br>Differential diagnosis group Mean (95% CI): 0.48 (0.37_0.58) Structured reflection group Mean (95% CI): 0.66 (0.56_0.76)<br>Reflection vs Immediate diagnosis p-value: 0.013<br>Reflection vs differential diagnosis p-value: 0.005
32	Mamede et al., 2014 (32)	Reflection (Structured)	1. Single diagnosis[8] + solve a word puzzle<br>Differential diagnosis + solve a word puzzle	Diagnostic Accuracy and Performance	=	Global performance in the test (all criterion cases): p-value <0.001<br>Nonanalytical reasoning Range (95% CI): 0.34 (0.28–0.39) Differential diagnosis Range (95% CI): 0.39 (0.32–0.46) Structured reflection Range (95% CI): 0.51 (0.46–0.56)<br>Performance in the test on cases of previously studied diseases: p-value <0.001<br>Nonanalytical reasoning Range (95% CI): 0.36 (0.26–0.46)<br>Differential diagnosis Range (95% CI): 0.51 (0.40–0.63)<br>Structured reflection Range (95% CI): 0.67 (0.60–0.74)<br>Performance in the test on cases of non-previously studied diseases: p-value: 0.32<br>Nonanalytical reasoning Range (95% CI): 0.32 (0.26–0.38)

[8] Immediate decision

						Differential diagnosis Range (95% CI): 0.33 (0.25–0.40)<br>Structured reflection Range (95% CI): 0.44 (0.36–0.51)
33	Mamede et al., 2019 (33)	Reflection(free)	1. Cued reflection Modelled reflection	Diagnostic Accuracy and Performance	II	Diagnostic accuracy<br>Cued reflection and free reflection: SMD= 0.789 (0.224_1.353), p-value <0.05<br>Cued reflection and modelled reflection: SMD= 0.178 (-0.357_0.713), p-value > 0.05<br>free-reflection and modelled reflection: SMD= 0.617 (0.071_1.164), p-value > 0.05
34	Matinpour et al., 2014(34)	PBL workshop[9] + conventional training	conventional training	Clinical Reasoning Performance	III-2	SMD: 0.21 (-0.289_0.71)
35	Middeke et al., 2018 (35)	PBL	Serious game / EMERGE	Clinical Reasoning Performance	III-2	PBL group had lower scores than the serious game group (p-value < 0.05).
36	Mlika et al., 2023(36)	CRT	SNAPPS	Clinical reasoning competency	II	CRT arm Mean (SD): 4.62 (2.93), SNAPPS arm Mean (SD): 4.99(2.93); p-value > 0.05
37	Moghadami et al., 2021(37)	Think aloud+ small group discussion	traditional lecture +small group discussion	Diagnostic Performance	II	SMD: 0.537 (-0.027_1.102); p-value= 0.009
38	Mutter et al., 2020(38)	CS-M	CS-NM	Clinical Reasoning Performance	II	SMD: 0.644 (0.064_1.224); p-value= 0.003
39	Oliveira et al., 2022 (39)	Individual study + Structured reflection + Identification and association exercises + Mind maps + Application to the resolution of clinical cases	Individual study + Structured reflection + Identification and association exercises + Mind maps + Application to the resolution of clinical cases	Diagnostic Accuracy	II	Training improved diagnostic accuracy and reduced case resolution time in both groups for specific diagnoses, but there was no transfer of learning to new cases.<br>group 1 = 1.00 [0.00 to 1.00] versus 2.00 SD[2.00 to 2.50], p = 0.017 and group 2 = 1.00 [0.66 to 1.17] versus 3.00 [1.33 to 3.00], p= 0.006
40	Ong et al., 2022 (40)	sTBL	IL	Clinical Reasoning Performance	II	neuroanatomical localization subsection (7 scenarios):<br>Mean difference (%) (95% CI): 3.1 (0.6–5.5); P-value= 0.013

[9] clinical reasoning education (PBL) using patient scenarios.

						Neurological emergencies subsection (7 scenarios):<br>Mean difference (%) (95% CI): -0.4 (-2.2 to 3.1); P-value= 0.75
41	PEAHL et al., 2019 (41)	postpartum rounding video + usual teaching	usual teaching	Clinical Reasoning Performance	II	Differential diagnosis<br>SMD: 0.1 (-0.345_0.545); p-value= 0.43
42	Peixoto et al., 2017(42)	test enhanced learning + self-explanation	test enhanced learning	Diagnostic Performance	II	SMD: 0.143 (-0.486_0.772); p-value= 0.66
43	Raupach et al., 2016(43)	e-seminar (test enhanced learning + feedback)	e-seminar (studying long case narratives[10] + feedback)	Clinical Reasoning Performance	II	Mean (SD) scores for test items: 56.0 (25.8%); Mean (SD) for study-only item: 48.8 (24.7%); p < 0.001)
44	Ribeiro et al., 2019 (44)	Deliberate Reflection + case study	Making differential diagnosis + case study	diagnostic performance	II	Deliberate reflection Mean (SD): 22.08 (14.94)<br>Making differential diagnosis Mean (SD): 15.75 (9.24)<br>p-value: 0.03; SMD: 0.51 (0.04_0.979)
45	Schubach et al., 2017(45)	key feature[11]	systematic[12]	Clinical Reasoning Performance	III-2	SCT Result: gastrointestinal hemorrhage subscale<br>SMD (according to ITT): 0.05 (-0.475_0.575)<br>KF vs. systematic arm difference [95% CI]<br>ITT: 0.23 [−3.01_3.46], p-value= 0.888<br>PP: 0 [−3.48_3.48], p-value= 1<br>SCT Result: acute abdomen subscale<br>SMD (according to ITT): 0.336 (-0.193_0.864)<br>KF vs. systematic arm difference [95% CI]<br>ITT: −5.32 [−13.79_3.15], p-value= 0.214<br>PP: −5.47 [−14.81_3.88], p-value: 0.245
46	Schuelper et al., 2019(46)	test enhanced learning (video-based key feature questions)	test enhanced learning (text-based key feature questions)	Clinical Reasoning Performance	III-2	Post test:<br>Video-based Mean score% (SD): 76.2% (12.6); Text-based Mean score% (SD): 70% (19); p-value: 0.15<br>Follow-up:<br>Video-based Mean score% (SD): 75.3% (16.6); Text-based Mean score% (SD): 63.4% (20.3); P-value: 0.02

[10] case-based learning

[11] virtual patients + worked on multiple short cases, with the instruction being focused only on important elements.

[12] virtual patients + worked on few long cases, with the instruction being comprehensive and systematic.

47	Si et al., 2019(47)	PBL + Argumentation with the Concept Map Method	NA	Clinical Reasoning Performance	III-2	The clinical reasoning performance increased in both first year (p-value<0.001) and second-year students(P-value<0.001).
48	Sobocan et al., 2017(48)	digital problem-based learning[13]	paper problem-based learning	Diagnostic thinking ability	=	No significant difference in knowledge, flexibility in thinking, memory structure was observed between digital PBL(VPs) and p-PBL groups.
49	Stark et al. 2011 (49)	with errors and elaborated feedback	1- with errors and KOR feedback<br>2- without errors and elaborated feedback<br>3- without errors and KOR-feedback	Diagnostic competency	=	Strategic knowledge Mean (SD):<br>with errors and elaborated feedback: 20.68 (3.02)<br>with errors and KOR feedback: 17.81 (2.96)<br>without errors and elaborated feedback: 18.32 (4.04)<br>without errors and KOR-feedback: 18.85 (4.42)<br>conditional knowledge Mean (SD):<br>with errors and elaborated feedback: 11.93 (3.65)<br>with errors and KOR feedback: 9.93 (3.53)<br>without errors and elaborated feedback: 10.86 (3.70)<br>without errors and KOR-feedback: 11.63 (3.27)<br>Strategic knowledge Mean (SD):<br>with errors and elaborated feedback: 26.40 (3.27)<br>with errors and KOR feedback: 23.25 (3.71)<br>without errors and elaborated feedback: 25.76 (4.10)<br>without errors and KOR-feedback: 25.29 (3.56)<br>conditional knowledge Mean (SD):<br>with errors and elaborated feedback: 13.99 (4.05)<br>with errors and KOR feedback: 9.68 (4.56)<br>without errors and elaborated feedback: 13.49 (5.39)<br>without errors and KOR-feedback: 10.71 (4.22)
50	Stein et al., 2015(50)	self-study	Tutored syllabus	Clinical Reasoning Performance	=	University 1<br>SMD: 0.847 (-0.824_2.517)<br>University 2<br>SMD: 0.712 (-0.938_2.363)

[13] virtual patients

51	Stieger et al., 2011 (51)	interactive discussions	NA	diagnostic performance	III-3	DTI: Pretest mean: 165.1, posttest mean: 176.4, p-value: <0.001
52	Weidenbusch et al., 2019(52)	peer-moderated live case discussions in an interactive setting	1. single-learner format utilizing an interactive multimedia platform displaying video recordings of the live case discussion<br>2. single learner format in which the students worked with the original paper cases of the NEJM	Clinical Reasoning Performance	II	Posttest:<br>Live-CCD vs video-CCD SMD: 0.724 (0.187-_1.26); P-value=0.011<br>Live-CCD vs paper-cases SMD=1.936 (1.337_2.535); P-Value<0.001<br>Video-CCD vs paper-cases SMD: 1.108 (0.562_1.654); P-Value <0.001<br>Follow-up:<br>Live-CCD vs video-CCD SMD=0.492 (-0.035_1.02); P-value= 0.146<br>Live-CCD vs paper-cases SMD=1.933 (1.334_2.532); P-Value <0.001<br>video-CCD vs paper-cases SMD=1.49 (0.916_2.064); P-Value <0.001
53	Xu et al., 2023 (53)	PBL + lecture	Lecture	Diagnostic accuracy	II	Diagnostic accuracy in PBL + lecture group=81%; in lecture group=58.5%; P-value<0.001
54	Yousefichaijan et al., 2016(54)	Lecture on the nature and process of clinical reasoning	No intervention	Clinical Reasoning performance	III-2	No significant difference was observed.

References:

1. Aghili O, Khamseh ME, Taghavinia M, Malek M, Emami Z, Baradaran HR, et al. Virtual patient simulation: Promotion of clinical reasoning abilities of medical students. Knowledge Management and E-Learning. 2012;4(4):518-27.
2. Alavi-Moghaddam M, Zeinaddini-Meymand A, Ahmadi S, Shirani A. Teaching clinical reasoning to medical students: A brief report of case-based clinical reasoning approach. Journal of education and health promotion. 2024;13(1):42.
3. Ali S, Jamil B, Ali L. EFFECTIVENESS OF VARIOUS TEACHING METHODOLOGIES IN DEVELOPING CLINICAL REASONING SKILLS IN UNDERGRADUATE FEMALE MEDICAL STUDENTS. Khyber Medical University Journal-Kmuj. 2018;10(2):71-5.
4. Al Rumayyan A, Ahmed N, Al Subait R, Al Ghamdi G, Mahzari MM, Mohamed TA, et al. Teaching clinical reasoning through hypothetico-deduction is (slightly) better than self-explanation in tutorial groups: An experimental study. Perspectives on Medical Education. 2018;7(2):93-9.
5. Al Rumayyan A, Mamede S, van Mook WNKA, Schmidt HG. Teaching Clinical Reasoning: An Experiment Comparing the Effects of Small-group Hypothetico-deduction Versus Self-explanation. Health Professions Education. 2021;7(1):12-9.
6. Bonifacino E, Follansbee WP, Farkas AH, Jeong K, McNeil MA, DiNardo DJ. Implementation of a clinical reasoning curriculum for clerkship-level medical students: a pseudo-randomized and controlled study. Diagnosis (Berlin, Germany). 2019;6(2):165-72.
7. Bösner S, Pickert J, Stibane T. Teaching differential diagnosis in primary care using an inverted classroom approach: student satisfaction and gain in skills and knowledge. BMC medical education. 2015;15:63.
8. Braun LT, Zottmann JM, Adolf C, Lottspeich C, Then C, Wirth S, et al. Representation scaffolds improve diagnostic efficiency in medical students. Medical education. 2017;51(11):1118-26.
9. Brich J, Jost M, Brustle P, Giesler M, Rijntjes M. Teaching neurology to medical students with a simplified version of team-based learning. Neurology. 2017;89(6):616-22.
10. Carlson J, Abel M, Bridges D, Tomkowiak J. The Impact of a Diagnostic Reminder System on Student Clinical Reasoning During Simulated Case Studies. Simulation in Healthcare-Journal of the Society for Simulation in Healthcare. 2011;6(1):11-7.
11. Chamberland M, Mamede S, St-Onge C, Setrakian J, Bergeron L, Schmidt H. Self-explanation in learning clinical reasoning: the added value of examples and prompts. Medical education. 2015;49(2):193-202.
12. Chamberland M, St-Onge C, Setrakian J, Lanthier L, Bergeron L, Bourget A, et al. The influence of medical students' self-explanations on diagnostic performance. Medical education. 2011;45(7):688-95.
13. Chamberland M, Mamede S, St-Onge C, Setrakian J, Schmidt HG. Does medical students' diagnostic performance improve by observing examples of self-explanation provided by peers or experts? Advances in Health Sciences Education. 2015;20(4):981-93.
14. Chamberland M, Setrakian J, St-Onge C, Bergeron L, Mamede S, Schmidt HG. Does providing the correct diagnosis as feedback after self-explanation improve medical students diagnostic performance? BMC medical education. 2019;19(1):194.

15. Choi S, Oh S, Lee DH, Yoon HS. Effects of reflection and immediate feedback to improve clinical reasoning of medical students in the assessment of dermatologic conditions: a randomised controlled trial. BMC medical education. 2020;20(1):146.
16. Delavari S, Monajemi A, Baradaran HR, Myint PK, Yaghmaei M, Soltani Arabshahi SK. How to develop clinical reasoning in medical students and interns based on illness script theory: An experimental study. Medical journal of the Islamic Republic of Iran. 2020;34:9.
17. Fernandes RAF, Malloy-Diniz LF, de Vasconcellos MC, Camargos PAM, Ibiapina C. Adding guidance to deliberate reflection improves medical student's diagnostic accuracy. Medical education. 2021;55(10):1161-71.
18. Fink MC, Heitzmann N, Siebeck M, Fischer F, Fischer MR. Learning to diagnose accurately through virtual patients: do reflection phases have an added benefit? Bmc Medical Education. 2021;21(1).
19. Gong J, Du J, Hao J, Li L. Effects of bedside team-based learning on pediatric clinical practice in Chinese medical students. BMC medical education. 2022;22(1):264.
20. Heitzmann N, Fischer F, Kühne-Eversmann L, Fischer MR. Enhancing diagnostic competence with self-explanation prompts and adaptable feedback. Medical education. 2015;49(10):993-1003.
21. Ibiapina C, Mamede S, Moura A, Elói-Santos S, van Gog T. Effects of free, cued and modelled reflection on medical students' diagnostic competence. Medical Education. 2014;48(8):796-805.
22. Jost M, Brüstle P, Giesler M, Rijntjes M, Brich J. Effects of additional team-based learning on students' clinical reasoning skills: a pilot study. BMC research notes. 2017;10(1):282.
23. Kahl KG, Alte C, Sipos V, Kordon A, Hohagen F, Schweiger U. A randomized study of iterative hypothesis testing in undergraduate psychiatric education. Acta Psychiatr Scand. 2010;122(4):334-8.
24. Kiyak YS, Budakoglu, II, Kalaycioglu DB, Kula S, Coskun O. Can preclinical students improve their clinical reasoning skills only by taking case-based online testlets? A randomized controlled study. Innovations in Education and Teaching International. 2022.
25. Kiesewetter J, Sailer M, Jung VM, Schönberger R, Bauer E, Zottmann JM, et al. Learning clinical reasoning: how virtual patient case format and prior knowledge interact. BMC Medical Education. 2020;20(1):1-10.
26. Klein M, Otto B, Fischer MR, Stark R. Fostering medical students' clinical reasoning by learning from errors in clinical case vignettes: effects and conditions of additional prompting procedures to foster self-explanations. Advances in Health Sciences Education. 2019;24(2):331-51.
27. Kuhn J, Mamede S, van den Berg P, Zwaan L, Elshout G, Bindels P, et al. Teaching medical students to apply deliberate reflection. Medical teacher. 2023;46(1):65-72.
28. Lee A, Joynt GM, Lee AK, Ho AM, Groves M, Vlantis AC, et al. Using illness scripts to teach clinical reasoning skills to medical students. Family medicine. 2010;42(4):255-61.
29. Linsen A, Elshout G, Pols D, Zwaan L, Mamede S. Education in clinical reasoning: an experimental study on strategies to foster novice medical students' engagement in learning activities. Health Professions Education. 2018;4(2):86-96.
30. Ludwig S, Schuelper N, Brown J, Anders S, Raupach T. How can we teach medical students to choose wisely? A randomised controlled cross-over study of video- versus text-based case scenarios. BMC medicine. 2018;16(1):107.
31. Mamede S, van Gog T, Moura AS, de Faria RM, Peixoto JM, Rikers RM, et al. Reflection as a strategy to foster medical students' acquisition of diagnostic competence. Medical education. 2012;46(5):464-72.
32. Mamede S, van Gog T, Sampaio AM, de Faria RM, Maria JP, Schmidt HG. How can students' diagnostic competence benefit most from practice with clinical cases? The effects of structured reflection on future diagnosis of the same and novel diseases. Academic medicine : journal of the Association of American Medical Colleges. 2014;89(1):121-7.

33. Mamede S, Figueiredo-Soares T, Elói Santos SM, de Faria RMD, Schmidt HG, van Gog T. Fostering novice students' diagnostic ability: the value of guiding deliberate reflection. Medical education. 2019;53(6):628-37.
34. Matinpour M, Sedighi I, Monajemi A, Jafari F, Momtaz HE, Ali Seif Rabiei M. Clinical reasoning and improvement in the quality of medical education. Shiraz E Medical Journal. 2014;15(4):1-4.
35. Middeke A, Anders S, Schuelper M, Raupach T, Schuelper N. Training of clinical reasoning with a Serious Game versus small-group problem-based learning: A prospective study. PloS one. 2018;13(9):e0203851.
36. Mlika M, Dziri C, Jallouli M, Cheikhrouhou S, Mezni F. Teaching clinical reasoning among undergraduate medical. Journal of Medical Education Development. 2023;16(51):57-64.
37. Moghadami M, Amini M, Moghadami M, Dalal B, Charlin B. Teaching clinical reasoning to undergraduate medical students by illness script method: a randomized controlled trial. BMC medical education. 2021;21(1):87.
38. Mutter MK, Martindale JR, Shah N, Gusic ME, Wolf SJ. Case-Based Teaching: Does the Addition of High-Fidelity Simulation Make a Difference in Medical Students' Clinical Reasoning Skills? Medical science educator. 2020;30(1):307-13.
39. Oliveira JCV, Peixoto AB, Marinho GEM, Peixoto JM. Teaching of Clinical Reasoning Guided by Illness Script Theory. Arquivos Brasileiros de Cardiologia. 2022;119(5):14-21.
40. Ong KY, Ng CWQ, Tan NCK, Tan K. Differential effects of team-based learning on clinical reasoning. The clinical teacher. 2022;19(1):17-23.
41. Peahl AF, Tarr EE, Has P, Hampton BS. Impact of 4 Components of Instructional Design Video on Medical Student Medical Decision Making During the Inpatient Rounding Experience. Journal of surgical education. 2019;76(5):1286-92.
42. Peixoto JM, Mamede S, de Faria RMD, Moura AS, Santos SME, Schmidt HG. The effect of self-explanation of pathophysiological mechanisms of diseases on medical students' diagnostic performance. Advances in Health Sciences Education. 2017;22(5):1183-97.
43. Raupach T, Andresen JC, Meyer K, Strobel L, Koziolek M, Jung W, et al. Test-enhanced learning of clinical reasoning: a crossover randomised trial. Medical education. 2016;50(7):711-20.
44. Ribeiro LMC, Mamede S, de Brito EM, Moura AS, de Faria RMD, Schmidt HG. Effects of deliberate reflection on students' engagement in learning and learning outcomes. Medical education. 2019;53(4):390-7.
45. Schubach F, Goos M, Fabry G, Vach W, Boeker M. Virtual patients in the acquisition of clinical reasoning skills: does presentation mode matter? A quasi-randomized controlled trial. BMC medical education. 2017;17(1):165.
46. Schuelper N, Ludwig S, Anders S, Raupach T. The Impact of Medical Students' Individual Teaching Format Choice on the Learning Outcome Related to Clinical Reasoning. JMIR medical education. 2019;5(2):e13386.
47. Si J, Kong HH, Lee SH. Developing Clinical Reasoning Skills Through Argumentation With the Concept Map Method in Medical Problem-Based Learning. Interdisciplinary Journal of Problem-Based Learning. 2019;13(1).
48. Sobocan M, Turk N, Dinevski D, Hojs R, Balon BP. Problem-based learning in internal medicine: virtual patients or paper-based problems? Internal Medicine Journal. 2017;47(1):99-103.
49. Stark R, Kopp V, Fischer MR. Case-based learning with worked examples in complex domains: Two experimental studies in undergraduate medical education. Learning and instruction. 2011;21(1):22-33.
50. Stein GH, Tokunaga H, Ando H, Obika M, Miyoshi T, Tokuda Y, et al. Clinical Reasoning Web-based Prototypic Module for Tutors Teaching 5th Grade Medical Students : A Pilot Randomized Study. Journal of General and Family Medicine. 2015;16(1):13-25.
51. Stieger S, Praschinger A, Kletter K, Kainberger F. Diagnostic grand rounds: a new teaching concept to train diagnostic reasoning. European journal of radiology. 2011;78(3):349-52.

52. Weidenbusch M, Lenzer B, Sailer M, Strobel C, Kunisch R, Kiesewetter J, et al. Can clinical case discussions foster clinical reasoning skills in undergraduate medical education? A randomised controlled trial. BMJ open. 2019;9(9):e025973.
53. Xu G, Zhao L, Zhou M. Effectiveness of problem-based learning combined with lecture based learning methodology in renal pathology education. Cogent Education. 2023;10(1).
54. Yousefichaijan P, Jafari F, Kahbazi M, Rafiei M, Pakniyat A. The effect of short-term workshop on improving clinical reasoning skill of medical students. Medical journal of the Islamic Republic of Iran. 2016;30:396.
