# Supplementary material for: Teaching and learning clinical reasoning skill in undergraduate medical students: A scoping review
Source: PLoS One. 2024 Oct 16;19(10):e0309606. doi: 10.1371/journal.pone.0309606 (PMC11482728; doi:10.1371/journal.pone.0309606)
Supplement: S10 Table — (PDF) [file pone.0309606.s013.pdf]

Critical appraisal of included studies by MMAT

#	study	Type of study	S1	S2	RCT					Nonrandomized				
					2.1	2.2	2.3	2.4	2.5	3.1	3.2	3.3	3.4	3.5
1	Aghili et al., 2012 (1)	RCT												
2	Alavi-Moghadam et al., 2024 (2)	One group before-after												
3	Ali et al., 2018(3)	RCT												
4	Al Rumayyan et. al., 2018(4)	RCT												
5	Al Rumayyan et. al., 2021 (5)	RCT												
6	Bonifacino et al., 2019 (6)	Non-randomized controlled study												
7	Bösner et al., 2015 (7)	one-group before-after												
8	Braun et al., 2017(8)	RCT												
9	Brich et al., 2017(9)	RCT												
10	Carlson et al., 2011(10)	one group before-after												
11	Chamberland et al., 2015(11)	RCT												
12	Chamberland et al., 2011(12)	RCT												
13	Chamberland et al., 2015(13)	RCT												
14	Chamberland et al., 2019(14)	RCT												
15	Choi et al., 2020(15)	RCT												
16	Delavari et al., 2020(16)	One group before-after												
17	Fernandes et al., 2021(17)	RCT												
18	Fink et al., 2021 (18)	RCT												
19	Gong et al., 2022 (19)	RCT												
20	Heitzmann et al., 2015(20)	RCT												
21	Ibiapina et al., 2014 (21)	RCT												
22	Jost et al., 2017(22)	quasi-Experimental												
23	Kahl et al., 2022 (23)	RCT												

24	Kıyak et al., 2022 (24)	RCT												
25	Kiesewetter et al., 2020(25)	RCT												
26	Klein et al., 2019(26)	RCT												
27	Kuhn et al., 2023 (27)	Quasi-experimental												
28	Lee et al., 2010(28)	RCT												
29	Linsen et al., 2018(29)	RCT												
30	Ludwig et al., 2018(30)	RCT												
31	Mamede et al., 2012(31)	RCT												
32	Mamede et al., 2014 (32)	RCT												
33	Mamede et al., 2019 (33)	RCT												
34	Matinpour et al., 2014(34)	Quasi-experimental												
35	Middeke et al., 2018 (35)	Quasi-experimental												
36	Mlika et al., 2023(36)	RCT												
37	Moghadami et al., 2021(37)	RCT												
38	Mutter et al., 2020(38)	RCT												
39	Oliveira et al, 2022 (39)	RCT												
40	Ong et al., 2022 (40)	RCT												
41	PEAHL et al., 2019(41)	RCT												
42	Peixoto et al., 2017(42)	RCT												
43	Raupach et al., 2016(43)	RCT												
44	Ribeiro et al., 2019 (44)	RCT												
45	Schubach et al., 2017(45)	Quazi-randomized control trial												
46	Schuelper et al., 2019(46)	Quasi-experimental												
47	Si et al., 2019(47)	One group pretest & posttest												
48	Sobocan et al., 2017(48)	RCT												
49	Stark et al., (2011) (49)	RCT												

50	Stein et al., 2015(50)	RCT	Yes	Yes	Can't tell	Yes	Yes	Yes	Can't tell					
51	Stieger et al., 2011 (51)	One group before-after	Yes	Yes						Can't tell	Yes	No	No	Can't tell
52	Weidenbusch et al., 2019(52)	RCT	Yes	Yes	Yes	No	Yes	Can't tell	Can't tell					
53	Xu et al. 2023(53)	RCT	Yes	Yes	Yes	Yes	Yes	Can't tell	Can't tell					
54	Yousefichaijan et al., 2016(54)	Quasi-experimental	Yes	Yes						Can't tell	Yes	Yes	No	Can't tell
	**Guide**	Yes: (green)<br>No: (red)<br>Can't tell: (yellow)												

References:

1. Aghili O, Khamseh ME, Taghavinia M, Malek M, Emami Z, Baradaran HR, et al. Virtual patient simulation: Promotion of clinical reasoning abilities of medical students. Knowledge Management and E-Learning. 2012;4(4):518-27.
2. Alavi-Moghaddam M, Zeinaddini-Meymand A, Ahmadi S, Shirani A. Teaching clinical reasoning to medical students: A brief report of case-based clinical reasoning approach. Journal of education and health promotion. 2024;13(1):42.
3. Ali S, Jamil B, Ali L. EFFECTIVENESS OF VARIOUS TEACHING METHODOLOGIES IN DEVELOPING CLINICAL REASONING SKILLS IN UNDERGRADUATE FEMALE MEDICAL STUDENTS. Khyber Medical University Journal-Kmuj. 2018;10(2):71-5.
4. Al Rumayyan A, Ahmed N, Al Subait R, Al Ghamdi G, Mahzari MM, Mohamed TA, et al. Teaching clinical reasoning through hypothetico-deduction is (slightly) better than self-explanation in tutorial groups: An experimental study. Perspectives on Medical Education. 2018;7(2):93-9.
5. Al Rumayyan A, Mamede S, van Mook WNKA, Schmidt HG. Teaching Clinical Reasoning: An Experiment Comparing the Effects of Small-group Hypothetico-deduction Versus Self-explanation. Health Professions Education. 2021;7(1):12-9.
6. Bonifacino E, Follansbee WP, Farkas AH, Jeong K, McNeil MA, DiNardo DJ. Implementation of a clinical reasoning curriculum for clerkship-level medical students: a pseudo-randomized and controlled study. Diagnosis (Berlin, Germany). 2019;6(2):165-72.
7. Bösner S, Pickert J, Stibane T. Teaching differential diagnosis in primary care using an inverted classroom approach: student satisfaction and gain in skills and knowledge. BMC medical education. 2015;15:63.
8. Braun LT, Zottmann JM, Adolf C, Lottspeich C, Then C, Wirth S, et al. Representation scaffolds improve diagnostic efficiency in medical students. Medical education. 2017;51(11):1118-26.
9. Brich J, Jost M, Brustle P, Giesler M, Rijntjes M. Teaching neurology to medical students with a simplified version of team-based learning. Neurology. 2017;89(6):616-22.
10. Carlson J, Abel M, Bridges D, Tomkowiak J. The Impact of a Diagnostic Reminder System on Student Clinical Reasoning During Simulated Case Studies. Simulation in Healthcare-Journal of the Society for Simulation in Healthcare. 2011;6(1):11-7.
11. Chamberland M, Mamede S, St-Onge C, Setrakian J, Bergeron L, Schmidt H. Self-explanation in learning clinical reasoning: the added value of examples and prompts. Medical education. 2015;49(2):193-202.
12. Chamberland M, St-Onge C, Setrakian J, Lanthier L, Bergeron L, Bourget A, et al. The influence of medical students' self-explanations on diagnostic performance. Medical education. 2011;45(7):688-95.
13. Chamberland M, Mamede S, St-Onge C, Setrakian J, Schmidt HG. Does medical students' diagnostic performance improve by observing examples of self-explanation provided by peers or experts? Advances in Health Sciences Education. 2015;20(4):981-93.
14. Chamberland M, Setrakian J, St-Onge C, Bergeron L, Mamede S, Schmidt HG. Does providing the correct diagnosis as feedback after self-explanation improve medical students diagnostic performance? BMC medical education. 2019;19(1):194.
15. Choi S, Oh S, Lee DH, Yoon HS. Effects of reflection and immediate feedback to improve clinical reasoning of medical students in the assessment of dermatologic conditions: a randomised controlled trial. BMC medical education. 2020;20(1):146.
16. Delavari S, Monajemi A, Baradaran HR, Myint PK, Yaghmaei M, Soltani Arabshahi SK. How to develop clinical reasoning in medical students and interns based on illness script theory: An experimental study. Medical journal of the Islamic Republic of Iran. 2020;34:9.

17. Fernandes RAF, Malloy-Diniz LF, de Vasconcellos MC, Camargos PAM, Ibiapina C. Adding guidance to deliberate reflection improves medical student's diagnostic accuracy. Medical education. 2021;55(10):1161-71.
18. Fink MC, Heitzmann N, Siebeck M, Fischer F, Fischer MR. Learning to diagnose accurately through virtual patients: do reflection phases have an added benefit? Bmc Medical Education. 2021;21(1).
19. Gong J, Du J, Hao J, Li L. Effects of bedside team-based learning on pediatric clinical practice in Chinese medical students. BMC medical education. 2022;22(1):264.
20. Heitzmann N, Fischer F, Kühne-Eversmann L, Fischer MR. Enhancing diagnostic competence with self-explanation prompts and adaptable feedback. Medical education. 2015;49(10):993-1003.
21. Ibiapina C, Mamede S, Moura A, Elói-Santos S, van Gog T. Effects of free, cued and modelled reflection on medical students' diagnostic competence. Medical Education. 2014;48(8):796-805.
22. Jost M, Brüstle P, Giesler M, Rijntjes M, Brich J. Effects of additional team-based learning on students' clinical reasoning skills: a pilot study. BMC research notes. 2017;10(1):282.
23. Kahl KG, Alte C, Sipos V, Kordon A, Hohagen F, Schweiger U. A randomized study of iterative hypothesis testing in undergraduate psychiatric education. Acta Psychiatr Scand. 2010;122(4):334-8.
24. Kiyak YS, Budakoglu, II, Kalaycioglu DB, Kula S, Coskun O. Can preclinical students improve their clinical reasoning skills only by taking case-based online testlets? A randomized controlled study. Innovations in Education and Teaching International. 2022.
25. Kiesewetter J, Sailer M, Jung VM, Schönberger R, Bauer E, Zottmann JM, et al. Learning clinical reasoning: how virtual patient case format and prior knowledge interact. BMC Medical Education. 2020;20(1):1-10.
26. Klein M, Otto B, Fischer MR, Stark R. Fostering medical students' clinical reasoning by learning from errors in clinical case vignettes: effects and conditions of additional prompting procedures to foster self-explanations. Advances in Health Sciences Education. 2019;24(2):331-51.
27. Kuhn J, Mamede S, van den Berg P, Zwaan L, Elshout G, Bindels P, et al. Teaching medical students to apply deliberate reflection. Medical teacher. 2023;46(1):65-72.
28. Lee A, Joynt GM, Lee AK, Ho AM, Groves M, Vlantis AC, et al. Using illness scripts to teach clinical reasoning skills to medical students. Family medicine. 2010;42(4):255-61.
29. Linsen A, Elshout G, Pols D, Zwaan L, Mamede S. Education in clinical reasoning: an experimental study on strategies to foster novice medical students' engagement in learning activities. Health Professions Education. 2018;4(2):86-96.
30. Ludwig S, Schuelper N, Brown J, Anders S, Raupach T. How can we teach medical students to choose wisely? A randomised controlled cross-over study of video- versus text-based case scenarios. BMC medicine. 2018;16(1):107.
31. Mamede S, van Gog T, Moura AS, de Faria RM, Peixoto JM, Rikers RM, et al. Reflection as a strategy to foster medical students' acquisition of diagnostic competence. Medical education. 2012;46(5):464-72.
32. Mamede S, van Gog T, Sampaio AM, de Faria RM, Maria JP, Schmidt HG. How can students' diagnostic competence benefit most from practice with clinical cases? The effects of structured reflection on future diagnosis of the same and novel diseases. Academic medicine : journal of the Association of American Medical Colleges. 2014;89(1):121-7.
33. Mamede S, Figueiredo-Soares T, Elói Santos SM, de Faria RMD, Schmidt HG, van Gog T. Fostering novice students' diagnostic ability: the value of guiding deliberate reflection. Medical education. 2019;53(6):628-37.
34. Matinpour M, Sedighi I, Monajemi A, Jafari F, Momtaz HE, Ali Seif Rabiei M. Clinical reasoning and improvement in the quality of medical education. Shiraz E Medical Journal. 2014;15(4):1-4.

35. Middeke A, Anders S, Schuelper M, Raupach T, Schuelper N. Training of clinical reasoning with a Serious Game versus small-group problem-based learning: A prospective study. PloS one. 2018;13(9):e0203851.
36. Mlika M, Dziri C, Jallouli M, Cheikhrouhou S, Mezni F. Teaching clinical reasoning among undergraduate medical. Journal of Medical Education Development. 2023;16(51):57-64.
37. Moghadami M, Amini M, Moghadami M, Dalal B, Charlin B. Teaching clinical reasoning to undergraduate medical students by illness script method: a randomized controlled trial. BMC medical education. 2021;21(1):87.
38. Mutter MK, Martindale JR, Shah N, Gusic ME, Wolf SJ. Case-Based Teaching: Does the Addition of High-Fidelity Simulation Make a Difference in Medical Students' Clinical Reasoning Skills? Medical science educator. 2020;30(1):307-13.
39. Oliveira JCV, Peixoto AB, Marinho GEM, Peixoto JM. Teaching of Clinical Reasoning Guided by Illness Script Theory. Arquivos Brasileiros de Cardiologia. 2022;119(5):14-21.
40. Ong KY, Ng CWQ, Tan NCK, Tan K. Differential effects of team-based learning on clinical reasoning. The clinical teacher. 2022;19(1):17-23.
41. Peahl AF, Tarr EE, Has P, Hampton BS. Impact of 4 Components of Instructional Design Video on Medical Student Medical Decision Making During the Inpatient Rounding Experience. Journal of surgical education. 2019;76(5):1286-92.
42. Peixoto JM, Mamede S, de Faria RMD, Moura AS, Santos SME, Schmidt HG. The effect of self-explanation of pathophysiological mechanisms of diseases on medical students' diagnostic performance. Advances in Health Sciences Education. 2017;22(5):1183-97.
43. Raupach T, Andresen JC, Meyer K, Strobel L, Koziolek M, Jung W, et al. Test-enhanced learning of clinical reasoning: a crossover randomised trial. Medical education. 2016;50(7):711-20.
44. Ribeiro LMC, Mamede S, de Brito EM, Moura AS, de Faria RMD, Schmidt HG. Effects of deliberate reflection on students' engagement in learning and learning outcomes. Medical education. 2019;53(4):390-7.
45. Schubach F, Goos M, Fabry G, Vach W, Boeker M. Virtual patients in the acquisition of clinical reasoning skills: does presentation mode matter? A quasi-randomized controlled trial. BMC medical education. 2017;17(1):165.
46. Schuelper N, Ludwig S, Anders S, Raupach T. The Impact of Medical Students' Individual Teaching Format Choice on the Learning Outcome Related to Clinical Reasoning. JMIR medical education. 2019;5(2):e13386.
47. Si J, Kong HH, Lee SH. Developing Clinical Reasoning Skills Through Argumentation With the Concept Map Method in Medical Problem-Based Learning. Interdisciplinary Journal of Problem-Based Learning. 2019;13(1).
48. Sobocan M, Turk N, Dinevski D, Hojs R, Balon BP. Problem-based learning in internal medicine: virtual patients or paper-based problems? Internal Medicine Journal. 2017;47(1):99-103.
49. Stark R, Kopp V, Fischer MR. Case-based learning with worked examples in complex domains: Two experimental studies in undergraduate medical education. Learning and instruction. 2011;21(1):22-33.
50. Stein GH, Tokunaga H, Ando H, Obika M, Miyoshi T, Tokuda Y, et al. Clinical Reasoning Web-based Prototypic Module for Tutors Teaching 5th Grade Medical Students : A Pilot Randomized Study. Journal of General and Family Medicine. 2015;16(1):13-25.
51. Stieger S, Praschinger A, Kletter K, Kainberger F. Diagnostic grand rounds: a new teaching concept to train diagnostic reasoning. European journal of radiology. 2011;78(3):349-52.
52. Weidenbusch M, Lenzer B, Sailer M, Strobel C, Kunisch R, Kiesewetter J, et al. Can clinical case discussions foster clinical reasoning skills in undergraduate medical education? A randomised controlled trial. BMJ open. 2019;9(9):e025973.

53. Xu G, Zhao L, Zhou M. Effectiveness of problem-based learning combined with lecture based learning methodology in renal pathology education. Cogent Education. 2023;10(1).
54. Yousefichaijan P, Jafari F, Kahbazi M, Rafiei M, Pakniyat A. The effect of short-term workshop on improving clinical reasoning skill of medical students. Medical journal of the Islamic Republic of Iran. 2016;30:396.
