# Supplementary material for: Teaching and learning clinical reasoning skill in undergraduate medical students: A scoping review
Source: PLoS One. 2024 Oct 16;19(10):e0309606. doi: 10.1371/journal.pone.0309606 (PMC11482728; doi:10.1371/journal.pone.0309606)
Supplement: S1 File — (PDF) [file pone.0309606.s014.pdf]

## Search Syntax

*This term used for search:*

“Instruction”, “Teaching", “Clinical Education", “Teaching Hospital", “Clinical Reasoning”, “Diagnostic Reasoning", “Medical Decision”, “Medical Decision Making", “Clinical Decision Making", “Differential Diagnosis", “Diagnostic Accuracy".

### Search syntax in PubMed:(23 March 2024:13148)

(Instruction[tiab] OR (Clerkship[tiab] AND clinical[tiab]) OR (clinical[tiab] AND teaching[tiab]) OR (clinical[tiab] AND education[tiab]) OR (Hospital*[tiab] AND teaching[tiab]) OR teach*[tiab] OR (Technique*[tiab] AND Training[tiab]) OR (Technic*[tiab] AND Training[tiab]) OR Pedagog*[tiab] OR (Method [tiab] AND Teaching[tiab]) OR (Training[tiab] AND Academic[tiab]) OR (Activit*[tiab] AND Training[tiab]) OR (Technic*[tiab] AND Educational[tiab]) OR (Technique*[tiab] AND Educational[tiab])) AND (“Clinical reasoning”[tiab] OR “Diagnostic reasoning”[tiab] OR “Effect-to-cause reasoning”[tiab] OR “medical decision”[tiab] OR (Medical[tiab] AND “decision making”[tiab]) OR “clinical approach”[tiab] OR “Clinical decision making”[tiab] OR (Decision-Making[tiab] AND Clinical[tiab]) OR (Decision-Making[tiab] AND Medical[tiab]) OR (Diagnos*[tiab] AND differential[tiab]) OR “diagnostic possibility”[tiab] OR (Accuracy[tiab] AND diagnostic[tiab]) OR “diagnosis accuracy”[tiab] OR (clinical[tiab] AND judgment*[tiab])) AND 2010/01/01:2024/03/23[dp]

### Search syntax in Scopus (24 March 2024: 16271)

TITLE-ABS(Instruction OR (Clerkship AND clinical) OR (clinical AND teaching) OR (clinical AND education) OR (Hospital* AND teaching) OR teach* OR (Technique* AND Training) OR (Technic* AND Training) OR Pedagog* OR (Method AND Teaching) OR (Training AND Academic) OR (Activit* AND Training) OR (Technic* AND Educational) OR (Technique* AND Educational)) AND TITLE-ABS(“Clinical reasoning” OR “Diagnostic reasoning” OR “Effect-to-cause reasoning” OR “medical decision” OR (Medical AND “decision making”) OR “clinical approach” OR “Clinical decision making” OR (Decision-Making AND Clinical) OR (Decision-Making AND Medical) OR

(Diagnos* AND differential) OR “diagnostic possibility” OR (Accuracy AND diagnostic) OR “diagnosis accuracy” OR (clinical AND judgment*)) AND (PUBYEAR > 2009 AND PUBYEAR < 2025)

### Search syntax in Web of Sciences (24 March 2024: 17093)

(TS=Instruction OR (TS=(Clerkship) AND TS=(clinical)) OR (TS=(clinical) AND TS=(teaching)) OR (TS=(clinical) AND TS=(education)) OR (TS=(Hospital*) AND TS=(teaching)) OR TS=(teach*) OR (TS=(Technique*) AND TS=(Training)) OR (TS=(Technic*) AND TS=(Training)) OR TS=(Pedagog*) OR (TS=(Method) AND TS=(Teaching)) OR (TS=(Training) AND TS=(Academic)) OR (TS=(Activit*) AND TS=(Training)) OR (TS=(Technic*) AND TS=(Educational)) OR (TS=(Technique*) AND TS=(Educational))) AND (TS=(“Clinical reasoning”) OR TS=(“Diagnostic reasoning”) OR TS=(“Effect-to-cause reasoning”) OR (TS=“medical decision”) OR (TS=(Medical) AND TS=(“decision making”)) OR TS=(“clinical approach”) OR TS=(“Clinical decision making”) OR (TS=(Decision-Making) AND TS=(Clinical)) OR (TS=(Decision-Making) AND TS=(Medical)) OR (TS=(Diagnos*) AND TS=(differential)) OR TS=(“diagnostic possibility”) OR (TS=(Accuracy) AND TS=(diagnostic)) OR TS=(“diagnosis accuracy”) OR (TS=(clinical) AND TS=(judgment*)))

*Indexes=SCI-EXPANDED, SSCI, A&HCI, CPCI-S, CPCI-SSH, BKCI-S, BKCI-SSH, ESCI, CCR-EXPANDED, IC Timespan=2010-2024*

### Search syntax in Eric (24March 2024: 69)

((title:(instruction) OR abstract:(instruction) OR title:(teach OR teaching OR teacher) AND title:(clinical OR hospital OR method)) OR (abstract:(teach OR teaching OR teacher) AND abstract:(clinical OR hospital OR method)) OR (title:(train OR training OR trainer) AND title:(technic OR technique OR academic OR activity)) OR (abstract:(train OR training OR trainer) AND abstract:(technic OR technique OR academic OR activity)) OR (title:(clerkship clinical OR clinical education OR Pedagog OR technic educational OR technique educational OR teach OR teaching OR teacher) OR (abstract:(clerkship clinical OR clinical education OR Pedagog OR technic educational OR technique educational OR teach OR teaching OR teacher)) AND

((title:(reason OR reasoning) AND title:(clinical OR diagnosis OR diagnoses OR diagnostic OR “effect to cause”)) OR (abstract:(reason OR reasoning) AND abstract:(clinical OR diagnosis OR diagnoses OR diagnostic OR “effect to cause”)) OR (title:(medical OR clinical) AND title:(decision making)) OR (abstract:(medical OR clinical) AND abstract:(decision making)) OR (title:(diagnosis OR diagnoses OR diagnostic) AND title:(differential OR possibility OR accuracy)) OR (abstract:(diagnosis OR diagnoses OR diagnostic) AND abstract:(differential OR possibility OR accuracy)) OR title:(“clinical approach” OR “clinical judgment”)) AND pubyear:2009-2025
