# Supplementary material for: Teaching and learning clinical reasoning skill in undergraduate medical students: A scoping review
Source: PLoS One. 2024 Oct 16;19(10):e0309606. doi: 10.1371/journal.pone.0309606 (PMC11482728; doi:10.1371/journal.pone.0309606)
Supplement: S2 File — (PDF) [file pone.0309606.s015.pdf]

List of excluded articles with reasons.

#	Author(s)	Article title	Reason for exclusion
1	Abdul-Kadir (2022)	Enriching traditional didactic teaching in undergraduate ophthalmology with lateral thinking method: a prospective study	Other /unclear type of test
2	Abouzeid (2023)	Teaching by concordance: Individual versus team-based performance	No full text
3	Ahopelto (2011)	A follow-up study of medical students' biomedical understanding and clinical reasoning concerning the cardiovascular system	More than one reason (Other/ unclear study design +other/unclear type of test)
4	Altshuler (2014)	Development of a bedside teaching service to enhance physical examination and clinical reasoning skills	No full text
5	Anderson (2022)	Integrating Clinical Reasoning Skills in a Pre-professional Undergraduate Human Anatomy Course	Unclear / other participants
6	Aniort (2024)	Impact of reference panel composition on scores of script concordance test assessing basic nephrology knowledge in undergraduate medical education	No full text
7	Asad (2015)	Effectiveness of problem based learning as a strategy to foster problem solving and critical reasoning skills among medical students	Other/ unclear study design
8	Artino Jr (2014)	Exploring clinical reasoning in novices: a self-regulated learning microanalytic assessment approach	More than one reason (Other/ unclear study design +other/unclear type of test)
9	Audétat (2010)	Supervision of clinical reasoning Methods and tools to support and promote effective clinical reasoning	Other/ unclear study design
10	Audétat (2017)	Diagnosis and management of clinical reasoning difficulties: Part I. Clinical reasoning supervision and educational diagnosis	Other/ unclear study design
11	Augustin (2022)	The use of podcasts as a tool to teach clinical reasoning: a pseudorandomized and controlled study	No full text
12	Babenko (2022)	In-Person or Online? The Effect of Delivery Mode on Team-Based Learning of Clinical Reasoning in a Family Medicine Clerkship	Other/ unclear study design
13	Bagourd (2015)	Diagnostic sceneries, department of family medicine contribution to clinical reasoning	No full text
14	Bansal (2020)	Developing Medical Students' Broad Clinical Diagnostic Reasoning Through GP-Facilitated Teaching in Hospital Placements	More than one reason (other/unclear test & study design)
15	Bateman (2012)	Virtual patients can be used to teach clinical reasoning	No full text
16	Battista (2018)	Clinical Reasoning in the Primary Care Setting: Two Scenario-Based Simulations for Residents and Attendings	More than one reason (other/unclear test + study design)
17	Beeler (2023)	The effects of procedural and conceptual knowledge on visual learning	Other /unclear type of test

18	Berens (2022)	Effects of Elaboration and Instructor Feedback on Retention of Clinical Reasoning Competence Among Undergraduate Medical Students: A Randomized Crossover Trial	More than one reason (other/unclear test + study publishing format[letter])
19	Berrocal (2018)	Dehydration: A Multidisciplinary Case-Based Discussion for First-Year Medical Students	More than one reason (other/unclear test & study design)
20	Bhusnurmath (2021)	Helping medical students to learn pathology more effectively	More than one reason (other/unclear test & study design)
21	Blissett (2012)	Should we teach using schemas? Evidence from a randomised trial	Another/unclear type of test
22	Blissett (2015)	ECG rhythm analysis with expert and learner-generated schemas in novice learners	Other/unclear type of test
23	Bonifacino (2019)	Think like a doctor: an innovative clinical reasoning curriculum for clerkship-level medical students	No full text
24	Bonifacino (2018)	An innovative skill-based clinical reasoning curriculum for clerkship-level medical students	No full text
25	Braun (2019)	Scaffolding clinical reasoning of medical students with virtual patients: effects on diagnostic accuracy, efficiency, and errors	Other/unclear type of test
26	Brydges (2013)	Divergence in student and educator conceptual structures during auscultation training	Other/unclear type of test
27	Buaprasert (2021)	Diagnostic Accuracy of Extended Focused Assessment with Sonography for Trauma Performed by Paramedic Students: A Simulation-Based Pilot Study	Other/unclear participants
28	Calzada (2020)	Case discussion in teams as a strategy for practicing clinical reasoning since the basic cycle	No English full text
29	Capaldi (2015)	The clinical integrative puzzle for teaching and assessing clinical reasoning: preliminary feasibility, reliability, and validity evidence	Clinical reasoning assessment
30	Chandrasekar (2018)	Promoting student case creation to enhance instruction of clinical reasoning skills: a pilot feasibility study	More than one reason (other/unclear test & study design)
31	Charlin (2012)	Clinical reasoning processes: unravelling complexity through graphical representation	Other/unclear study design
32	Cogan (2020)	How to formalize the supervision of learning of clinical reasoning	No English full text
33	Connors (2015)	Clinical Reasoning and Risk in the Intensive Care Unit	Other/unclear study design
34	Chadha (2021)	A Virtual Clinical Reasoning Case for Medical Students Using an Ophthalmology Model: A Case of Red Eye	More than one reason (other/unclear test & study design)
35	Chamberland (2015)	Self-explanation, an instructional strategy to foster clinical reasoning in medical students	Other/unclear study design
36	Chamberland (2023)	Exploring medical students' use of principles of self-explanation and structured reflection during clerkship	Other/unclear study design

37	Chang (2023)	Introducing second-year medical students to diagnostic reasoning concepts and skills via a virtual curriculum	Other/unclear study design
38	Cheng (2020)	Artificial intelligence-based education assists medical students' interpretation of hip fracture	More than one reason (other/unclear type of test + other outcomes)
39	Cheng (2023)	Using Clinical Data Visualizations in Electronic Health Record User Interfaces to Enhance Medical Student Diagnostic Reasoning: Randomized Experiment	More than one reason (other / unclear test & study design)
40	Cohen (2023)	Bacterial Meningitis with Cerebral Edema in a Young Adult: A Simulation Case for Medical Students	More than one reason (other / unclear test & study design)
41	Choi (2023)	Using an experiential learning model to teach clinical reasoning theory and cognitive bias: an evaluation of a first-year medical student curriculum	Other/unclear study design
42	Conn (2012)	Clinical teaching and learning: from theory and research to application	Other/unclear study design
43	Connor (2020)	Clinical Reasoning as a Core Competency	Other/unclear study design
44	Consorti (2023)	The challenge of clinical reasoning in chronic multimorbidity: time and interactions in the Health Issues Network model	Other/unclear study design
45	Corrao (2022)	Rethinking clinical decision-making to improve clinical reasoning	Other/unclear study design
46	Chon (2019)	Serious Games in Surgical Medical Education: A Virtual Emergency Department as a Tool for Teaching Clinical Reasoning to Medical Students	Other/unclear type of test
47	Chou (2017)	Application of visually based, computerised diagnostic decision support system in dermatological medical education: a pilot study	More than one reason (other / unclear test & study design)
48	Clarke (2020)	Beginning teachers' developing clinical judgement: knowledge, skills and attributes for clinical teaching	Other participants
49	Collard (2016)	Context impact of clinical scenario on knowledge transfer and reasoning capacity in a medical problem-based learning curriculum	Clinical reasoning assessment
50	Cutrer (2013)	Educational Strategies for Improving Clinical Reasoning	Other/unclear study design
51	Czeskleba (2020)	Clinical reasoning for acute dyspnoea: comparison between final-year medical students from discipline- and competency based undergraduate programmes	Other/unclear study design
52	Da (2018)	Application of mini-clinical evaluation exercise for assessing the integrated-based learning during physical diagnostic course	More than one reason (other/unclear type of test & participants)
53	Darwish (2023)	The Effect of a Structured Pre-Briefing Simulation Session on Medical Students' Competency	More than one reason (other / unclear type of test & other outcomes)
54	Davari (2021)	Problem-based learning as an effective method for teaching theoretical surgery courses to medical students	Other / unclear type of test
55	Dekhtyar (2022)	Use of a structured approach and virtual simulation practice to improve diagnostic reasoning	No full text

56	Delany (2020)	Starting from a higher place: linking Habermas to teaching and learning clinical reasoning in the emergency medicine context	More than one reason (other participants + other outcomes + other/unclear test & study design)
57	Diemers (2015)	Diagnostic reasoning and underlying knowledge of students with preclinical patient contacts in PBL	Other/unclear type of test
58	Djermester (2021)	Bedside teaching without bedside - an introduction to clinical reasoning in COVID-19 times	More than one reason (other/unclear study design & test)
59	Dong (2015)	Use of concept maps to promote electrocardiogram diagnosis learning in undergraduate medical students	Other /unclear type of test
60	Duca (2019)	Bridging the Gap Between the Classroom and the Clerkship: A Clinical Reasoning Curriculum for Third-Year Medical Students	More than one reason (other/unclear type of test & study design)
61	Duffy (2023)	An online case-based teaching and assessment program on clinical history-taking skills and reasoning using simulated patients in response to the COVID-19 pandemic	More than one reason (other/unclear type of test & study design)
62	Dupriez (2023)	Is gallbladder PoCUS diagnostic accuracy accessible to medical students after PoCUS training exclusively on healthy volunteers? A pilot randomized control trial	Other /unclear type of test
63	Durán-Pérez (2019)	CARAIPER scheme: A teaching-learning strategy of clinical reasoning	No full text
64	Durning (2012)	Instructional authenticity and clinical reasoning in undergraduate medical education: a 2-year, prospective, randomized trial	Other /unclear type of test
65	Dwyer (2020)	Effects of Biopsychosocial Education on the Clinical Judgments of Medical Students and GP Trainees Regarding Future Risk of Disability in Chronic Lower Back Pain: A Randomized Control Trial	Other /unclear type of test
66	E. Brush Jr (2019)	Effect of Teaching Bayesian Methods Using Learning by Concept vs Learning by Example on Medical Students' Ability to Estimate Probability of a Diagnosis: A Randomized Clinical Trial	Other /unclear type of test
67	Elizondo-Omaña (2010)	Teaching skills to promote clinical reasoning in early basic science courses	other/unclear study design
68	Fagundes (2020)	Case presentation methods: a randomized controlled trial of the one-minute preceptor versus SNAPPS in a controlled setting	Another/unclear type of test
69	Fan (2021)	Exploration of an effective training system for diagnosis of superficial esophageal squamous cell carcinoma with magnifying narrow-band imaging: Prospective research	Other participants
70	Fässler (2022)	Problem-solving in virtual environment simulations prior to direct instruction for differential diagnosis in medical education: An experimental study	Other /unclear type of test
71	Ferradji (2016)	Collaborative environment for remote clinical reasoning learning	no full text

72	Filho (2019)	Effects of deliberate reflection on diagnostic accuracy, confidence and diagnostic calibration in dermatology	Other /unclear type of test
73	Fink (2023)	Diagnosing virtual patients: the interplay between knowledge and diagnostic activities	Another/unclear study design
74	Forbes (2023)	A Team-based Learning Approach During Pediatric Clerkship to Promote Clinical Reasoning	No full text
75	Fukuta (2018)	First-person perspective video to enhance simulation	Other /unclear type of test
76	Furlan (2022)	Learning Analytics Applied to Clinical Diagnostic Reasoning Using a Natural Language Processing-Based Virtual Patient Simulator: Case Study	Other /unclear study design
77	Gamble (2023)	Should Virtual Objective Structured Clinical Examination (OSCE) Teaching Replace or Complement Face-to-Face Teaching in the Post-COVID-19 Educational Environment: An Evaluation of an Innovative National COVID-19 Teaching Programme	More than one reason (Other /unclear type of test & study design + other outcome)
78	Gardiner (2016)	The art of self-knowledge and deduction in clinical practice	Other /unclear study design
79	Gilkes (2022)	Teaching and assessment of clinical diagnostic reasoning in medical students	No full text
80	Goldowsky (2023)	Self-regulated learning and the future of diagnostic reasoning education	Other /unclear study design
81	Goldszmidt (2013)	Developing a unified list of physicians' reasoning tasks during clinical encounters	Other participants
82	Gavinski (2023)	Clinical reasoning curricula in undergraduate medical education: bridging the gap between recommendations and reality	No full text
83	Gehlhar (2014)	Do different medical curricula influence self-assessed clinical thinking of students?	More than one reasons (other /unclear study design + other outcomes)
84	Gómez (2012)	Considerations on the basic biomedical sciencers and the learning of clinic in the curricular improvement of the medical career in Cuba	No English full text
85	Gómez Morales (2022)	Performance in cardiac examination and diagnostic accuracy after training medical students with simulators vs. patients	Other/unclear type of test
86	Goodin (2019)	Developing clinical reasoning skills in teacher candidates using a problem-based learning approach	More than one reason (other outcome & participants)
87	Gouzi (2019)	Interactive whiteboard use in clinical reasoning sessions to teach diagnostic test ordering and interpretation to undergraduate medical students	More than one reason (other /unclear type of test + unclear outcome)
88	Groves (2013)	Analysing clinical reasoning characteristics using a combined methods approach	Clinical reasoning Assessment
89	Gruenberg (2020)	A Randomized, Crossover Pilot Study of a Novel Web-Based/Mobile Platform for Collaborative Small Group Practice in Therapeutic Reasoning	Other participants

90	Hakim (2023)	Integration of respiratory physiology and clinical reasoning in the early years of a medical curriculum: engaging with students in a large classroom setting	More than one reason (other /unclear type of test + other / unclear study design)
91	Harendza (2017)	Implementation of a Clinical Reasoning Course in the Internal Medicine trimester of the final year of undergraduate medical training and its effect on students' case presentation and differential diagnostic skills	More than one reason (other /unclear type of test + other / unclear study design)
92	Harris (2011)	Clinical reasoning sessions: back to the patient	More than one reason (other /unclear type of test + other / unclear study design)
93	Hassan (2022)	The Structural Analysis: Incorporating Structurally Competent Clinical Reasoning into Case-Based Presentations	Other / unclear study design
94	Hege (2018)	How to tell a patient's story? Influence of the case narrative design on the clinical reasoning process in virtual patients	More than one reason (Other /unclear type of test + unclear educational intervention)
95	Hege (2017)	A Clinical Reasoning Tool for Virtual Patients: Design-Based Research Study	other /unclear study design
96	Hege (2023)	Developing a European longitudinal and interprofessional curriculum for clinical reasoning	other /unclear study design
97	Henderson (2015)	Cross cultural teaching of patient care and clinical reasoning in Asia	No full text
98	Hoshina (2021)	Does a learner-centered approach using teleconference improve medical students' psychological safety and self-explanation in clinical reasoning conferences? a crossover study	other/unclear type of test
99	Hu (2023)	Three-Dimensional Multimodality Image Reconstruction as Teaching Tool for Case-based learning among medical postgraduates: a focus on primary pelvic bone Tumour Education	More than one reason (Other participants + other/unclear type of test)
100	Huang (2021)	Exploration of an effective method for the step-by-step presentation of case information to guide grade 4 medical students to develop clinical reasoning skills	More than one reason (unclear type of test & study design)
101	Ilgen (2011)	Adjusting our lens: can developmental differences in diagnostic reasoning be harnessed to improve health professional and trainee assessment?	other /unclear study design
102	Jackson (2022)	Diagnostic Reasoning, Deconstructed: A Teachable Framework for Novices	other /unclear study design
103	Imran (2022)	Team-based learning versus interactive lecture in achieving learning outcomes and improving clinical reasoning skills: a randomized crossover study	other/unclear type of test
104	Isaza-Restrepo (2018)	The virtual patient as a learning tool: a mixed quantitative qualitative study	More than one reason (other/unclear type of test + another outcome)
105	Ishizuka (2023)	Hybrid PBL and Pure PBL: Which one is more effective in developing clinical reasoning skills for general medicine clerkship? -A mixed-method study	Other /unclear type of test

106	Ishizuka (2023)	The influence of Gamification on medical students' diagnostic decision making and awareness of medical cost: a mixed-method study	Other / unclear study design
107	Jacobson (2010)	Integrated Cases Section: a course designed to promote clinical reasoning in year 2 medical students	Other / unclear study design (program evaluation)
108	Jawaid (2019)	Effect of Paper- and Computer-based Simulated Instructions on Clinical Reasoning Skills of Undergraduate Medical Students: A Randomized Control Trial	Other /unclear type of test
109	Jayasinghe (2016)	Describing complex clinical scenarios at the bed-side: Is a systems science approach useful? Exploring a novel diagrammatic approach to facilitate clinical reasoning	Other/unclear study design
110	Jellouli (2020)	Evaluation of Clinical Reasoning Learning for students in SCMS2, pediatrics Module	No full text
111	Johnson (2015)	Are therapist assumptions an enabler or barrier to sound clinical reasoning?	No full text
112	Juvin-Bouvier (2017)	Simulation-based training during medical degree for diagnosis of mitral stenosis. Clinics, images and decision making	No full text
113	Kafke (2023)	Can clinical decision support systems be an asset in medical education? An experimental approach	Using CDSS for increasing diagnostic accuracy (don't use educational strategy)
114	Kämmer (2021)	Differential Diagnosis Checklists Reduce Diagnostic Error Differentially: A Randomized Experiment	Unclear educational strategy
115	Kaminska (2022)	Does Walking Help to Generate a Differential Diagnosis?	Unclear educational intervention)
116	Kandiah (2017)	Clinical reasoning and knowledge management in final year medical students: the role of Student-led Grand Rounds	More than one reason (unclear type of test & study design)
117	Kassianos (2020)	eCREST: a novel online patient simulation resource to aid better diagnosis through developing clinical reasoning	Other/ unclear study design
118	Kazi (2013)	Clinical reasoning gains in medical PBL: an UMLS based tutoring system	Other/ unclear study design
119	Keemink (2018)	Illness script development in pre-clinical education through case-based clinical reasoning training	other/unclear type of test
120	Kelekar (2020)	Evaluation of the effect of a new clinical reasoning curriculum in a pre-clerkship clinical skills course	Other/ unclear study design
121	Kıyak (2024)	Test-Only Learning via Virtual Patients to Improve Surgical Illness Scripts of Preclinical Medical Students as a Preparation for Clinical Clerkships: An Experimental Study	No full text
122	Kiesewetter (2011)	Cognitive Problem Solving Patterns of Medical Students Correlate with Success in Diagnostic Case Solutions	Other/ unclear type of test
123	Kim (2013)	Evaluation of an e-PBL model to promote individual reasoning	More than one reason (unclear type of test & study design)

124	King (2017)	Developing Validity Evidence for the Written Pediatric History and Physical Exam Evaluation Rubric	More than one reason (unclear type of test & study design)
125	Kiran (2016)	Enhancing the clinical reasoning skills of postgraduate students in internal medicine through medical nonfiction and nonmedical fiction extracurricular books	Other participants
126	Kobner (2021)	The Challenging Case Conference: A Gamified Approach to Clinical Reasoning in the Video Conference Era	Other /unclear study design
127	Kok (2015)	Case Comparisons: An Efficient Way of Learning Radiology	Other/unclear type of test
128	Koenemann (2020)	Clinical case discussions – a novel, supervised peer-teaching format to promote clinical reasoning in medical students	More than one reason (unclear type of test & study design)
129	Krupat (2017)	Avoiding premature closure and reaching diagnostic accuracy: some key predictive factors	More than one reason (other/unclear type of test & study design + other outcomes)
130	Kulasegaram (2017)	Contexts, concepts and cognition: principles for the transfer of basic science knowledge	More than one reason (other outcome + other /unclear test)
131	Kumar (2020)	Infusing the axioms of clinical reasoning while designing clinical anatomy case vignettes teaching for novice medical students: A randomised cross over study	Other /unclear type of test
132	Kulatunga-Moruzi (2011)	Teaching post training: influencing diagnostic strategy with instructions at test	More than one reason (Other / unclear study design + unclear educational strategy)
133	Lambe (2018)	Guided Reflection Interventions Show No Effect on Diagnostic Accuracy in Medical Students	More than one reason (Other /unclear type of test + no educational intervention)
134	LaRochelle (2011)	Authenticity of Instruction and Student Performance: A Prospective Randomized Trial	More than one reason (Other /unclear type of test + no pretest and posttest + other study design + OSCE, video, and essay in the end of academic year)
135	LaRochelle (2012)	Impact of increased authenticity in instructional format on preclerkship students' performance: a two-year, prospective, randomized study	More than one reason (Other /unclear type of test + no pretest and posttest + other study design + OSCE, video, and essay in the end of academic year)
136	LaRochelle (2016)	Pre-clerkship clinical skills and clinical reasoning course performance: Explaining the variance in clerkship performance: Pre-clerkship predictors of clerkship variance	More than one reason (Other /unclear type of test + no pretest and posttest + OSCE, video, and essay in the end of academic year)
137	Leeds (2020)	Teaching heuristics and mnemonics to improve generation of differential diagnoses	More than one reason (Other/unclear type of test & study design)
138	Larsen (2023)	Education in Focused Lung Ultrasound Using Gamified Immersive Virtual Reality: A Randomized Controlled Study	More than one reason (other/unclear type of test + other outcomes)

139	Ienghong (2021)	Comparative accuracy of ultrasound and physical examination conducted by clinical year medical students in diagnosing ascites	Other/unclear type of test
140	Levin (2016)	Teaching Clinical Reasoning to Medical Students: A Case-Based Illness Script Worksheet Approach	More than one reason (Other/unclear type of test & study design)
141	Levinson (2017)	Description and student self-evaluation of a pilot integrated small group learning and simulation programme for medical students in the first clinical year	Other/unclear type of test
142	Li (2019)	Orderly display of limb lead ECGs raises Chinese intern's diagnostic accuracy when determining frontal plane QRS axis	Other /unclear type of test
143	Linn (2012)	Clinical reasoning: A guide to improving teaching and practice	No full text
144	Linsenmeyer (2017)	Development of an Educational Activity for First- and Second-Year Medical Students Using Cadaver Pathologies to Enhance Clinical Reasoning and Prepare for Entrustment in Providing Oral Case Presentations	Other study design
145	Lippa (2015)	Assessment of Durability of Online and Multisensory Learning Using an Ophthalmology Model	Other study design
146	Lisk (2016)	Exploring cognitive integration of basic science and its effect on diagnostic reasoning in novices	Other participants
147	Lisk (2017)	Examining the effect of self-explanation on cognitive integration of basic and clinical sciences in novices	Other participants
148	Lockwood (2018)	Practice Improvement Using Virtual Online Training: A Novel App-Based Platform to Teach Clinical Reasoning in Rheumatology	No full text
149	López (2015)	Usefulness of active participatory educational strategy for the development of clinical reasoning in undergraduate education	No full text
150	Lorello (2024)	Relinquishing control? Supervisor co-regulation may disrupt students' self-regulated learning during simulation-based training	No full text
151	Loughland (2022)	A taxonomy of clinical reasoning for pre-service teachers on professional experience	More than one reason (other participants + other / unclear study design)
152	Lupi (2018)	The Educational Effects of a Summative Diagnostic Reasoning Examination among Second-Year Medical Students	Other / unclear study design
153	Ma (2016)	Comparison of student perception and performance between case-based learning and lecture-based learning in a clinical laboratory immunology course	More than one reason (Other/unclear type of test & study design + other outcome)
154	Maicher (2017)	Developing a Conversational Virtual Standardized Patient to Enable Students to Practice History-Taking Skills	Other/unclear study design
155	Makkink (2018)	AIMED AT ITCHEDD: A proof-of-concept study to evaluate a mnemonic-based approach to clinical reasoning in the emergency medical care educational setting	More than one reason (other outcomes + other / unclear study design)

156	Mallory (2023)	Teaching Clinical Reasoning in the Preclinical Period	Other / unclear study design
157	Malterud (2019)	Diagnostic knowing in general practice: interpretative action and reflexivity	Other / unclear study design
158	Mamede (2023)	Deliberate reflection and clinical reasoning: Founding ideas and empirical findings	Other / unclear study design
159	Marne (2016)	Evaluation of an E-learning resource on approach to the first unprovoked seizure	More than one reason (unclear educational intervention + other/unclear type of test)
160	Mazigh (2023)	Case based self-directed learning tool versus clinical reasoning learning sessions	No full text
161	McBee (2017)	Contextual factors and clinical reasoning: differences in diagnostic and therapeutic reasoning in board certified versus resident physicians	More than one reason (other/unclear study design + other participants)
162	McGervey (2020)	Getting bang for your buck in clinical reasoning: innovative peer-to-peer teaching utilizing feedback loops and metacognition	No full text
163	Medvecky (2017)	Teaching clinical reasoning: Turning novices into expert diagnosticians outline and applications to teaching clinical orthopedics	No full text
164	Meral Savran (2015)	Are Medical Students Being Taught Anatomy in a Way That Best Prepares Them to Be a Physician?	More than one reason (other/unclear type of test & study design)
165	Middeke (2020)	Transfer of Clinical Reasoning Trained with a Serious Game to Comparable Clinical Problems: A Prospective Randomized Study	other/unclear type of test (unclear having / not having pretest, posttest, and follow up)
166	Ming-Chen (2017)	Analyzing the effectiveness of teaching and factors in clinical decision-making	other/unclear type of test
167	Modi (2015)	Teaching and Assessing Clinical Reasoning Skills	Other / unclear study design
168	Montaldo (2013)	Teaching of clinical reasoning to medical students using prototypical clinical cases	No English full text
169	Montastruc (2024)	Teaching pharmacovigilance to French medical students during the COVID-19 pandemic: Interest of distance learning clinical reasoning sessions	No full text
170	Mookherjee (2011)	Bedside teaching of clinical reasoning and evidence-based physical examination	No full text
171	Moonen (2010)	Diagnostic exercise for the medical school student: teaching diagnostic reasoning	No full text
172	Montgomery (2023)	Teaching delirium to undergraduate medical students: Exploring the effects of a cross-professional group objective structured clinical examination compared to standard education	Other /unclear type of test
173	Morgan (2024)	Game-based learning to improve diagnostic accuracy: a pilot randomized-controlled trial	More than one reason (other /unclear type of test + combination of different participants)
174	Mousseau (2021)	E-learning to teach medical students about acute otitis media: A randomized controlled trial	Other /unclear type of test

175	Muñoz-Sandoval (2015)	Proposal to improve the teaching of clinical reasoning	No full text
176	Myung (2013)	Effect of enhanced analytic reasoning on diagnostic accuracy: a randomized controlled study	Other /unclear type of test
177	N. Covin (2019)	Convergent Validity of a Novel Clinical Reasoning Tool in Clerkship Medical Students: Pilot Study	Other / unclear study design
178	Nadler (2016)	Applying Judgment Analysis Theory and Methods to Obtain an Insight into Clinical Judgments: Implementation and Findings with a Simulated Neonatal Intensive Care Unit Setup	Other participants
179	Nakada (2018)	Clinical Reasoning in Kampo Education for Teaching Kampo Beginners	Other participants
180	Nendaz (2011)	Bringing explicit insight into cognitive psychology features during clinical reasoning seminars: a prospective, controlled study	other/unclear type of test (unclear having/not having pretest, posttest, and fallow up)
181	Nogueira (2018)	Caries treatment decisions among undergraduate and postgraduate students supported by visual detection systems	Other participants
182	Nolt (2018)	Design and delivery of a new clinical reasoning course	More than one reason (other/unclear type of test & study design)
183	Olszynski (2020)	the Clinical Ultrasonography Elective in Clerkship (CUSEC): A pilot elective for senior clerkship students at the University of Saskatchewan	other / unclear study design
184	Papa (2016)	A Dual Processing Theory Based Approach to Instruction and Assessment of Diagnostic Competencies	other / unclear study design (Clinical reasoning theory)
185	Parks (2015)	Effect of a point-of-care ultrasound protocol on the diagnostic performance of medical learners during simulated cardiorespiratory scenarios	other / unclear study design
186	Payne (2011)	Effect of a metacognitive intervention on cognitive heuristic use during diagnostic reasoning	other / unclear study design (thesis)
187	Paul (2023)	Comparative effectiveness study of flipped classroom versus online-only instruction of clinical reasoning for medical students	other/unclear type of test
188	Peacock (2015)	Patient exposure in the basic science classroom enhances differential diagnosis formation and clinical decision-making	other / unclear study design
189	Penner (2023)	Reasoning on Rounds: a Framework for Teaching Diagnostic Reasoning in the Inpatient Setting	other / unclear study design
190	Petersen (2023)	Online Virtual Patient Cases vs. Weekly Classroom Lectures in an Internal Medicine Clerkship: Effects on Military Learner Outcomes	other / unclear study design
191	Pincavage (2020)	Virtual patient simulation to teach clinical reasoning and reduce diagnostic error	No full text
192	Pinnock (2012)	evPaeds: undergraduate clinical reasoning	other / unclear study design

193	Pisano (2020)	The Bloody Board Game: A Game-Based Approach for Learning High-Value Care Principles in the Setting of Anemia Diagnosis	Other participants
194	Plackett (2020)	Online patient simulation training to improve clinical reasoning: a feasibility randomised controlled trial	Other/unclear type of test
195	Punj (2014)	Palpation as a useful diagnostic tool for skin lesions	More than one reason (other / unclear type of test & study design)
196	Quiroga-Garza (2020)	The Use of Clinical Reasoning Skills in the Setting of Uncertainty: A Case of Trial Femoral Head Migration	Other / unclear study design
197	Quispe-Cardenas (2021)	Teaching clinical reasoning in times of pandemics	Other / unclear study design
198	Radkowitsch (2021)	Learning to diagnose collaboratively–Effects of adaptive collaboration scripts in agent-based medical simulations	Other / unclear type of test
199	Recker (2022)	Application of test-enhanced learning (TEL) in obstetrics and gynecology: a prospective study	other/unclear type of test
200	Rencic (2017)	Clinical reasoning education at US medical schools: results from a national survey of internal medicine clerkship directors	Other / unclear study design
201	Roberti (2015)	Development of clinical reasoning in an undergraduate medical program at a Brazilian university	Other study design
202	Rowat (2022)	Longitudinal clinical reasoning theme embedded across four years of a medical school curriculum	No full text
203	Ruina (2023)	Application of Ultrasonography Visualization Teaching in the Integration Course of Diagnostics and Ultrasonic Medicine for Eight-year Clinical Medicine Program Students	No English full text
204	Saeed (2023)	Hybridizing video-based learning with simulation for flipping the clinical skills learning at a university hospital in Pakistan	Other /unclear type of test
205	Salava (2022)	Perceptual learning modules in undergraduate dermatology teaching	More than one reason (other / unclear type of test & study design)
206	Salava (2023)	Perceptual learning in dermatology-A Finnish cohort study of undergraduate medical students	More than one reason (other / unclear type of test & study design)
207	Santhosh (2019)	Diagnostic uncertainty: from education to communication	other / unclear study design
208	Sawanyawisuth (2015)	Expressing clinical reasoning and uncertainties during a Thai internal medicine ambulatory care rotation: does the SNAPPS technique generalize?	Other/unclear type of test
209	Seneviratne (2014)	Video-based training improves the accuracy of seizure diagnosis	More than one reason (Other /unclear type of test + other outcome)
210	Shah (2016)	Online self-study of chest X-rays shows no difference between blocked and mixed practice	Other /unclear type of test

211	Seki (2016)	How do case presentation teaching methods affect learning outcomes? - SNAPPS and the One-Minute preceptor	Other participants
212	Sheldon (2023)	Learning strategy impacts medical diagnostic reasoning in early learners	Other participants
213	Sherbino (2014)	Ineffectiveness of cognitive forcing strategies to reduce biases in diagnostic reasoning: a controlled trial	More than one reason (other outcome + other/unclear type of test)
214	Shikino (2015)	Influence of predicting the diagnosis from history on the accuracy of physical examination	Other /unclear type of test
215	Shimizu (2013)	Effects of the use of differential diagnosis checklist and general de-biasing checklist on diagnostic performance in comparison to intuitive diagnosis	Other / unclear study design
216	Schmidt (2024)	An early-curricular team learning activity to foster integration of biochemical concepts and clinical sciences in undergraduate medical education	No full text
217	Scott (2021)	Developing critical thinking skills for delivering optimal care	Other study design
218	Seif (2014)	The development of clinical reasoning and interprofessional behaviors: service-learning at a student-run free clinic	More than one reason (Other/unclear type of test + combination of PT, OT, PA, medical and pharmacy students)
219	Si (2022)	Application of Flipped Classroom Combined with Case-based Collaborative Learning in Medical Teaching in the Experimental Eight-year Medical Doctor Program	No English full text
220	Sibbald (2013)	Cardiac examination and the effect of dual-processing instruction in a cardiopulmonary simulator	unclear type of educational strategy
221	Smith (2016)	The Development and Preliminary Validation of a Rubric to Assess Medical Students' Written Summary Statements in Virtual Patient Cases	Other / unclear study design
222	Spahic (2023)	Promoting clinical reasoning with meta-memory techniques to teach broad differential diagnosis generation in a pediatric core clerkship	No full text
223	Steinmetz (2018)	Accuracy of Medical Students in Detecting Pleural Effusion Using Lung Ultrasound as an Adjuncttothe Physical Examination	Other / unclear study design
224	Staal (2022)	Does a suggested diagnosis in a general practitioners' referral question impact diagnostic reasoning: an experimental study	Unclear type of educational strategy
225	Staal (2024)	Impact of performance and information feedback on medical interns' confidence-accuracy calibration	Other /unclear type of test
226	Stout (2019)	Standardising online clinical reasoning teaching for undergraduate students in Somaliland	No full text
227	Strowd (2015)	A Guide to Developing Clinical Reasoning Skills in Neurology: A Focus on Medical Students	No full text

228	Sullivan (2017)	The Clinical Reasoning Conference: A Flipped-Classroom, Case-Based Approach to Teaching and Learning in the Pre-Clerkship Medical Curriculum	No full text
229	Swartz (2014)	A clinical refresher course for medical scientist trainees	More than one reason (other / unclear study design + other outcomes)
230	Tayce (2022)	The Use of a Modified Script Concordance Test in Clinical Rounds to Foster and Assess Clinical Reasoning Skills	More than one reason (other/unclear participants + Other / unclear study design)
231	Tayyeb (2013)	Effectiveness of problem based learning as an instructional tool for acquisition of content knowledge and promotion of critical thinking among medical students	More than one reason (Other /unclear type of test + other outcomes)
232	Thach (2020)	Worked examples for teaching electrocardiogram interpretation: Salient or discriminatory features?	Other /unclear type of test
233	Toledo (2021)	Abdominal ultrasound augments the medical students' ability to identify free intraabdominal fluid	More than one reason (Other / unclear type of test & study design)
234	Torre (2019)	The Clinical Reasoning Mapping Exercise (CResME): a new tool for exploring clinical reasoning	Other / unclear study design
235	Trinidad (2023)	Excellence via Strategic Experiential Learning (ExSEL): A Continuous Improvement Project for Developing Clinical Reasoning and Management	No full text
236	Tschandl (2015)	Teaching dermatoscopy of pigmented skin tumours to novices: comparison of analytic vs. heuristic approach	Comparing two diagnosis approach
237	Valentine (2022)	Problem representation and diagnostic accuracy during an OSCE	No full text
238	Van Es (2016)	Cytopathology whole slide images and adaptive tutorials for senior medical students: a randomized crossover trial	More than one reason (other/unclear type of test + unclear educational strategy)
239	Udrea (2017)	Effects of Student-Performed Point-of-Care Ultrasound on Physician Diagnosis and Management of Patients in the Emergency Department	Other / unclear study design
240	Watari (2020)	The Utility of Virtual Patient Simulations for Clinical Reasoning Education	Other /unclear type of test
241	Watters (2014)	Diagnostic conversations: Clinical Decision Making in surgery - Part 1	Other/ unclear study design
242	Watters (2014)	Diagnostic conversations: Clinical Decision Making in surgery - Part 2	Other/ unclear study design
243	Wells (2015)	Clinical thinking in psychiatry	Other/ unclear study design
244	Williams (2022)	Using the Unfolding Case Study to Improve Clinical Reasoning	More than one reason (other/ unclear study design + other participants)
245	Wu (2014)	Improving the learning of clinical reasoning through computer-based cognitive representation	More than one reason (other/unclear type of test & study design)
246	Wu (2016)	Visualizing complex processes using a cognitive-mapping tool to support the learning of clinical reasoning	More than one reason (other/unclear type of test & study design)
247	Wu (2017)	Evaluation of a Web-Based Module and an Otoscopy Simulator in Teaching Ear Disease	Other /unclear type of test

248	Yanagita (2023)	Improving diagnostic accuracy using a clinical decision support system for medical students during history-taking: a randomized clinical trial	Don't using teaching and learning strategy and assessing impact of technology on diagnostic accuracy
249	Yang (2023)	Cognitive and motor skill competence are different: Results from a prospective randomized trial using virtual reality simulator and educational video in laparoscopic cholecystectomy	No full text
250	Yuan (2020)	Investigating the Role of Cognitive Feedback in Practice-Oriented Learning for Clinical Diagnostics	Other/ unclear type of test
251	Zagury-Orly (2022)	The Student-Generated Reasoning Tool (SGRT): Linking medical knowledge and clinical reasoning in preclinical education	More than one reason (other/unclear type of test & study design)
252	Zapata-Ospina (2021)	Clinical reasoning in medicine II: Towards an integrating definition	No full text
253	Zhao (2023)	Comparison of case-based learning using Watson for oncology and traditional method in teaching undergraduate medical students	No full text
