# Supplementary material for: Teaching and learning clinical reasoning skill in undergraduate medical students: A scoping review
Source: PLoS One. 2024 Oct 16;19(10):e0309606. doi: 10.1371/journal.pone.0309606 (PMC11482728; doi:10.1371/journal.pone.0309606)
Supplement: S3 File — (PDF) [file pone.0309606.s016.pdf]

Abbreviations

AAA: **A**bdominal **A**ortic **A**neurysm

AD: **A**ortic **D**issection

AF: **A**trial **F**ibrillation

AMS: **A**ltered **M**ental **S**tatus

CAD: **C**oronary **A**rtery **D**isease

CASUS: Virtual Patient E-Learning and Online Education Software

CCD: **C**linical **C**ase **D**iscussion

CCS: **C**omputer-based **C**ase **S**imulations

CHB: **C**omplete **H**eart **B**lock

CHF: **C**ongestive **H**eart **F**ailure

COPD: **C**hronic **O**bstructive **P**ulmonary **D**isease

CRP: **C**linical **R**easoning **P**roblem

CS-M: **C**ase **S**ession **M**anikin

CS-NM: **C**ase **S**ession **N**o **M**anikin

DDXC: **D**ifferential **D**iagnosis **C**hecklist

DTI: **D**iagnostic **T**hinking **I**nventory

EMERGE: Serious Game, the virtual Accident & Emergency department

EMQ: **E**xtended **M**atching **Q**uestions

GER: **G**astroesophageal **R**eflux

GPA: **G**rade **P**oint **A**verage

HTN: hypertension

HZ: **H**erpes **Z**oster

ICR: **I**ntroduction to **C**linical **R**easoning

IHT: **I**terative **H**ypothesis **T**esting

IL: **I**nteractive **L**ectures

ITT: **I**ntention **to** **T**reat

KF_S: Key-Features Tests

MC-items: **M**ultiple **C**hoice **Items**

MCQ: **M**ultiple-**C**hoice **Q**uestions

MI: **M**yocardial **I**nfarction

mini_CEX: The mini-**C**linical **E**valuation **E**xercise

MS: **M**ultiple **S**clerosis

NEJM: The **N**ew **E**ngland **J**ournal of **M**edicine

NICTALOP: NI: the number of key ideas or key diagnoses, **CT**: the choice of terms, **A**: the veracity of the concepts or diagnoses, **LoP**: the length and the position of the ideas and concepts

NSAIDs: **N**on-**S**teroidal **A**nti-**I**nflammatory **D**rug**s**

NSTEMI: **n**on-**ST E**levation **M**yocardial **I**nfarction

OPD: **O**utpatient **D**epartment

OQ: **O**pen **Q**uestions

OSCE: **O**bjective **S**tructured **C**linical **E**xamination

PBL: **P**roblem **B**ased **L**earning

p-PBL: **P**aper **P**roblem-**B**ased **L**earning

PER: pericarditis

PP: **P**er-**P**rotocol

PTE: **P**ulmonary **T**hrombo**e**mbolism

SLE: **S**ystemic **L**upus **E**rythematosus

SNAPPS: **S**ummarize briefly the history and findings, **N**arrow the differential to two or three relevant possibilities, **A**nalyze the differential comparing and contrasting the possibilities, **P**robe the preceptor by asking questions about uncertainties, difficulties, or alternative approaches, **P**lan management for the patient's medical issues, **S**elect a case-related issue for self-directed learning.

SP: **S**imulated **P**atients

sTBL: **S**implified **T**eam **B**ased **L**earning

STEMI: **ST E**levation **M**yocardial **I**nfarction

TAU: **T**eaching **a**s **U**sual

VPs: **V**irtual **P**atient**s**

UTI: **U**rinary **T**ract **I**nfection

VT: **V**entricular **T**achycardia
